# Activity-dependent organization of prefrontal hub-networks for associative learning and signal transformation

**DOI:** 10.1038/s41467-023-41547-5

**Published:** 2023-10-06

**Authors:** Masakazu Agetsuma, Issei Sato, Yasuhiro R. Tanaka, Luis Carrillo-Reid, Atsushi Kasai, Atsushi Noritake, Yoshiyuki Arai, Miki Yoshitomo, Takashi Inagaki, Hiroshi Yukawa, Hitoshi Hashimoto, Junichi Nabekura, Takeharu Nagai

**Affiliations:** 1 Division of Homeostatic Development, National Institute for Physiological Sciences, 38 Nishigohnaka Myodaiji-cho, Okazaki, Aichi 444-8585 Japan; 2 Japan Science and Technology Agency, PRESTO, 4-1-8 Honcho, Kawaguchi, Saitama, 332-0012 Japan; 3 SANKEN (The Institute of Scientific and Industrial Research), Osaka University, Mihogaoka 8-1, Ibaraki, Osaka, 567-0047 Japan; 4 Division of Molecular Design, Research Center for Systems Immunology, Medical Institute of Bioregulation, Kyushu University, 3-1-1 Maidashi, Higashi-ku, Fukuoka, 812-8582 Japan; 5 Quantum Regenerative and Biomedical Engineering Team, Institute for Quantum Life Science, National Institutes for Quantum Science and Technology (QST), Anagawa 4-9-1, Chiba Inage-ku, Chiba, 263-8555 Japan; 6 Department of Computer Science, Graduate School of Information Science and Technology, The University of Tokyo, 7-3-1 Hongo, Bunkyo-ku, Tokyo 113-0033 Japan; 7 Brain Science Institute, Tamagawa University, 6-1-1 Tamagawagakuen, Machida, Tokyo 194-8610 Japan; 8 Instituto de Neurobiologia, National Autonomous University of Mexico, Boulevard Juriquilla 3001, Juriquilla, Queretaro, CP 76230 Mexico; 9 Graduate School of Pharmaceutical Sciences, Osaka University, Yamadaoka 1-6, Suita, Osaka, 565-0871 Japan; 10 Division of Behavioral Development, National Institute for Physiological Sciences, 38 Nishigohnaka Myodaiji-cho, Okazaki, Aichi 444-8585 Japan; 11 Institute of Nano-Life-Systems, Institutes of Innovation for Future Society Nagoya University, Furo-cho, Chikusa-ku, Nagoya, 464-8603 Japan; 12 United Graduate School of Child Development, Osaka University, Kanazawa University, Hamamatsu University School of Medicine, Chiba University, and University of Fukui, 2-2 Yamadaoka, Suita, Osaka, 565-0871 Japan; 13 Division of Bioscience, Institute for Datability Science, Osaka University, 1-8 Yamadaoka, Suita, Osaka, 565-0871 Japan; 14 Open and Transdisciplinary Research Initiatives, Osaka University, 2-1 Yamadaoka, Suita, Osaka, 565-0871 Japan; 15 Graduate School of Medicine, Osaka University, 2-2 Yamadaoka, Suita, Osaka, 565-0871 Japan

**Keywords:** Fear conditioning, Neural circuits, Cortex, Classical conditioning, Prefrontal cortex

## Abstract

Associative learning is crucial for adapting to environmental changes. Interactions among neuronal populations involving the dorso-medial prefrontal cortex (dmPFC) are proposed to regulate associative learning, but how these neuronal populations store and process information about the association remains unclear. Here we developed a pipeline for longitudinal two-photon imaging and computational dissection of neural population activities in male mouse dmPFC during fear-conditioning procedures, enabling us to detect learning-dependent changes in the dmPFC network topology. Using regularized regression methods and graphical modeling, we found that fear conditioning drove dmPFC reorganization to generate a neuronal ensemble encoding conditioned responses (CR) characterized by enhanced internal coactivity, functional connectivity, and association with conditioned stimuli (CS). Importantly, neurons strongly responding to unconditioned stimuli during conditioning subsequently became hubs of this novel associative network for the CS-to-CR transformation. Altogether, we demonstrate learning-dependent dynamic modulation of population coding structured on the activity-dependent formation of the hub network within the dmPFC.

## Abstract (web summary)

Neuronal populations in the prefrontal cortex are involved in associative learning. Here the authors use longitudinal imaging and computational approaches in the mouse prefrontal cortex to observe changes in neuronal ensembles during fear conditioning.

## Introduction

Animals learn to adapt to changing environments for survival. Associative learning, such as classical conditioning, is one of the simplest types of learning that has been intensively studied over the past century^1,2^. It is based on repeated pairings of a neutral conditioned stimulus (CS) such as a tone, and an unconditioned stimulus (US) such as foot shock, that eventually elicits a conditioned response (CR), e.g., freezing response in the associative fear learning paradigm to the CS alone. During the last two decades, technical developments in molecular, genetic, and optogenetic methods have enabled the tagging of a population of neurons in the brain whose specific manipulation allows control of the associative memory^3^. Findings from such studies suggest that information processing by specific neuronal populations is likely to underlie associative memory. How information is stored and processed by the neural population to encode and retrieve the associative memory, however, remains unclear^3^. In addition, although it has been suggested that the formation of associative memory may involve novel associative connections between the originally distinct CS and US networks to enable the CS-to-CR transformation, direct evidence is quite limited.

The prefrontal cortex (PFC) is a brain region that regulates associative fear memory, which is evolutionarily conserved in mammals, from humans to rodents^4–9^. Dysfunction of the PFC may lead to various psychiatric diseases, including post-traumatic stress disorder^10^, and the associative fear learning paradigm has been used as a research model to investigate the underlying mechanisms of this disorder. The dorsal part of the medial prefrontal cortex (dmPFC) of rodents is a brain region demonstrated to be important for the retrieval of associative fear memory^11–16^. During fear memory retrieval and evoked freezing responses (i.e., CR), activated individual neurons^17^ or enhanced synchrony of neural populations^14^ in the dmPFC are observed, while pharmacological or optogenetic silencing of the dmPFC and its projections to specific downstream targets suppresses fear memory retrieval^11,12^, revealing that associative fear memory is normally stored in the dmPFC. Recent studies also uncovered how the dmPFC works together with other brain regions^12,16,18^, including the basolateral amygdala, hippocampus, and paraventricular nucleus of the thalamus, each of which areas have also been intensively studied in the research field of learning and defensive behaviors as well as human psychiatric disorders^3,10,19–22^. Therefore, the dmPFC can serve as an interesting target to address the fundamental question of what structural and computational alterations in the prefrontal networks are required to organize novel associative memories (in the present study, the term “network” describes a functional group of neurons, or a neural population, that contributes to forming an information-processing system). Also, studies of the dmPFC may contribute to our understanding of how novel associative memory is stored in the dmPFC together with pre-existing networks, such as those regulating sensory and motor information.

To address these points, here we developed a pipeline for longitudinal imaging and computational dissection of neural population activities in the dmPFC during fear-conditioning procedures in mice, which enabled us to uncover learning-dependent changes in the internal neural network topology and computation of the dmPFC upon memory acquisition.

## Results

### Fear-conditioning system under the microscope with the head-fixed configuration

**Fig. 1:**
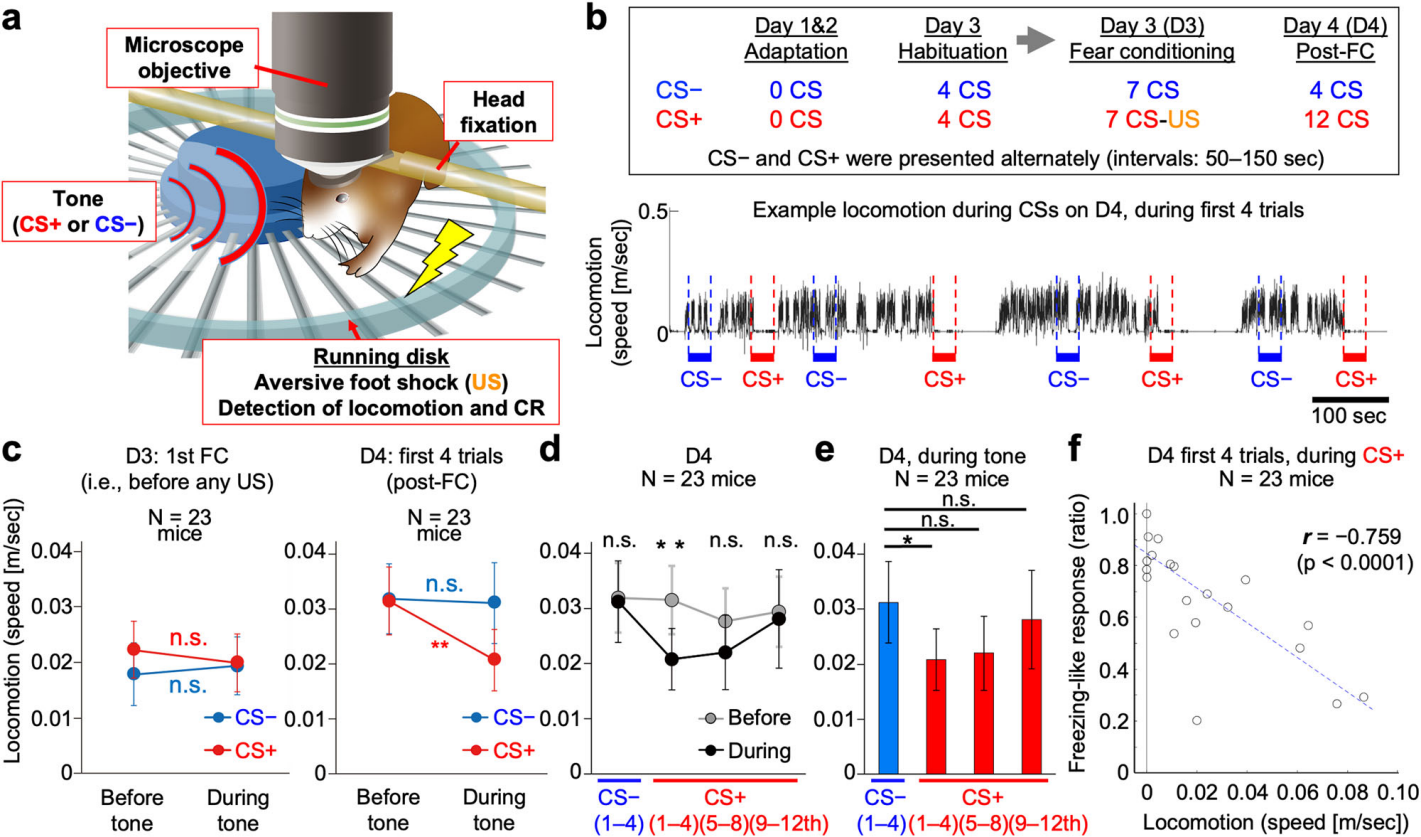
Cued-fear conditioning during two-photon microscopy. **a** Developed system for cued-fear conditioning under a two-photon microscope. **b** (top) Experimental protocol. CS, conditioned stimulus; US, unconditioned stimulus; FC, fear conditioning. (bottom) An example of CS+-evoked changes in the locomotion of a mouse on day [D] 4, the day after fear conditioning. See Supplementary Fig. 1 and Methods for details. **c**, **d** Locomotor speed before the tone onset and during the tone presentation was compared at different experimental phases. During the first 29 sec of the first trials on D3 (i.e., before any CS+-US pairing), mice (*N* = 23) exhibited no significant change during the CS+ and CS− presentations (**c**, left). On D4 (the day after fear conditioning), the CS+ suppressed locomotion as a CR, while the CS− induced no significant change (**c**, right, and **d**). After repeated presentations of the CS+ (5th–12th trials on D4), the CRs became smaller until no significant change in locomotion was observed upon CS+ presentation (**d**). **e** Statistical comparison between locomotion during CS− and that during CS+ at each testing phase on D4. Locomotion during CS+ was significantly lower only during trials 1–4, and not after repeated presentations to the CS+ (5th–12th trials). The same data shown in d (for “during”) are presented for different statistical comparisons. Note that locomotion during the pre-tone-onset (“before”) was not significantly different between the CS− and CS+ conditions. **f** Significant correlation between locomotion and freezing-like response (*p* < 0.0001, Pearson’s correlation test, *N* = 23). Each circle represents an individual mouse. Blue dotted line, linear fitting. Two-tailed tests for all analyses; *p < 0.05; **p < 0.01; n.s., not significant by Wilcoxon signed-rank test. *p* = 0.426 for CS− and *p* = 0.715 for CS+ in **c**-left; *p* = 0.465 for CS− and *p* = 0.0097 for CS+ in **c**-right; *p* = 0.465, 0.0097, 0.101, and 0.670 from left to right in **d**; *p* = 0.021, 0.078, and 0.465 from left to right in **e**; *p* < 0.0001 for **f**. Error bars, s.e.m. Source data are provided as a Source Data file.

To perform longitudinal imaging of neuronal population activities in the dmPFC during fear-conditioning procedures in mice, we first developed a system to perform cued-fear conditioning and memory retrieval while imaging neural activity in the brains of awake and behaving mice with a two-photon microscope (Fig. 1 and Supplementary Fig. 1), which enabled us to record the neural activities of hundreds of neurons with single-cell resolution. The mice were head-fixed under the microscope objective and placed on a running disk. The rotation of the disk was recorded to assess the mouse locomotion state (Fig. 1a) (the term “state” is defined to describe whether a mouse is locomoting, spontaneously stationary, or expressing a CS-induced freezing-like response). Tones and foot shocks were delivered as the CS and US, respectively. Two different tones were used; one was associated with the US (CS+) and the other was not (CS−) (Fig. 1b and Supplementary Fig. 1). We followed the fear-conditioning protocol applied in previous studies using freely locomoting mice^14,15,23^. For example, we used the same number and types of tones and the same interval at each step, except that we used 7 CS+-US pairings rather than the 5 or 6 used in the previous studies and that we used a milder foot shock current (see Methods for details). In the present study, the term “session” is defined as a series of CS presentations with or without a paired US on each day (e.g., a habituation session, a fear-conditioning session, and a post-fear-conditioning [post-FC] session on the day after the fear conditioning), while the term “trial” indicates each 30-sec CS presentation (with or without the US). We also use the term “phase” in the present study to distinguish early and late trials during the same (consecutive) session.

After 2 days of adaptation to the head-fixed system, on day 3 (D3), the mice underwent a habituation session, in which they alternately received 4 presentations of the CS− and CS+ without the US (Fig. 1b and Supplementary Fig. 1a, b). The habituation session was immediately followed by the discriminative fear-conditioning session on the same day, in which the CS+ was paired with the US (Fig. 1b and Supplementary Fig. 1c, d). The US duration was 1 sec and it co-terminated with the CS+ trial. The CS− and CS+ trials were performed alternately (inter-trial intervals, 50–150 s). The next day (day 4, D4), the conditioned mice underwent a post-FC session, in which they received 4 presentations of the CS− and 12 presentations of the CS+ without US presentation (four presentations of the CS− and CS+ trials alternately, followed by 8 CS+ trials; Fig. 1b and Supplementary Fig. 1e, f). Behavioral analyses revealed that the mice learned to exhibit a freezing-like response, i.e., decrease their locomotion as a conditioned response (CR), specifically during the CS+ presentation, only after the fear conditioning (Fig. 1b, c). We refer to this expression of the CS+-evoked CRs during the early post-FC session as memory retrieval^12^. We used 23 naive mice to evaluate our head-fixed system for fear conditioning, while we succeeded with the dmPFC imaging in 11 mice on D3, and 7 of these 11 mice were successfully imaged on both D3 and D4, from the same set of neurons. Compared with the CS+ evoked change in locomotion of the entire cohort mice (Supplementary Fig. 2a), the change in locomotion of the mice used for the longitudinal imaging (explained in the later section) was consistent (Supplementary Fig. 2b). On the other hand, as reported previously^14,15,23^, the CR observed during the early phase on D4 was extinguished after repeated exposure to the CS+ only (Fig. 1d, e) (we refer to this progressive decrease in the CR observed after the repeated CS+-only presentation during the late post-FC session as extinction^14,15,23^). These results indicate the potential usefulness of our system in studying brain computation during fear memory retrieval and following extinction.

To score the freezing-like CR, the locomotion speed of mice was referred to, and if no movement was detected for at least 1 sec, the mouse was considered to be expressing a freezing-like response. With this measure, we also confirmed the significant enhancement of this freezing-like response by the CS+ presentation (Supplementary Fig. 2c). Using Pearson’s correlation and calculating the *r* and *p* value, we confirmed that the locomotion speed was negatively and significantly correlated with the freezing-like response; mice with less locomotion showed more freezing-like responses and vice versa (Fig. 1f).

### Evaluation of the nature of the CR and its dependency on the dmPFC

Prior to investigating neural activities in the dmPFC, we considered two points: (1) whether the CR and the memory retrieval rely on the dmPFC in this behavioral protocol, and (2) whether the CR observed with this system is physiologically similar to the typical freezing response observed in freely moving mice and different from the regular stationary state (i.e., a spontaneous non-locomotive state without CS+). To test the contribution of the dmPFC to memory retrieval in our head-fixed system, we performed chemogenetic silencing of the dmPFC by designer receptors exclusively activated by designer drugs (DREADD). To evaluate the nature of the freezing-like response observed in our head-fixed system, we simultaneously monitored heart rate during the D4 post-FC session (Supplementary Fig. 3a–c) because previous studies suggested that freezing is accompanied by heart rate deceleration in freely moving mice^24^ as well as in other species^25^.

We observed that, as in freely locomoting mice^24^, some control mice exhibited a reduced heart rate when the CS+ was presented and mice were not locomoting in our head-fixed system during the D4 post-FC session, and this heart rate deceleration was accompanied by the suppression of the locomotion (Supplementary Fig. 3d, e). In another case, the mouse showed low-level basal locomotion even without the CS+ (Supplementary Fig. 3f). In this case, while showing no explicit change in the locomotion level by the CS+, the mouse clearly had a reduced heart rate. This observation suggested that the regular stationary state (without CS+) and non-locomotive state during CS+ (i.e., freezing-like response as a CR) might be physiologically different. We further performed the statistical evaluation and confirmed that the heart rate during this freezing-like response under the CS+ presentation was significantly slower than that during the regular stationary state in the control mice (Supplementary Fig. 4a–d).

Importantly, during the CS+ presentation on D4, the heart rates of locomotive mice were significantly faster than those during the CS+-evoked freezing-like response (Supplementary Fig. 4k, right), suggesting the physiological difference between these two states during the CS+ presentation. This observation is essential for the present study since we further utilized the state difference to extract the neuronal population encoding the information for the CS+-evoked freezing-like response. The heart rates without the CS presentation were also similarly enhanced during locomotion; those during the locomotive state were significantly faster than those during the non-locomotive state (Supplementary Fig. 4k, left).

Then, we further tested the role of the dmPFC by specifically targeting the excitatory neurons in the dmPFC for the silencing experiments. We bilaterally injected the adeno-associated virus (AAV) in the dmPFC to express the inhibitory human M4 muscarinic cholinergic Gi-coupled DREADD (hM4Di). The mice were intraperitoneally injected with clozapine-N-oxide (CNO) 30 min before the first trial on D4 (post-FC session; i.e., the day after fear conditioning). On the D4, bilateral silencing of the dmPFC significantly suppressed the CS+-evoked reduction of the heart rate in comparison with the control mice; no significant change was evoked by the CS+ presentation in dmPFC-silenced mice (Supplementary Fig. 4e–h), and there was a significant difference between the dmPFC-silenced mice and non-silenced control mice (Supplementary Fig. 4i, j).

These results suggest that the freezing-like response as a CR observed in our system is physiologically similar to typical freezing in terms of heart rate deceleration, and likely dependent on the dmPFC excitatory neurons which is consistent with the previous studies in freely locomoting mice^11,12^.

Overall, these results demonstrated that our head-fixed system and the fear-conditioning protocol would be potentially useful for observing changes in neural population activity upon associative fear learning.

### Longitudinal imaging of neural population activities in mouse dmPFC during fear-conditioning procedures

**Fig. 2:**
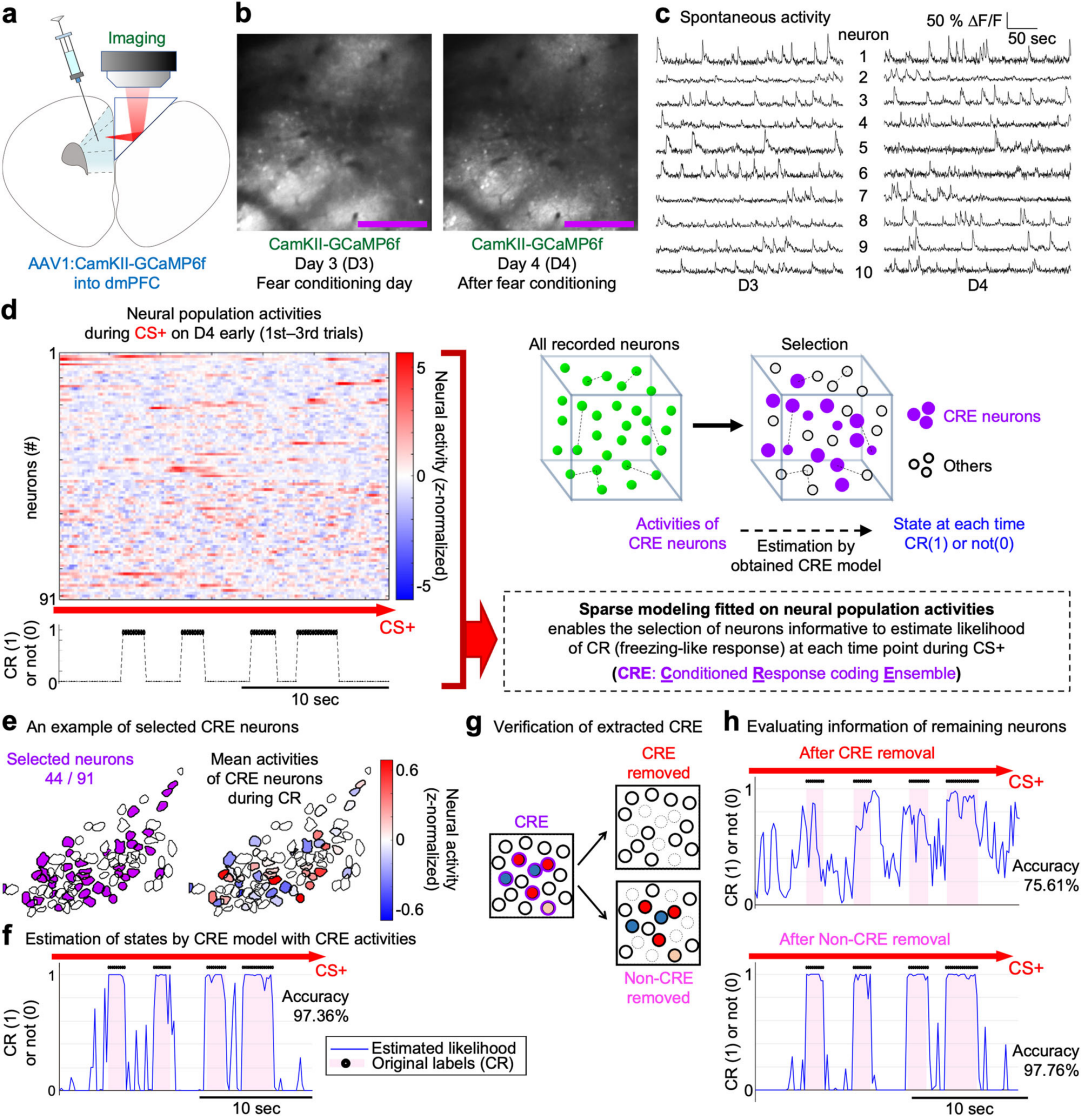
Longitudinal in vivo imaging in dmPFC and extraction of the CR ensemble. **a** Microprism implantation along the midline for optical access to the dmPFC without cutting nerves. GCaMP6f was expressed in the dmPFC excitatory neurons by the AAV under the CamKII promoter regulation. **b** In vivo two-photon microscopy to detect activities at the single-cellular resolution visualized by GCaMP6f, chronically (day [D] 3 and D4) from the same set of dmPFC neurons. See also Supplementary Movies 1 and 2. Scale bar, 250 μm. **c** Longitudinal detection of spontaneous Ca^2+^ activities on D3 and D4 from 10 example dmPFC neurons. **d** Extraction procedure for the CR ensemble (CRE). See the Methods for details. **e** An example of the extracted CRE. (left) Selected neurons. (right) Mean neural activity during CR (freezing-like response) is shown in color. **f** Time-course changes of neural representation encoded by the CRE (of the one shown in **e**) under the CS+ presentation on D4 (memory retrieval phase). The plots show a part of the whole length of the data (during the CS+ presentation). Overall estimation accuracy was 97.36% in this example. **g** Schematic diagram showing how the extracted CRE neurons (circled by the purple lines) were verified through comparison of the fitting performances between CRE removed (when all CRE neurons were removed) and Non-CRE removed (when Non-CR ensemble [Non-CRE] neurons were removed). The fitting performance by the “CRE removed” should be substantially decreased compared with the “Non-CRE removed” if most of the neurons informative for the CR are sufficiently selected as the CRE. **h** An example of the comparison of the fitting performances, revealing the poor remaining information in the “CRE removed” (in the same mouse analyzed in **e** and **f**). **d**, **f**, **h** black dots on the top of the graphs and pink color indicate the timing of the actual CR, while blue lines show the likelihood of locomotion states estimated by the activity of the respective neural populations. As a neural activity, ΔF/F (**c**) or z-normalized ΔF/F (**d** and **e** are shown).

Next, to monitor the neural activities in the dmPFC by two-photon microscopy, we implanted a 2-mm microprism along the rostral midline of the brain to optically access the dmPFC region. Although the size of the prism was larger than that of prisms used in previous work^26^, there was sufficient space and no callosal fibers between the hemispheres around the dmPFC area, especially at the rostral region, enabling smooth insertion of the prism without cutting prefrontal or callosal neural fibers (Fig. 2a). Using a genetically encoded Ca^2+^ indicator, GCaMP6f, expressed by an AAV, the activities from a wide region of the prefrontal area were chronically visualized (Fig. 2b, c and Supplementary Movie 1). To specifically record the activity of the excitatory neurons^27^ and separate them from inhibitory neurons that may have a distinct function in the dmPFC^13^, the GCaMP6f was expressed under the regulation of the CaMKII promoter^28,29^. The CS and US presentation did not disturb image acquisition (Supplementary Movie 2). We focused on analyzing the activities of the surface layer neurons (~150–200 μm depth below the pial surface along the midline) in the dmPFC area (see the Methods for details). In most of the data analyses, the neural representation during the first three trials of the fear conditioning on D3 (D3-early) was compared with those during the first 3 trials on D4 (D4-early) to assess the changes occurring as a result of the fear conditioning. The data obtained during the last 3 trials on D3 (D3-late) were used to assess the late conditioning phase, and the data obtained during the last 3 trials on D4 (D4-late) were used to assess the extinction phase.

Prior to investigating population coding in the dmPFC, we assessed single-neuron responses to the CS+ and CS− before and after the acquisition of the fear memory (Supplementary Fig. 5; *n* = 1165 neurons from *N* = 7 chronically recorded mice; for each mouse, *n* = 91, 116, 249, 99, 288, 157, 165 neurons respectively). We found that ~60% of neurons exhibited a change in neural activity following exposure to the CS+ and/or CS−, and ~20% of neurons showed responses to both the CS+ and CS−. The distributions of these types of neurons were consistent throughout the learning process (Supplementary Fig. 5d). This type of responsiveness of individual neurons to variable task-relevant aspects has also been reported in the primate PFC^30^, and is proposed to enhance the number of tasks that each neural circuit containing a limited number of neurons can handle in a high-dimensional space implemented by a population of networked neurons^30,31^. This encouraged us to further analyze the population coding for associative fear memory, which was followed by the comparison with the single-cellular responsiveness.

### Extraction of the neuronal ensemble encoding the CR

To dissect the computational architecture composed by a neural population in the dmPFC implementing a novel associative memory, we next extracted a group of neurons encoding the freezing-like conditioned response (named the CR ensemble; CRE in figures) based on the neural population activities during D4-early (i.e., memory retrieval phase) (Fig. 2d–f), and further analyzed the features of the extracted ensemble to investigate the change induced after fear conditioning. In addition, we aimed to elucidate whether the CR-coding ensemble and the regular motor-coding ensemble overlapped or were distinct from each other.

We used the elastic net^32^, a model-based regularization algorithm that enabled us to select the neural population corresponding to the CR as well as to independently extract the motor-coding ensemble for comparison. *L*^1^-regularization algorithms such as LASSO also allow the selection of the neural population, but if there is a group of highly correlated neurons, *L*^1^ regularization tends to select only 1 neuron from the group and ignore the others^32^ (see the Methods for details). Importantly, neuronal activity in the cortical network is correlated^14,33^, and, as we describe below, a substantial number of correlated pairs was observed in our recording data. On the other hand, the elastic net that we use in the present study is formulated as the combination of *L*^1^ and *L*^2^ regularizations, can perform better for the regression and classification problems by balancing these regularizations, and is advantageous to avoid missing such correlated neurons during the selection^32^ (see the Methods for details).

Models were fitted on neural population activities to estimate the likelihood of locomotion state at each time point. Sparse models with high fitting performance (CR-ensemble model for neural population data of each mouse) were produced by this procedure (Fig. 2d). Since the sparse model estimates or predicts the mouse locomotion states by referring to the activities of a limited number of neurons (among the entire recorded neurons), this method enabled us to select neurons that were informative for estimating the corresponding locomotion state, the CR.

We extracted the CR ensemble using the elastic net by referring to population neural activity data recorded during the CS+ presentation of D4-early (Fig. 2d, e). The fitting performance (ratio of correct estimation) of the obtained model was calculated to evaluate the model. We demonstrated that the CR of the mice could be estimated at a high accuracy using the obtained model and the neural activities of these selected CR ensemble neurons (Fig. 2f). When using the elastic net, the degree of inclusion of correlated pairs can be adjusted by the hyperparameter “alpha”^32^. We systematically tested a wide range of alpha values (Supplementary Fig. 6) and evaluated whether informative neurons were left as unselected neurons at each alpha by measuring the information (fitting performance of a model) of the remaining and unselected neurons (Fig. 2g, h, and Supplementary Fig. 6; see also Methods). This procedure enabled us to confirm whether the neurons informative for estimating the CR were maximally extracted as a result of the optimization of the alpha.

This systematic optimization procedure revealed a general trend that a larger alpha tended to select a smaller number of neurons as the CR ensemble (Supplementary Fig. 6b, top), as expected from the general feature of the elastic net. Interestingly, the fitting performance by the small CR ensemble was quite high, equivalent to the fitting performance by the larger CR ensemble (Supplementary Fig. 6b, middle), while the removal of such a small portion from the whole set of neurons was not always sufficient to substantially diminish the information left in the remaining neurons (Supplementary Fig. 6b, top and bottom, and d–f). This suggests that the CR was redundantly encoded in the dmPFC at the level of the population coding (a detailed discussion of this redundancy is provided in the Discussion).

After determining the optimal alphas for individual circuits (i.e., each group of neurons simultaneously observed in the individual mice), we observed a substantial reduction of the fitting performance when all the neurons selected for the CR ensemble were removed (Fig. 2h, and Supplementary Fig. 6), confirming that a sufficiently large portion of the dmPFC neurons encoding the CR was selected as the CR ensemble by our method.

**Fig. 3:**
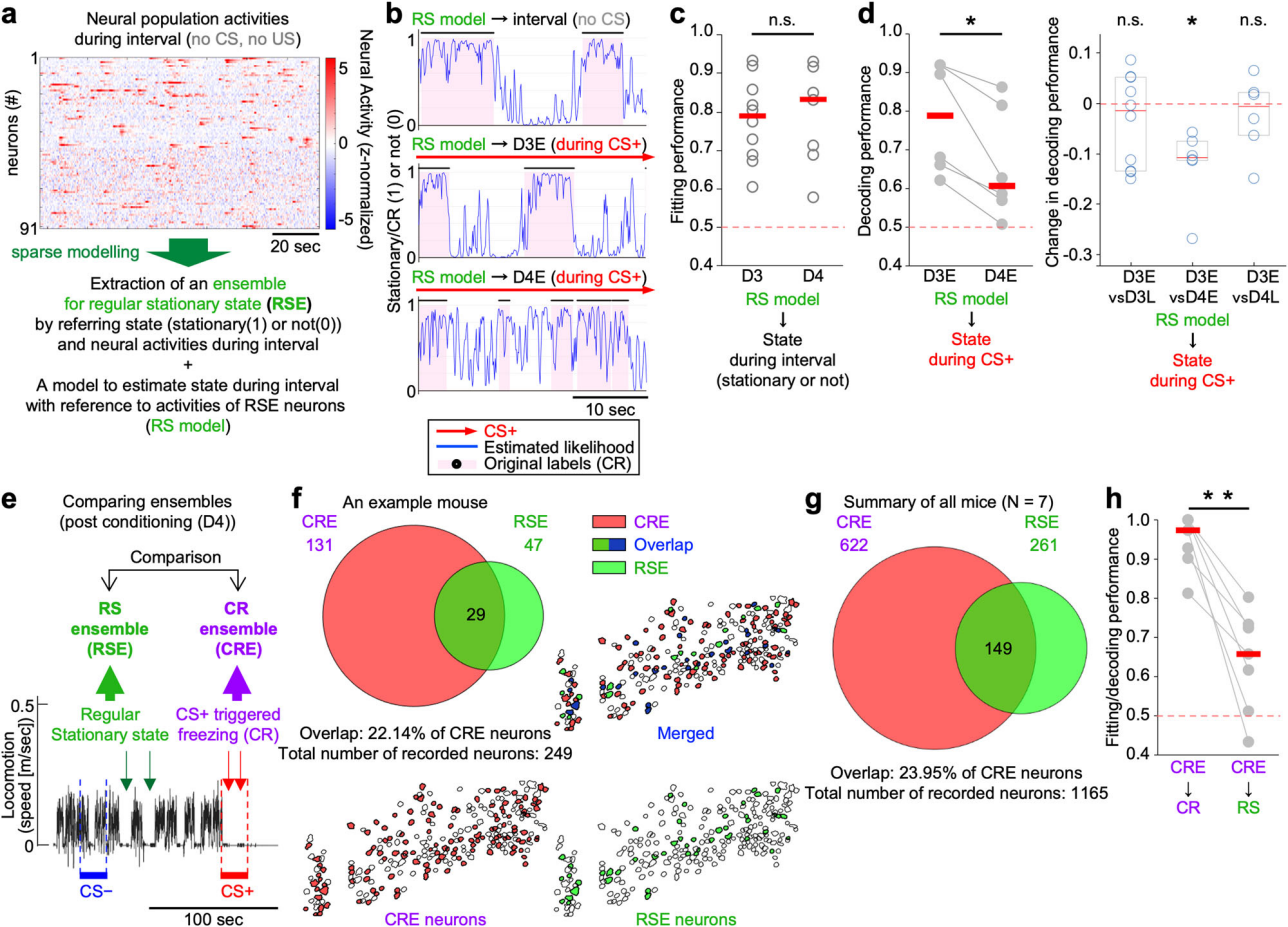
Emergence of the unique CR ensemble after fear conditioning. **a** Schematic diagram for extracting the RS ensemble (RSE) with building the RS model. As a neural activity, z-normalized ΔF/F is shown. **b–d** Estimating and decoding locomotion states by the RS model. **b** In an example mouse, the RS model possessed high performance for estimating RS (day[D]4-interval; top) and decoding locomotion states during CS+ at D3-early (D3E; middle). However, the performance decreased for the locomotion states during CS+ at D4-early (D4E; bottom). **c** No significant difference in the original RS-model performance between D3 and D4. **d** (left) Significant decrease in the RS-model decoding performance for locomotion states during CS+ at D4E, compared with D3E. (right) The changes in the decoding performance visualized by subtracting D3E values from others (D3-late [D3L], D4E, D4-late [D4L])). **e** Schematic diagram for comparing the overlap between the CR ensemble (CRE) and the RSE. **f** A Venn diagram and a spatial map of an example mouse showing the limited overlap between the CRE and RSE. **g** Summary of the overlap between the CRE and RSE of all 7 mice (*n* = 1165 neurons). **h** The decoding performance of the CR model to the RS was statistically compared with the fitting performance of the CR. Of 11 successfully imaged mice, 7 mice were longitudinally imaged on D3-D4. Because data from 1 of the 7 mice on D3 did not meet the RS-modeling criteria, *N* = 10 for D3 (**c**, **d**); *N* = 6 for D3-D4 paired comparison (**d**); *N* = 7 for D4 (**c** and Supplementary Fig. 8). The CR models (at D4E) were successfully built in all seven mice (**h**). The fitting/decoding performances are indicated by the accuracy, while the AUC was similar (Supplementary Fig. 8). Two-tailed, Wilcoxon rank-sum test (**c**, *p* = 0.887) and paired permutation tests (**d**-left, *p* = 0.016; **d**-right, *p* = 0.244, 0.016, 0.531 from left to right; **h**, *p* = 0.008) were used. *p < 0.05; **p < 0.01; n.s., not significant. Red bars, median; box in **d**-right, 25th–75th percentiles. Source data are provided as a Source Data file.

We eventually confirmed that the CR ensemble obtained by the optimal alpha was highly informative for estimating the CR during D4-early (mean ± SE of the estimation accuracy, 0.9450 ± 0.0265, *N* = 7 mice; see an example case shown in Fig. 2f; the summary of the individual data is shown later in Fig. 3h [“CRE to CR”]). As for the spatial distribution, the CR ensemble was spatially intermingled in the field of view, as shown in Fig. 2e.

### The CR ensemble is distinct from the regular motor-coding ensemble

We then evaluated the specificity and uniqueness of the extracted CR ensemble. For this purpose, we independently extracted a group of regular motor-coding neurons (named regular stationary state [RS] ensemble; RSE in figures) using the elastic net for comparison (Fig. 3a). To extract the RS ensemble, instead of the population neural activity data during the CS+ presentation that were used to extract the CR ensemble, we used the population neural activity data recorded during the no-CS period (i.e., whole-day data, including the period prior to the CS presentation and that during the inter-trial interval; see the Methods for details). Interestingly, the selection of the RS ensemble was not clearly affected by the alpha values (Supplementary Fig. 7). This suggests a possible difference in the coding structure between the CR ensemble and the RS ensemble, and that the RS was less likely to be redundantly encoded in the dmPFC at the level of the population coding.

The RS model, a model for estimating the RS based on the neural population activities of the RS ensemble (Fig. 3a), showed high performance not only for estimating the locomotion state during the no-CS period (RS model to interval (no CS) in Fig. 3b, top, and c), but also for decoding the locomotion state during the CS+ presentation on D3, i.e., during the early phase of the fear conditioning (RS model to D3-early [during CS+] in Fig. 3b, middle, and d). This result suggested that the RS model was applicable to the data obtained when CS+-related activities were also observed (as shown in Supplementary Fig. 5), and thus, like cross-validation by the data with the additional noise, confirmed the reliability of the RS model obtained using our elastic net-based method. No significant change in the decoding performance was observed during the fear conditioning (D3-early vs D3-late; Supplementary Fig. 8).

The fitting performance for the RS estimation by the RS model was also similar between D3 and D4 (i.e., the day of the fear conditioning vs the day of the post-FC session) (Fig. 3c). The decoding performance of the RS model to the locomotion state during the CS+ on D4-early, i.e., during memory retrieval, was significantly reduced, however, compared with that of D3-early (RS model to D4-early [during CS+] in Fig. 3b, bottom, and d; results of the detailed analyses are also summarized in Supplementary Fig. 8). These results suggest that the locomotion states during the memory retrieval, or the CR, could not be explained by the RS model. We also found that most of the neurons selected as the CR ensemble were unique and did not overlap with the RS ensemble (Fig. 3e–g). We further observed that the CR model was specific to the CR (the locomotion state during D4-early under CS+ presentation) and not applicable to the RS (locomotion state during no CS) on D4 (Fig. 3h). These results suggest that the CR ensemble extracted by our method based on the elastic net was unique, not the simple motor-coding population, and dominantly and exclusively explained the locomotion states of the mice during the CR (i.e., CS+-evoked memory retrieval) as an encoder of the acquired associative memory.

As widely introduced in the cued-fear conditioning paradigm^34–36^, we used different floor (i.e., running disk) textures for D3 and D4 to change the context (see the Methods for details). One might consider this difference to be a causal factor of the difference in the decoding performance between D3-early and D4-early shown in Fig. 3b, d (RS model to locomotion states during CS+). Importantly, however, this reduced decoding performance at D4-early (i.e., memory retrieval phase) was substantially recovered on the same day with the same running disk at D4-late (i.e., extinction phase; no significant difference between D3-early and D4-late, and a significant difference between D4-early and D4-late; Fig. 3d, right, and Supplementary Fig. 8), suggesting that the contextual difference was not the causal factor of the reduced decodability at D4-early.

These results established that the CR, or the locomotion state occurring as a result of memory retrieval, was dominantly explained by the CR ensemble, supporting the idea that the CR ensemble systematically extracted by our method was a dominant and specific group of neurons encoding the CR that emerged on the day after the fear conditioning during the memory retrieval phase and was suppressed during the extinction phase.

### Coactivity within the CR ensemble is specifically enhanced after fear conditioning

In the CR ensemble, we observed a slight but significant increase in CS+ activatable neurons, but no change in CS+ inactivated neurons after fear conditioning (Supplementary Fig. 9a–d). In contrast, other cells (Non-CR ensemble: neurons that were not extracted as the CR ensemble; Non-CRE in figures) exhibited no significant changes in the CS+ activatable neurons, with a significant increase in CS+ inactivated neurons. Neurons in the RS ensemble did not exhibit any significant change in CS+ responsiveness. We detected no significant change in CS− responsiveness in any of the categories. Because the CR ensemble was discriminated by the neural activity data and locomotion states during the CS+, not by comparisons between those during the CS+ and those during the presentation of other stimuli or the interval, our method produced no bias toward the CS+ during the selection of the CR ensemble.

In addition to the analyses based on the number of CR ensemble neurons responsive to the CS+, we investigated the characteristics of the extracted CR ensemble neurons by further analyzing the activity level of these neurons at different phases and different experimental days. When we looked at the responsiveness of individual neurons, some of the neurons activated by the CS+ (“CS+ activated neurons”) in the CR ensemble neurons showed higher responses during the D4-early compared with the D3-early (Supplementary Fig. 9e, f). We further statistically tested whether they showed differences in responsiveness at different phases (D3-early, D3-late, D4-early, D4-late). Comparison between D3-early and later phases suggested that the CS+-activated CR ensemble neurons showed significantly higher responses to the CS+ during D4-early (Supplementary Fig. 9g). This enhancement declined during the D4-late (Supplementary Fig. 9g). At D3-late, while comparison between the groups showed no significant difference from the D3E, a subset of neurons showed higher responses than most of the neurons at D3-early, which was indicated by the higher top 25 percentile line (gray boxes in Supplementary Fig. 9g). These observations for the responsiveness of individual neurons suggest the existence of a mechanism by which fear conditioning (or repeated CS-US pairings) results in the CS+ dominantly activating a subset of dmPFC neurons that also encode the CR.

To elucidate the mechanism underlying associative learning and memory at the neural population level, we measured the coactivity in the extracted CR ensemble of each mouse during the CS+ presentation by calculating pairwise correlation coefficients^33^. The pairwise correlation coefficients are widely used to evaluate neural population activity and are reportedly related to improved or impaired network computation^33,37–39^. We investigated the changes that occurred as a result of fear conditioning and found that only the positively correlated fraction was enhanced after the fear conditioning specifically within the CR ensemble, and not in the outside network (Non-CR ensemble) (Supplementary Fig. 10a). Statistical analyses demonstrated that this enhancement in positive correlation after the fear conditioning, as well as the enhanced ratio of significantly and positively correlated pairs, specifically occurred in the CR ensemble (Supplementary Fig. 10a–c).

We also performed analyses based on shuffled datasets, as described in previous studies^33,40^ to consider the possibility that a change in basal activity may contribute to the change in the correlation coefficient. Analyses based on the shuffled data, where the activity of each neuron was preserved but the temporal order was randomly shuffled neuron by neuron, revealed no significant difference between the CR ensemble and Non-CR ensemble (Supplementary Fig. 10a, c), suggesting that the specific enhancement of the coactivity of the CR ensemble in the real data did not derive from the enhanced basal activity.

A similar enhancement of the coactivity was observed in the CR ensemble excluding the RS ensemble-overlapped neurons (Supplementary Fig. 10a–c). In addition, changes in the coactivity across the categories (coactivity between the CR ensemble and the Non-CR ensemble neurons) were significantly smaller than those within the CR ensemble (Supplementary Fig. 10c). These results led us to hypothesize that the functional connectivity within the CR ensemble was specifically enhanced as a result of the fear conditioning, contributing to enhancing the coactivity.

### Enhanced internal connectivity and association with the CS in the CR ensemble after fear conditioning

**Fig. 4:**
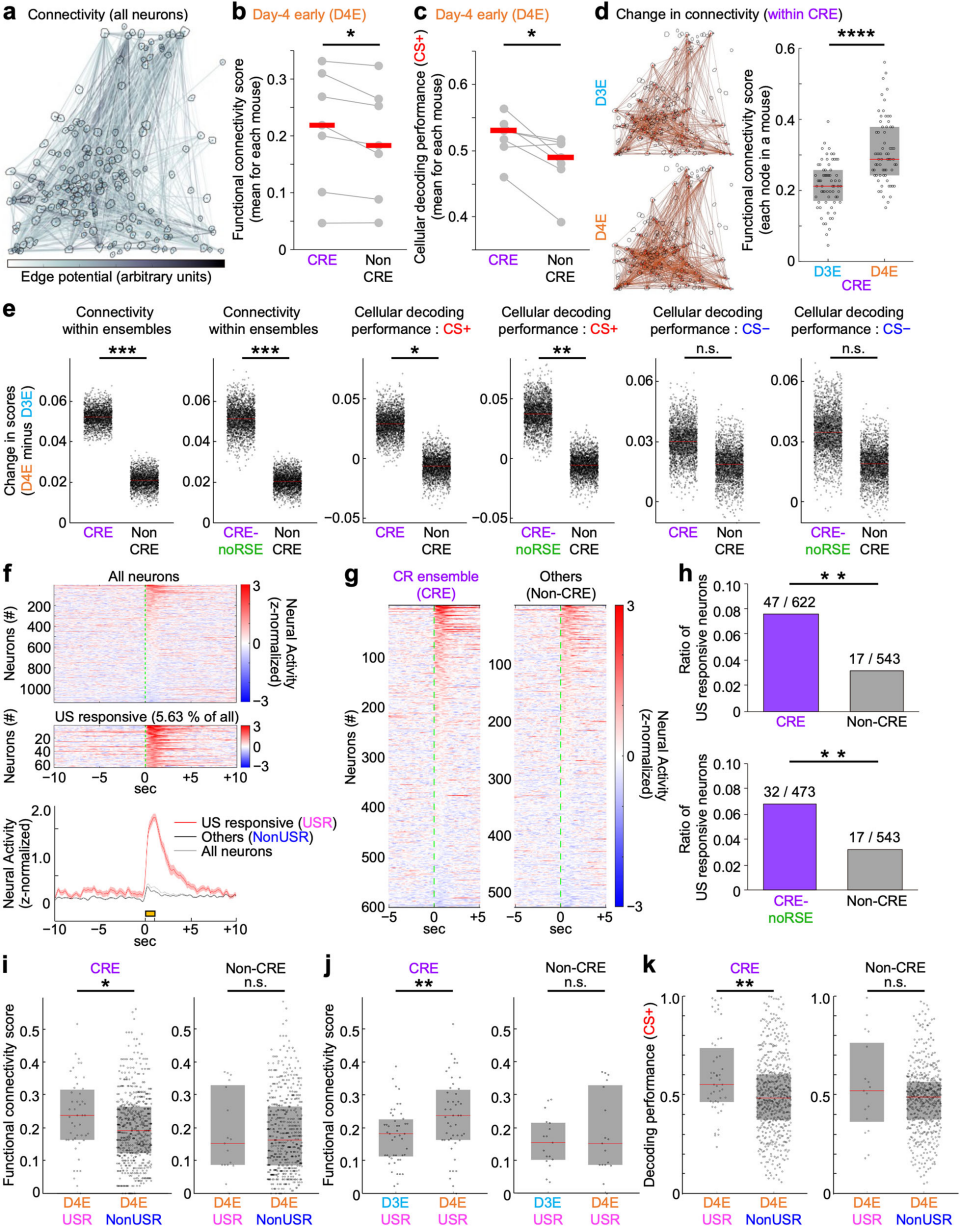
Enhanced functional connectivity and CS+ predictability in the CR ensemble with an emergent hub of US-responsive neurons after fear conditioning. **a** Functional connectivity in an example neuronal circuit, estimated by the CRF model. Top 50% edge potentials were visualized. **b** Higher functional connectivity within the CR ensemble (CRE) compared with the others (Non-CRE) during the day[D]4-early (D4E). **c** Higher decoding performance for CS+ in CRE compared with Non-CRE during D4E. **d** Enhanced functional connectivity within the CRE in an example circuit after fear conditioning (cf., **a**; *n* = 67 CRE neurons marked by red ellipses [left, spatial maps] or black dots [right, functional connectivity scores]). **e** Changes in functional connectivity and cellular decoding performance (for CS+ and CS−) in CRE, CRE-noRSE (CRE excluding those overlapping with RS ensemble), or Non-CRE, evaluated by calculating D4E-D3E differences (*N* = 7 mice, 2000 times bootstrap resampling). **f**, **g** dmPFC neuronal responses to the US on D3. Neural activities (mean over seven trials) were aligned at the US onset ordered by the magnitude of response for 1.5 sec from the US onset. As neural activity, z-normalized ΔF/F is shown. Green dotted lines, US onset; yellow bar, 1-sec US presentation. **f** (top) All neurons. (middle) US-responsive neurons (USR). (bottom) Mean ± s.e.m. of respective categories. **g** US responses of CRE or Non-CRE. **h** US-responsive neurons on D3 were predominantly involved in the CRE or CRE-noRSE on D4. **i**–**k** Comparison of functional connectivity scores (**i**–**j**) and CS+-decoding performances (**k**) between USR and others (NonUSR) at D4E (**i**, **k**), or between D3E and D4E (**j**), either within the CRE or Non-CRE. *N* = 7 mice in Fig. 4 (except for **a**, **d**). Two-tailed, paired permutation test (**b**, *p* = 0.016; **c**, *p* = 0.016), bootstrap resampling-based analysis (**e**: *p* < 0.001, < 0.001, 0.013, 0.006, 0.411 and 0.270, left to right), Fisher’s exact test (**h**: top, *p* = 0.001; bottom, *p* = 0.008), Wilcoxon rank-sum test (**i**: CRE, *p* = 0.020; Non-CRE, *p* = 0.999; **k**: CRE, *p* = 0.001; non-CRE, *p* = 0.350), and Wilcoxon signed-rank test (**d**: *p* < 0.0001; **j**: CRE, *p* = 0.003; Non-CRE, *p* = 0.554) were used. *p < 0.05; **p < 0.01; ***p < 0.001; ****p < 0.0001; n.s., not significant. Red bars, median; boxes in **d**, **e**, **i**–**k** indicate 25th–75th percentiles. See Methods and Source Data for details.

To test the hypothesis above, we introduced a probabilistic graphical model method, the conditional random field (CRF) model^41,42^. This method evaluates the contribution of specific neurons to the overall network activity by modeling the conditional probability distribution of a given neuronal population firing together or of a suppressive relationship (see the Methods for details). We generated a graphical model in which each node represents a neuron in a given neural population and edges represent the dependencies between neurons, which enabled us to estimate the functional connectivity between dmPFC neurons that were simultaneously recorded (Fig. 4a). Among the various mathematical algorithms used to evaluate the possible functional connectivity of neural networks and ensembles, the CRF model is one of the most reliable methods because the results of the calculation (functional connectivity) have already been carefully evaluated by two-photon holographic optogenetics and consequential behavioral modulation^41,42^. In the present study, we calculated the ratio of these relevant connections (both coactive and suppressive) per all possible connections for each node as a “functional connectivity score” for each neuron (see the Methods for details).

Using this method, we found that, after the fear conditioning (D4-early), the functional connectivity was significantly higher in the CR ensemble (Fig. 4b). This method also allowed us to evaluate the information coding of any arbitrary label, e.g., CS+ or CS−, and we found that the CS+ information encoded by the CR ensemble was significantly higher than that of the Non-CR ensemble (Fig. 4c). Importantly, our method did not produce any bias to the CS+ in selecting CR ensemble neurons, as explained above. Therefore, this result indicates that the neural population encoding the CR was dominantly associated with the CS+ information in the post-conditioning dmPFC network. In addition, we found that the enhancement in both the functional connectivity and CS+ predictability was experience-dependent and derived after the fear conditioning, dominantly in the CR ensemble neurons (Fig. 4d, e). In contrast, the changes in information coding for the CS− were not significantly different between the CR ensemble and the Non-CR ensemble (Fig. 4e). Therefore, the emergence of the CR ensemble after fear conditioning was accompanied by enhancement of the internal coactivity (Supplementary Fig. 10), functional connectivity, and an association with the CS+ selectively within the CR ensemble. These results indicate that fear conditioning drives a reorganization of functional connectivity in the dmPFC, which may lead to the formation of an information-processing neural network to trigger the CR by the CS+ (i.e., a neural network for the CS+-to-CR transformation).

### US-responsive neurons during fear conditioning subsequently become hubs of the CR ensemble

Finally, we hypothesized that the functional reorganization that we observed in the dmPFC after fear conditioning occurs via activity-dependent modulation during the repeated CS+-US pairing. This led us to search for the signature of this plasticity.

During the fear conditioning, we observed that some of the dmPFC neurons strongly responded to the US (Fig. 4f). Interestingly, the total number of US-responsive neurons during the fear conditioning (D3) was 64, while 47 became included in the CR ensemble, suggesting that 73.44% of the neurons responding to the US during the fear conditioning became integrated into the CR ensemble. The statistical analyses demonstrated that neurons responsive to the US during fear conditioning were predominantly and significantly more involved in the CR ensemble after the fear conditioning (Fig. 4g, h). Similarly, we evaluated the CS responsiveness of the neurons becoming the CR ensemble. During the D3-early (i.e., the early phase of the fear conditioning), the total number of CS+-responsive neurons was 495, while 278 became included in the CR ensemble, suggesting that 56.16% of the neurons responding to the CS+ during the D3-early became integrated into the CR ensemble. We similarly evaluated the CS−-responsiveness, and found that the ratio for the CS− is 55.43%. During the D3-late, the ratio for CS+ or CS− was 57.65 or 56.44 % respectively, which are slightly higher than those at D3-early. Also, all of the ratio values are smaller than that of the US-responsive neurons. Further statistical analyses revealed that neural responses to the CS+ during D3-early were not significantly implicated in whether the neurons became included in the CRE on the day after fear conditioning (Supplementary Fig. 11a, left). On the other hand, neurons responding to the CS+ during D3-late (i.e., after the repeated CS+-US pairing) were predominantly involved in the CRE on D4, which was statistically confirmed (Supplementary Fig. 11b, left), though the difference between the CRE and the Non-CRE was smaller than the case of the US responsiveness (Fig. 4h). There was no statistical significance in the case of CS− (Supplementary Fig. 11a, b, right panels). These results suggest that the US-responsive neurons (shown as USR in figures) were preferably integrated into the CR ensemble, in which functional connectivity might also be modulated and strengthened by US-evoked activity, perhaps together with the paired CS+ responsive neurons.

To test this possibility, we performed further analyses based on the CRF modeling. We found that the US-responsive neurons became functionally more connected within the CR ensemble than the Non-US-responsive neurons (i.e., neurons that were not defined as US-responsive neurons; NonUSR in figures), while these differences were not observed in the Non-CR ensemble (Fig. 4i). This higher connectivity was a result of the fear conditioning (Fig. 4j). The information coding for the CS+ was also significantly higher in the US-responsive neurons, specifically in the CR ensemble (Fig. 4k). As expected from this enhanced information coding for the CS+, we also observed that the functional connectivity between the US-responsive neurons and the neurons activated by the CS+ was significantly enhanced within the CR ensemble as a result of the fear conditioning (Supplementary Fig. 12a), while there was no significant difference for the neurons inactivated by the CS+ (Supplementary Fig. 12b). These results suggest that the US-responsive neurons were dominantly associated with the CS+-activated neurons when they became integrated into the newly emerged CR ensemble.

According to a previous study, higher functional connectivity and higher decoding performance of sensory stimuli are typical features of pattern-completion cells whose activation could efficiently enhance the entire ensemble activity for a specific sensory stimulus and promote the stimulus-associated behaviors of mice^41^. In the present study, we observed that the CR ensemble neurons became more activated by the CS+ as a result of the fear conditioning (Supplementary Fig. 9). The functional connectivity between the CS+-activated neurons and the US + -responsive (activated) neurons was enhanced as a result of the fear conditioning (Supplementary Fig. 12). The information coding for the CS+ in such US-responsive neurons was also enhanced in the CR ensemble but not in the outside network (Non-CR ensemble) (Fig. 4k). We also observed that the US-responsive neurons possessed significantly higher functional connectivity within the CR ensemble than the other neurons (Fig. 4i), of which enhancement was experience-dependent (Fig. 4j). The CR ensemble encoded the CR information and was distinct from the regular motor-coding neurons (Figs. 2 and 3). Conclusively, these results visualized the possible signal flow from the CS+ to the CS+-activated neurons in the CR ensembles, then to the US-responsive neurons, and further to the entire CR ensembles, most of which association or responsiveness was enhanced as a result of the associative fear learning paradigm. In other words, the US-responsive neurons in the dmPFC gained features of pattern-completion cells and became a hub of the novel neural ensemble linking the CS+ to the CR, a memory-evoked response, after the repeated CS+ and US pairings.

## Discussion

In the present study, we developed a pipeline for longitudinal two-photon imaging and computational dissection of the neural population, which allowed us to investigate learning-dependent dynamic modulation of population coding for associative fear learning, structured on an activity-dependent hub-network formation within the dmPFC. We observed that the repeated CS+-US pairing for the associative learning encouraged dmPFC reorganization characterized by adaptive rearrangement of the functional connectivity within a specific subset of dmPFC neurons and promoted the development of the unique CR-coding neural network distinct from the regular motor-coding neural networks in the dmPFC. This functional reorganization within the dmPFC was accompanied by enhanced internal coactivity, functional connectivity, responsiveness to the CS+, and information coding for the CS+, which were enhanced as a result of the fear conditioning. Upon this prefrontal reorganization, neurons activated by the US during fear conditioning were subsequently and predominantly integrated into the CR ensemble. Detailed analyses combining traditional measures based on the single-cellular responsiveness with the CRF graphical modeling technique proposed a possible signal flow in the extracted CR ensemble during the memory retrieval, which may allow the signal transformation from the CS+ to the CR via the US-responsive neurons. The eventual network stemming from these US-responsive neurons gained typical features of pattern-completion cells of the CR ensemble, which are supposed to work as a hub in the dmPFC to predominantly relay the CS+ information and promote the CR (Supplementary Fig. 13).

The comparisons between D4-early (D4E) and D4-late (D4L), i.e., memory retrieval and extinction phases also support the emergence and the temporal specificity of the extracted CR ensemble. We observed that the reduced decoding performance at D4-early by the RS model, a model to predict the regular stationary state, was substantially recovered at D4-late (Fig. 3d and Supplementary Fig. 8). Also, we additionally demonstrated that the mean activity level of the CS+-activated neurons in the CR ensemble once became significantly enhanced during the D4-early (compared with the D3-early), but this enhancement declined during the D4-late (Supplementary Fig. 9g). These results support that the CR ensemble was a dominant and specific group of neurons encoding the CR, emerged on the day after the fear conditioning, was activated during the memory retrieval phase, and was suppressed during the extinction phase.

A previous study investigated the population coding by the dmPFC neurons during memory retrieval using the electrophysiological recording technique^16^, reporting that the dmPFC neural population encodes information related to the CS+ and the upcoming CS+-triggered defensive behavior (avoidance behavior), silencing of which suppresses this memory-driven defensive response. In the study, how the population coding emerged was not addressed, which could be advantageously addressed by the two-photon-based longitudinal imaging techniques instead. Also, other recent studies reported that the assimilation of US activity with the CS occurred in the dmPFC^18,43^. But the direct relationship between such emergent CS-US assimilation of the neural representation and the information coding by the neural population during the memory retrieval remained unclear.

The novelty of the present study is that we first extracted the specific subpopulation of dmPFC neurons that encoded the CR and emerged as a result of the fear conditioning, of which specificity and distinction from the regular motor-coding population were confirmed with the advantage of the longitudinal two-photon imaging, and the elastic net. The regularized regression method (elastic net) allowed us to systematically extract the exact neurons that were dominantly and sufficiently encoding the CR, and distinguish them from the regular motor-coding ensemble. We demonstrated that the enhancement of the functional link between CS+ and US-responsive neurons was specifically developed in the CR-coding subpopulation, named the CR ensemble in the present study, as a result of the fear conditioning. Furthermore, the combination of the conventional measures based on the single-cellular responsiveness and the new CRF graphical modeling technique based on the analyses of coactivity in the neural population contributed to investigating the possible signal flow and its experience-dependent dynamic change in the dmPFC which may underly the CS+-to-CR signal transformation.

To our knowledge, this is the first in vivo evidence revealing (1) the emergence of a neural population encoding the CR (CR-ensemble) in the dmPFC as a result of CS+-US pairing, (2) that the CR-ensemble is distinct from the regular motor-coding ensemble, (3) that the emergence of the CR-ensemble in the dmPFC is based on an enhanced association (functional connectivity and information sharing) between the US-responsive neurons and the CS+ responsive neurons, and (4) that these changes specifically occurred in the neural population that encodes the CR after the CS+-US pairing, and not in the outside network (Non-CR ensemble). This observation is consistent with the observation in the basolateral amygdala, which indicated that entire recorded neurons in the amygdala revealed the assimilation of the CS+ and US as a whole population, and that it was significantly correlated with the enhanced behavioral responses^44^, which supports the reliability of our results. Also, the same study^44^ reported the enhancement of the CS+ responsiveness in the basolateral amygdala during the repeated CS-US pairing for the conditioning. We found that the CS+ responsiveness in the dmPFC during the late fear conditioning phase significantly affected the integration possibility of these responsive neurons into the CR ensemble (Supplementary Fig. 11). This result suggests the possible dynamic interaction between dmPFC and amygdala during the development of the associative memory network.

More than 60 years ago, Hebb proposed that repeated co-activation of a group of neurons might create a memory trace through the enhancement of connections^45^. Previous studies based on the artificial manipulation of neuronal activity suggested the in vivo contribution of the enhanced neural activity to form the associative memory network, and studies based on molecular markers such as CREB or immediate early genes provide strong support that the activity-dependent mechanism underlies endogenous associative learning^3,46–48^. Our results, based on longitudinal live imaging and model-based analyses, not only support these findings but also allow for detailed visualization of the neural network dynamics. We observed that the US-responsive neurons (Fig. 4f–k), perhaps with CS+ responsive neurons (Supplementary Figs. 9, 11, 12), were dominantly integrated into the specific neural population encoding the CR, suggesting that Hebbian plasticity (i.e., fire together, wire together) or some other co-activation based mechanism underlies the reorganization of the prefrontal network functional connectivity during associative learning and enable the emergence of a specific link between the US-responsive neurons and the CS+ responsive neurons to form a novel CR network in the dmPFC.

The dmPFC is defined differently among previous studies. Using our two-photon microscopy imaging system through a microprism, we precisely adjusted the anatomical coordinates of the field of view for the imaging. We recorded neural population activities precisely from the dorsal part of the medial prefrontal cortex (Supplementary Fig. 14) using the position of the dorsal surface of the brain, sinus, and pial surface along the midline, which were usually visible through the imaging window, as a guide. We specifically targeted the surface layer of the dmPFC (~150–200 μm from the pial surface along the midline). This precision and the detailed coordinate information will be crucial for more systematic comparison of the present study with previous and future related works.

Graphical modeling (CRF modeling) enabled visualization of the functional dependence between individual neurons of these selected neurons based on a mathematical model accounting for the contribution to the population coding, indicating the possible signal flow in the dmPFC underlying associative fear memory as discussed above. A previous study that introduced a similar type of mathematical analysis evaluating neuronal co-activation also reported the increased functional coupling of the US-responsive neurons and CS-responsive neurons in the ventral hippocampus as a result of contextual fear learning^49^. Together with this study for contextual fear learning and another previous study in visual discrimination learning^42^, our results strengthened the usefulness of this type of functional connectivity analysis based on neural coactivity during learning and memory retrieval.

We observed that individual dmPFC neurons responded to multiple task-relevant aspects (Supplementary Fig. 5), which has also been reported in the primate PFC^30^ and referred to as “mixed selectivity”. A similar feature was observed in the mouse caudal PFC during a decision-making task^50^, suggesting that this feature is not specifically observed in our behavioral paradigm. As a potential advantage, mixed selectivity is proposed to enhance the number of tasks that a limited number of neurons can handle through high-dimensional neural representations implemented by a population of neurons^30,31^. A further detailed investigation is important to understand how the dmPFC neural population encodes multiple memories and tasks.

We observed that the CR information was redundantly encoded in the dmPFC at the level of the population coding. Our analysis based on the elastic net allowed us to select neurons encoding a specific locomotion state as a population of neurons. By the adjustable parameter named alpha, we could not only optimize the number of the selected neurons but also search for possible overlap in information coding. Note that this method analyzed the information coding not as individual neurons 1-by-1 but as a population. Interestingly, at some alpha value, even without (i.e., after removing) the minimum number of neurons required to estimate the CR information at ~100% accuracy, the remaining neurons still possessed the CR information at high accuracy, similar to the pre-removed neuron set (in Supplementary Fig. 6b, at the right bottom panel, zero (dark blue) for some alpha in the mouse #5, for example, means no difference between pre-removed and post-removed). We needed to adjust the alpha and the size of the selected neurons to maximally extract the neural population encoding the CR information, depending on the mouse. This observation itself suggested that the CR information was redundantly encoded by multiple neural populations in the dmPFC (i.e., both minimally-extracted population A and remaining population B encoded the CR information). In terms of the population coding, this type of tendency was not observed when we extracted the regular motor-coding population, named the RS ensemble (Supplementary Fig. 7), suggesting that population coding of the CR information in the dmPFC may be unique and based on the redundant coding. The observation at each single-cell level has demonstrated that many neurons show similar response types to tones or foot shocks (Fig. 4, and Supplementary Figs. 5, 9), but our study also revealed that they were functionally connected to each other, and seemed to work together in the CR ensemble during the memory retrieval phase. It would be interesting to further investigate how and why these individual neurons contribute to the redundant (or separate) groups of neurons.

The advantage of the redundancy is not clear, but because fear memory is critical for animal survival, it is possible that the redundant coding for fear memory is not inefficient but rather evolutionarily crucial. On the other hand, although the redundancy can also be considered inefficient in terms of the short-term cost, because the dmPFC is known to be involved in long-term memory via brain-wide networks^12,18,21^, it would be interesting to investigate whether the redundantly encoded information for the CR is maintained or diminishes by longer-term continuous recording, and whether it is related to the brain-wide regulation of memory using virus-based anterograde or retrograde fluorescent labeling techniques to simultaneously dissect the downstream or upstream structures.

As we have successfully discriminated the specific neural population encoding the CR as well as the detailed internal structure with a hub of the US-responsive neurons, further testing the causality of the identified structure to the locomotion state by holographic optogenetics^41^ could be intriguing. Importantly, however, we also found that the dmPFC responds to auditory signals even prior to the associative learning (Supplementary Fig. 5) and that the CR ensemble predominantly includes the US-responsive neurons (Fig. 4h). Therefore, to validate the causality as a memory network, the stimulation experiment needs to be carefully designed because enhancing the sensory coding can also modify behavioral responses in a task based on the sensory stimuli as demonstrated before^41^, and because activating US-responsive neurons may sufficiently encourage innate defensive freezing responses as unconditioned responses. Further mathematical dissection and additional anatomical dissection as discussed in the preceding paragraph would be the next important step to more precisely identify the memory-specific connections and information flow to be tested by holographic optogenetics.

## Methods

### Animals

All animal experiments were carried out in accordance with the Institutional Guidance on Animal Experimentation and with permission from the Animal Experiment Committee of Osaka University (authorization number: 3348), or in accordance with National Institutes of Health guidelines and approved by the National Institute for Physiological Sciences Animal Care and Use Committee (approval number 18A102). Male C57BL/6 mice housed under a 12-h light/dark cycle with free access to food and water in a temperature-controlled environment (22–24 °C and 30–60% humidity) were used for all experiments. Behavioral experiments were performed during the dark cycle (i.e., when mice were normally awake) using singly-housed mice. Mice at 4–6 months of age were used for the behavioral and imaging experiments.

### Virus injection

To express GCaMP6f, a genetically encoded calcium indicator to monitor neural activity, we used a gene expression system based on the AAV vector. Viruses were injected into mice at postnatal day (P) 50–120 for in vivo experiments, at least 1 month before the microprism implantation, which was followed by the in vivo experiments 1–3 months after the implantation. Injection procedures were performed as described previously^33^, with some modifications. During surgery, the mice were anesthetized with isoflurane (initially 2% [partial pressure in air] and then reduced to 1%). A small circle (~1 mm in diameter) of the skull was thinned over the left medial prefrontal cortex (mPFC) using a dental drill to mark the site for a small craniotomy. AAV1/CamKII.GCaMP6f was obtained from the University of Pennsylvania Vector Core, and injected into the left mPFC (slightly away from the imaging target area to avoid damaging the field of view) at 3 sites (depth 1.0, 1.5, and 2.0 mm from the dorsal surface of the brain, volume 375 nl/site) to cover the dorsal mPFC, over a 5-min period at each depth using a UMP3 microsyringe pump (World Precision Instruments). The X-Y coordinates for the injection site were usually 0.5 mm lateral to the midline and 2.0 mm rostral to bregma, but if large blood vessels obstructed the position, we shifted the insertion site slightly to avoid the vessels. The beveled side of the injection needle was faced to the midline so that the needle could be smoothly inserted and the virus would cover the superficial layers of the mPFC. We carefully designed our injection protocol (especially the volume and depth) to widely cover the mPFC areas, which include, according to the nomenclature in the Allen Brain Atlas (https://atlas.brain-map.org/), the ACA (anterior cingulate area), PL (prelimbic area), and dorsal part of the IL (infralimbic area). The anatomical coordinates of the field of view for the two-photon imaging were precisely targeted to record neural population activities from the dorsal part of the mPFC (i.e., dmPFC) using the position of the dorsal surface of the brain, the sinus, and the pial surface along the midline, which were usually visible through the imaging window prepared as described below, as a guide. We recorded neural activities through the implanted microprism in the dmPFC that specifically included the PL and part of the ACA, and little or none of the IL, specifically from the superficial layer (~150–200 μm from the pial surface along the midline) of the dmPFC (see Supplementary Fig. 14 for details; the field of view ranged from a depth of ~0.4–1.8 mm and centered at a depth of ~0.8–1.4 mm from the peak point of the dorsal surface of the brain above the dmPFC).

To perform chemogenetic silencing of the dmPFC by DREADD, we expressed the inhibitory human M4 muscarinic cholinergic Gi-coupled DREADD (hM4Di) fused to mCherry bilaterally in the dmPFC based on the AAV vector (AAVdj/CaMKIIα-hM4Di-mCherry). For the non-silenced control, we used AAVdj/CaMKIIα-mCherry. The pAAV-CaMKIIα-hM4Di-mCherry plasmid was constructed by excising the hM4Di-mCherry sequence from the pAAV-hSyn-DIO-hM4Di-mCherry plasmid^51^ and inserting it into the pAAV-CaMKIIα-DIO-hM3Dq-mCherry plasmid^51^ using SalI and EcoRV sites and standard cloning techniques. The pAAV-CaMKIIα-mCherry plasmid was previously constructed by Niu et al.^51^. Using these plasmids, the AAVdj/CaMKIIα-hM4Di-mCherry and AAVdj/CaMKIIα-mCherry were produced and purified respectively, following the methods described previously^52,53^. Viruses were injected bilaterally into the dmPFC at postnatal day (P) 60–90, which was followed by the behavioral experiments ~1 month after the injection. The beveled side of the needle was faced rostrally so that viruses could be injected into the anterior dmPFC where we observed the neural activities in the present study. We targeted 0.4 mm lateral to the midline in both hemispheres, ~2.0–2.1 mm rostral to bregma (we adjusted the position to avoid hitting the bold blood vesicles on the surface of the brain), and a depth of 1.5 mm from the pial surface with 450 nl/site. After the behavioral experiments as described in the “Silencing dmPFC excitatory neurons by DREADD” section, the brain of the mouse was removed following perfusion with phosphate-buffered saline (PBS, pH 7.4) and 4% paraformaldehyde (in PBS) under isoflurane anesthesia (2%), fixed in 4% paraformaldehyde at 4 °C overnight and sliced into 200-μm coronal sections. Slices were mounted in Vectashield with DAPI (Vector Labs, H1500), and fluorescence images were obtained by an Olympus BX63 or a ZEISS LSM 980. Only mice in which bilateral expression in the dmPFC was confirmed were used to evaluate the effect of dmPFC silencing.

### In vivo two-photon imaging

In vivo two-photon imaging was performed as described previously^26,33^, with modifications to pair with our new experimental system. At 1–3 months after the virus injection, the mice were anesthetized with isoflurane (initially 2% [partial pressure in air] and reduced to 1%). A titanium head plate described in a previous paper by Goldy et al.^54^ was selected for the present study to minimize the area lying over the ear and to minimize the blockage of auditory input through the ear. The head plate was attached to the skull with dental cement. For the subsequent microprism implantation, a square cranial window (~2.3 × 2.3 mm) was carefully made with minimal bleeding above the right mPFC, the hemisphere opposite to the virus injection site. An implantable microprism assembly^26^, comprising a 2-mm right angle glass microprism (TS N-BK7, 2 mm AL+MgF2, Edmund) bonded to a 2 × 2 mm square cover glass (No.1; Matsunami) for the middle position and a 4 × 4 or 3 × 4 mm glass window at the surface position of the imaging window, was prepared and inserted into the subdural space within the fissure along the midline as described previously^26^ to avoid harming any nerves surrounding the mPFC network in both hemispheres, thereby allowing for visualization of the left mPFC, which was previously injected with the GCaMP6f virus, through the imaging window. The area directly beneath the microprism was compressed but remained intact. This insertion procedure sometimes caused a small amount of bleeding that covered the imaging site, but even in that case, the imaging window became clear after waiting at least a month before performing the experiments. As reported before^26^, the mice recovered quickly and displayed no gross impairments or behavioral differences compared with non-implanted mice, enabling chronic imaging of the dmPFC in behaving mice.

The activities of dorsal mPFC neurons were recorded by imaging fluorescence changes with a FVMPE-RS two-photon microscope (Olympus) and a Mai Tai DeepSee Ti:sapphire laser (Spectra-Physics) at 920 nm, through a 4× dry objective, 0.28 N.A. (Olympus) or a 16× water immersion objective, 0.80 N.A. (Nikon). Mean ( ± SE) frame rate was 8.96 ± 0.87 (frames/sec). GCaMP6f signals were detected via the band-pass emission filter (495–540 nm). As the GCaMP6f was expressed under the regulation of the CaMKII promoter^28,29^, all of the recording targets were assumed to be excitatory neurons^27^. Scanning and image acquisition were controlled by FV30S-SW image acquisition and processing software (Olympus). To smoothly set the mice below the objective lens for the imaging, light and minimal-duration isoflurane (2.0% for <2–3 min) anesthesia was used, and behavioral and imaging experiments were started 5 min after the mice awoke and began locomoting on the running disk, which was visually confirmed via the video camera (VLG-02, Baumer) under infrared light-emitting diode illumination (850 nm: LDL-130X15IR2-850, CCS Inc.). To detect neural activity from the same set of neurons in each mouse over multiple days, the depth from the surface of the brain (dmPFC area) and configuration of blood vessels and basal GCaMP6f signals in each field of view were recorded and referenced as described previously^55^.

### Fear conditioning, memory retrieval, and extinction under the microscope

The experiments were designed according to previous studies^13,15,23^, with some modifications to optimize conditions for the two-photon microscope system. Details are described in Supplementary Fig. 1. In the present study, we define the term “state” to describe whether a mouse was locomoting, spontaneously stationary, or expressing a CS+-induced freezing-like response. We followed the fear conditioning protocol applied in previous studies in freely moving mice^13,15,23^. For example, we used the same number and types of tones and the same intervals at each step, except that we used 7 CS+-US pairings rather than the 5 or 6 pairings in the previous studies and we used a milder foot shock current (as explained below). In the present study, the term “session” is defined as a series of CS presentations with or without a paired US on each day (e.g., habituation session, fear conditioning session, and a post-FC session on the day after the fear conditioning session), while the term “trial” indicates each 30-sec CS presentation (with or without US) (Supplementary Fig. 1c). We also use the term “phase” in the present study to distinguish the early and late trials during the same (consecutive) session. The heads of the mice were fixed under the objective lens for two-photon imaging, allowing them to run freely on the running disk placed below them, and locomotion was measured by the rotation of the running disk, as previously described^56^. Experiments were performed in a completely dark environment to protect the detector (photo multiplier tube) for the two-photon imaging from the room light. We prepared 2 different types of running disks to establish 2 different contexts as used in conventional fear conditioning experiments for head-unfixed mice^13,14,23^. Disk A was made of light-colored plastic with ridges from the center to the rim that the mice could grip to allow them to easily rotate (and walk on) the disk^56^. Disk A was used for adaptation (Day [D] 1 and D2) and for memory retrieval and extinction (D4). Disk B was built for the fear conditioning (D3), and comprised a grid made of stainless steel bars (Fig. 1a), which was attached to a foot shock generator (SGA-2010, O’HARA & CO., LTD) via an electrical slip ring so that electrical current to this running disk for the foot shock (US) could be stably delivered to the mouse irrespective of whether the running disk was rotating. The behavioral sessions on each day began only after the mouse was constantly locomoting for more than 5 min. The running disks and the surrounding area (inside the cage for the microscope) were cleaned with 70% ethanol before and after each experiment. To score regular stationary state or freezing-like responses as a CR, the speed of the mouse locomotion was measured by the rotation speed of the running disk^56^. Mice were considered to be stationary (during the no-CS period) or expressing a freezing-like response (during CS+/memory retrieval) if no movement was detected for at least 1 sec. On D1 and D2, the mice underwent an adaptation session with disk A for an hour each day, to familiarize them with the novel environment. On D3, the mice underwent a habituation session in context B, in which they received 4 presentations of the CS− and CS+ alternately (total CS duration, 30 sec for each trial; consisting of 50-ms pips at 1 Hz repeated 30 times; pip frequency, 7.5 kHz or white noise, respectively, 80-dB sound pressure level (60-dB basal room noise produced by the heating, ventilation, air conditioning system, and 20-dB for the CS). The habituation session was immediately followed by discriminative fear conditioning^13,14,23^ on the same day by pairing the CS+ with a US (1-sec foot shock, 7 CS+-US pairings). Compared with the protocol used in the previous studies^14,15,23^, a slightly higher number of repeated CS+-US pairings was used (5 CS+-US pairings in the previous studies), while the intensity of the foot shock current was much milder (0.6 mA or 0.5 mA in the previous studies). The intensity of the foot shock was usually 0.03–0.07 mA in the present study, but when mice failed to respond to the US, likely due to the running disk becoming dirty or wet from the mice and thus possibly suppressing or shunting the flow of current during the experiment, an intensity of 0.10–0.45 mA was used. Of the total 23 mice that we used for the behavioral experiments without chemogenetic manipulation of brain activity, seven mice required a higher shock intensity; 0.10, 0.15, 0.25, 0.35, 0.35, 0.35, or 0.45 mA. Note that, as explained in the next paragraph and Supplementary Fig. 2, the intensity adjustment did not enhance the conditioned response (CR, i.e., enhancement of the freezing-like response or decrease of the locomotion) but instead resulted in the equivalent responses. Also, 2 out of 5 mice injected with AAVdj/CaMKIIα-hM4Di-mCherry and 1 out of 5 mice with AAVdj/CaMKIIα-mCherry required a higher shock intensity (0.15, 0.15 for AAVdj/CaMKIIα-hM4Di-mCherry, and 0.15 for AAVdj/CaMKIIα-mCherry, respectively). The response to the foot shock (and whether or not the intensity was sufficient) was manually evaluated on the basis of the detection of enhanced locomotion and/or vocalizations emitted by the mice immediately after foot shock onset (example recording of these responses to the foot shock are shown in Supplementary Fig. 1d). We confirmed that at least 5 of a total of 7 CS+-US pairings induced an unconditioned response. The onset of the US coincided with the onset of the last sound pip of each 30-sec CS trial. The CS− and the CS+ trials were performed alternately (inter-trial intervals, 50–150 sec). On D4, conditioned mice underwent a post-FC session on disk A, which included an early memory retrieval phase and a following late extinction phase as described in the Results section (they received a total of 4 presentations of the CS− and 12 presentations of the CS+ as shown in Supplementary Fig. 1). During the experiment (D1–4), the mouse was continuously encouraged to locomote by administering a 4-ul drop of 0.1–0.3% saccharin water per 100 cm of locomoting, provided through a spout placed near their mouth^55^ so that the freezing-like response could be discriminately detected as decreased locomotion (Fig. 1). The mice were not water-deprived.

To clarify how we needed to adjust the intensity depending on the experiment, we measured the current on the wet disk without mice. We observed a clear tendency for moisture to reduce the amount of current. To further complicate matters, we observed that the amount of current did not become weaker as a function of the wetness but rather became zero, while the current above a certain level gradually began to flow (i.e., it did not suddenly become stronger). Although it was difficult to monitor the actual current flow through the mouse during the actual experiments, according to the observation above, we assumed that gradually increased currents provided a similar shock level to each mouse. Actually, when we compared the behavior of the mouse group that experienced a stronger foot shock (0.10–0.45 mA) and the group experiencing a weaker shock (0.03–0.07 mA), we observed no significant difference in the freezing-like response and locomotion level during the CS+ in the first four trials of the D4 post-FC session, supporting that adjusting the current level to produce a similar behavioral response to the US resulted in similarly aversive experiences to the mice for the fear conditioning (Supplementary Fig. 2d, e).

The locomotion speed and timings of the tones and the foot shock were synchronously recorded with image acquisition (GCaMP6f imaging in dmPFC) using NI software (LabVIEW 2015/2018; National Instruments) and NI-DAQ (National Instruments). The results shown in Fig. 1 and Supplementary Fig. 2 indicate that this protocol led to the mice successfully learning the CS+-US association, and that locomotion was reduced in response to the CS+, but not the CS−, only after the fear conditioning session, enabling us to observe changes in neural representations in the dmPFC occurring as a result of the fear conditioning.

### Electrocardiogram recording and heart rate analysis

To measure the heart rate during the CR or fear memory retrieval (on day 4), an electrocardiogram (ECG) was recorded from the awake mice using a previously described method for anesthetized mice^57^ with some modifications. A single pair of electrodes was anchored subcutaneously as illustrated in Supplementary Fig. 3a; The negative electrode (−) was placed in the mouse’s right upper chest, and the positive electrode (+) was placed in the left abdomen. ECG signals were referenced to the ground (left upper chest). The ECG was amplified with a gain of 1000 and filtered between 10 and 1000 Hz together with a notch filter (60 Hz), all of them using a differential AC amplifier (model 1700; A-M Systems, Inc.). The cables were bound above the mouse, and the cable bundle was clipped to a flexible magnetic stand base holder and then connected to the amplifier (Supplementary Fig. 3b).

The ECG signal was synchronously recorded using LabVIEW 2015/2018 and NI-DAQ together with locomotion and other stimulus timings at a 1000-Hz sampling rate. We analyzed the records using MATLAB R2014a and R2019b (MathWorks, Natick, MA). Our head-fixed system allowed us to stably record the ECG in awake mice, and mild motion artifacts could be eliminated by subtracting the baseline level calculated by the moving average. The typical R-wave peak of each heartbeat was clearly identified (Supplementary Fig. 3c), and the detected peaks were used to calculate the heart rate (beats per minute [bpm]) for each 100-ms bin to evaluate the dynamic change accompanied by tone presentations and behavioral change (Supplementary Fig. 3d–f).

In the present study, it was important to examine (1) whether the freezing-like response observed in our head-fixed system is physiologically similar to freezing in freely moving mice and different from the regular stationary state (without CS+) and (2) whether it is dependent on the dmPFC activity or not. We simultaneously performed the ECG recording to monitor the heart rate and the DREADD-based manipulation of the dmPFC activity together with non-silenced control mice. We describe how we performed their statistical evaluation below.

We first analyzed the difference in the heart rate between the freezing-like response (under the CS+ presentation) and the regular stationary state (without CS+) to evaluate the nature of the freezing-like response in our system using the control mice. 4 out of 5 control mice (non-silenced and CNO-injected mice) showed heart rate deceleration during the CS+-evoked freezing-like response (Supplementary Fig. 4a, b). For the statistical comparison, we first used a paired permutation test to compare the heart rates between the freezing-like response (under the CS+ presentation) and the regular stationary state (without CS+) since it requires no assumptions regarding the data distribution calculating a statistic (difference of means) of paired permuted data. The *p* value calculated from the distribution of the statistic obtained by permutation was “<0.0625” for this comparison (Supplementary Fig. 4b), which is the minimum value for the comparison of four pairs in the paired permutation test. We further performed tests based on bootstrap resampling to more systematically evaluate the difference between the two groups (the heart rate during the freezing-like response vs that during the regular stationary state). For this purpose, we first calculated the bootstrap means for each individual, which was further used to calculate the medians for each group. This was repeated in total 2000 times (Supplementary Fig. 4c) (we also describe details for the bootstrap resampling in the section “Imaging data analyses and statistics”), and eventually obtained 2000 ratio values (heart rate during the freezing-like response was divided by that during the regular stationary state; Supplementary Fig. 4d). The *p* value was calculated from the distribution (Supplementary Fig. 4d). This analysis certified that the heart rate during this freezing-like response under the CS+ presentation was significantly slower than that during the regular stationary state. We also statistically confirmed that the heart rates during the freezing-like response and those during locomotion under the CS+ presentation on D4 (the first 4 trials during the post-FC session) were significantly different (Supplementary Fig. 4k, right), suggesting the physiological difference between these two states during the CS+ presentation. This observation is also important for the present study since we further utilized this state difference to extract the neuronal population encoding the information for the CS+-evoked freezing-like response. The heart rates without the CS presentation were also similarly enhanced during locomotion; those during the locomotive state were significantly faster than those during the non-locomotive state (Supplementary Fig. 4k, left).

Similarly, we analyzed the heart rate of the dmPFC-silenced mice (Supplementary Fig. 4e). The paired permutation test indicated that the heart rates between the non-locomotive state (under the CS+ presentation) and the regular stationary state (without CS+) were not significantly different in the dmPFC-silenced mice (Supplementary Fig. 4f). The analyses based on the bootstrap resampling also revealed no significant difference in the dmPFC-silenced mice (Supplementary Fig. 4g, h).

As for the analyses based on the bootstrap resampling in the present study, calculating the median of multiple mice to evaluate each group (not the means used for other bootstrap resampling-based analyses in the present study) can be advantageous when outliers are included in a small-size group (Supplementary Fig. 4b, f). But we need to be careful that it might also lead to the wrong conclusion by ignoring some of the samples. Therefore, we also tested the statistical comparison based on the median for the group calculated by additional bootstrap resampling-based on the mean value for each individual obtained by bootstrap resampling (here we first calculated the bootstrap mean for each individual as described above, and additionally we calculated bootstrap median for each group using such individual value). By verifying these results, we obtained similar results confirming that the heart rate during the freezing-like response in the control group was significantly slower compared to the regular stationary state (*p* = 0.019) and that the dmPFC-silenced mice showed no significant change (*p* = 0.614).

We also directly compared the dmPFC-silenced mice (AAVdj/CaMKIIα-hM4Di-mCherry) and non-silenced control mice (AAVdj/CaMKIIα-mCherry). For this purpose, we first scored the differences between the impact of the CS+ on the heart rate and that of the CS− in each mouse (each was calculated by dividing the 29-sec during-trial heart rate with the 29-sec pre-trial heart rate based on the first 4 trials of either the CS+ or the CS− on D4). For the statistical comparison in each mouse, this value was not directly calculated from the original data but through the bootstrap resampling. Since mice behaviors are not consistent over multiple trials, all-time-point data (1-sec bin in the case of the heart rate data, i.e., 29 × 4 samples in total) were pooled to perform the resampling, calculate the bootstrap means, and further calculate the differences between the impact of the CS+ on the heart rate and that of the CS− in each mouse for each resampling round. After repeating this resampling 2000 times, we statistically evaluated whether the distribution of the difference values (2000 samples for the CS+ vs CS−) are statistically different from zero (i.e., whether the impact of the CS+ on the heart rate and that of the CS− in each mouse are significantly different). 4 of 5 control mice (AAVdj/CaMKIIα-mCherry) showed significant decreases (Supplementary Fig. 4i). On the other hand, 1 of 5 control mice (AAVdj/CaMKIIα-mCherry) exceptionally showed enhanced locomotion (flight-like response) that was accompanied by an enhanced heart rate even when the mouse was not locomoting under the CS+. This type of flight-like response was very exceptional in the case of the naive mice (non-CNO injected), and only 1 of 23 naive mice showed certain, but milder, enhancement of locomotion. We included this mouse (one control mouse showing flight-like responses) for fair statistics in direct comparisons of dmPFC-silenced and non-silenced control mice, and observed that the enhancement of the heart rate of the mouse was also significant (Supplementary Fig. 4i). Because the previous study showed the dmPFC also contributes to a flight-like defensive behavior^16^, we expected that not only the freezing-like response but also the flight-like response could be suppressed by silencing the dmPFC. We further observed that 4 of 5 dmPFC-silenced mice showed no significant difference between CS+-evoked change and CS−-evoked change and none of them showed significant enhancement of the heart rate by the CS+ (Supplementary Fig. 4i). Fisher’s exact test indicated that a statistically significant association existed between the dmPFC activity and CS+-evoked change in heart rate, supporting that the dmPFC normally contributes to memory retrieval (Supplementary Fig. 4j). Note that, the mouse of flight-like responses was excluded when evaluating the physiological nature of the freezing-like response in the control mice (since it was a rather flight-like exceptional response). Also, this type of exceptional mouse was not included in the imaging data analyses to extract the CR ensemble.

### Silencing dmPFC excitatory neurons by DREADD

To test the contribution of the dmPFC to memory retrieval after the fear conditioning in our head-fixed system, we performed chemogenetic silencing of the dmPFC using the DREADD platform. We injected the AAVdj/CaMKIIα-hM4Di-mCherry bilaterally in the dmPFC to silence the activities of the excitatory neurons, or AAVdj/CaMKIIα-mCherry as a non-silenced control, as described in the section “Virus injection”. The mice were given an intraperitoneal injection of clozapine-N-oxide (CNO; 5 mg/kg body weight) 30 min before the first trial of the D4 post-FC session. To evaluate whether the CR observed in our head-fixed system is physiologically similar to freezing, we simultaneously monitored heart rate during the experiments as described under the subheading “Electrocardiogram recording and heart rate analysis” because freezing is reportedly accompanied by heart rate deceleration in freely locomoting mice^24^ as well as in other species^25^. Only mice in which bilateral expression in dmPFC was confirmed (as described under the subheading “Virus injection”) were used to evaluate the effect of the dmPFC silencing.

### Vocal recording and visualization

Vocalization of the mouse during the fear conditioning was recorded using an ultrasonic microphone (CM16/CMPA, Avisoft) amplified (UltraSoundGate 116H, Avisoft) and digitized at 250 kHz (by a software, Avisoft-RECORDER USGH, Avisoft). For the visualization of the vocalization, we generated a spectrogram using a multitaper method^58^. In this analysis, multiple time tapers were designed using a set of 6 series of discrete prolate spheroidal sequences with the time half bandwidth parameter set to 3^59^. The length of these tapers was set to 512 data samples (~2 ms at 250 kHz). The stored waveform was multiplied by each taper and transformed into the frequency domain. These multiplied waveforms were averaged in the frequency domain. This procedure could produce a stable spectrotemporal representation of vocal sound with background noise attenuated.

### Imaging data analyses and statistics

The raw images of the GcaMP6f signals in the dmPFC were processed to correct for brain motion artifacts using the enhanced correlation coefficient image alignment algorithm^60^. To apply the same regions of interest (ROIs) for analyzing the images obtained across multiple days, the movies from the same mouse were precisely aligned with each other using the same enhanced correlation coefficient algorithm as above, while, for a local shift (shift of a few pixels in a small number of neurons among all recorded cells), the corresponding ROIs were manually adjusted.

The ROIs for the detection of neural activity were automatically selected using a constrained nonnegative matrix factorization algorithm in MATLAB R2014a/R2019b as described previously^61^, with some manual adjustment. Further steps to process the GcaMP6f signals for measurements of the signal change (ΔF/F) of each neuron were performed as described previously^33,62^; although the same constrained nonnegative matrix factorization package for ROI detection also provides an option for signal processing that was not sufficiently optimized to analyze our data, which were obtained over several days with more than 30,000 frames each day. Fluctuations in the background fluorescence, which contains synchronous fractions across nearby neurons^61,62^, was subtracted before calculating the ΔF/F of GcaMP6f signals as described previously^33^. Briefly, a ring-shaped “background ROI” was created for each ROI 2–5 pixels away from the border of each neuronal ROI to a width of 30–35 pixels, and the size was adjusted to contain at least 20 pixels in each background ROI after completing the following steps. From the background ROI, we removed the pixels that belonged to any neuronal ROIs, and the ROIs that contained artificially added pixels (black pixels added at the edge of the image due to the motion correction procedure) at any time point. We then removed the pixels that, at some time point(s), showed signals exceeding that of the neuronal ROI by 2 standard deviations of the difference between each background ROI pixel time series and the neuronal ROI time series. The resulting background ROI signals were averaged at each time point, and a moving average of the time series was calculated. Using the moving average instead of the raw background ROI signal was helpful to minimize the production of an artificially large increase or decrease at each time point due to the subtraction, which could have altered the analyses of the timing of neural activations. Pixels within each neuronal ROI were also averaged to give a single time course, and then the background ROI signal was subtracted. Then, the ΔF/F of GcaMP6f signals of all recorded neurons was calculated. For most of the analyses and comparisons of the results from multiple mice, the ΔF/F data were further z-normalized within each experiment (same mouse, same day) as described previously^13,23^, and as explained below (for more details). On the other hand, particularly for the CRF modeling used to evaluate the functional network connectivity, the spike probabilities were inferred from the ΔF/F as an alternative estimate of neuronal activation using a constrained sparse nonnegative calcium deconvolution method^61^. We used the code “constrained_foopsi.m”^61^, and the parameters used in the calculation were not manually selected but estimated from the data by the code. After inference of the spike probability and further thresholding by 2 standard deviations, the obtained binominal data were further binned (bin size: 1 sec). Importantly, the results obtained by CRF modeling were consistent with the results of the coactivity analyses based on the ΔF/F (and z-normalized ΔF/F) (Supplementary Fig. 10), providing substantial support that the analyses based on both estimates complemented each other for the data analyzed in the present study. While neurons for the analyses were initially automatically detected, neurons responding to noisy signals with no apparent calcium transient at any time during the experimental days were identified by visual inspection and excluded from further analysis.

For the statistical analysis, we used MATLAB R2014a/R2019b. The Wilcoxon signed-rank tests for paired comparisons or the Wilcoxon rank-sum test (equivalent to Mann-Whitney U test) for unpaired comparisons was used to determine statistical significance (*p* < 0.05) unless otherwise indicated. Two-tailed tests were selected for all statistical analyses. All p-values less than 0.0001 are described as “*p* < 0.0001” (or ****). Graphs were produced by MATLAB R2014a/R2019b or Excel (Microsoft). When comparing two groups (e.g., CR ensemble vs Non-CR ensemble) consisting of the results of multiple mice, in addition to the analyses using original data (e.g., *N* = 7 [D3] vs *N* = 7 [D4]) (Figs. 3d, h, 4b, c, and Supplementary Figs. 8, 10b), we performed tests based on resampling (Fig. 4e and Supplementary Figs. 4c, d, g, h, k, and 10c) to more systematically estimate representative values (e.g., mean or mean difference) of each group where the number of recorded neurons in each mouse (field of view) varied. We resampled the same sample size from each data (each mouse, each day) with replacement (i.e., collected a bootstrap sample). For example, if the original data of a mouse contained data of 100 neurons, the size of the resampled data for the mouse was also 100 neurons (but with accepting overlapped sampling). For 100 pairs (e.g., correlation coefficients between neurons), 100 pairs were resampled. During this resampling, we sampled the data from the same set of neurons for each day (for example, if resampled data for D3 contained the 4 data from neuronal pairs A-B, A-B, C-D, E-F, the resampled data for D4 also contained data from pairs A-B, A-B, C-D, E-F), so that we could further calculated differences between D3 and D4 of each original pairs (e.g., “Change in each score (D4E minus D3E)” in Supplementary Fig. 10 could be calculated from these resampled differences; with this way, we could also calculate D3L minus D3E, etc.). After we calculated the bootstrap mean for each mouse (at each category [e.g., CRE, NoCRE, etc.]), we obtained a bootstrap mean of the differences (e.g., D4E minus D3E) across all mice (*N* = 7) at each category. To consider the difference between two different categories (e.g., CR ensemble vs Non-CR ensemble), we further calculated the difference of two bootstrap means. These bootstrap means were collected with 2000 independent bootstrap samples. The *p* value was calculated from the distribution of bootstrap means (examples for the calculation are shown in Supplementary Fig. 4d and h). On the other hand, when statistically comparing original data (Figs. 3d, h, 4b, c, and Supplementary Figs. 8, 10b), we used a paired permutation test that requires no assumptions regarding the data distribution, calculating a statistic (difference of means) of paired permuted data. The *p* value was calculated from the distribution of the statistic obtained by permutation. The p-values obtained by this method and the evaluated statistical significance were very similar to those obtained by the paired t-test in almost all cases. When plotting the entire large number of samples, we used a MATLAB code, CategoricalScatterplot (https://github.com/AbstractGeek/CategoricalScatterplot).

In the present study, to compare changes in neural responses and ensemble representations occurring as a result of the fear conditioning, without any bias, we did not exclude neurons that showed no response to the CS on D4 from the analyses, which was done in some previous experiments^23^. Neurons for the analyses were automatically selected based on the neural responses, as described above, and all neurons that exhibited clear activity during at least 1 of the experimental days were included for the analyses irrespective of whether it was during the CS presentation or only during no CS presentation, considering the previous work suggesting that not only the neurons that typically respond to the CS, but also other types of neurons (including those of mixed selectivity) are important for population coding in the prefrontal network^30^.

The significance of CS-induced neural responses was determined as reported in previous studies^13,23^. Signals during CS presentation were normalized to baseline activity using a z-score transformation, as described previously^13,23^. The CS-induced neural activity for each stimulus was then calculated as the mean of the activity during ~1 sec from each stimulus onset (depending on the imaging frame rates, we set the number of frames to be used for this calculation so that sampling duration was closer to 1 sec but the frames that overlapped with the next stimulus onset was excluded). The last sound pip of each 30-sec CS trial was also excluded from this analysis because, during fear conditioning, the last sound pip of the CS+ overlapped with the US (we excluded the last pip data not only for analysis of CS+-evoked responses during fear conditioning but for all data analyses on both D3 and D4, for both CS+ and CS−). They were averaged over blocks of 3 CS trials consisting of 87 individual sound pips in total, for D3-early (first 3 trials during the fear conditioning session), D3-late (last 3 trials during fear conditioning on D3), D4-early (first 3 trials on D4, as responses during memory retrieval), and D4-late (last 3 trials only for CS+ on D4 as responses during extinction), respectively, or used to statistically test whether the responses of each neuron were significantly different from zero (baseline) and to define CS-activated / -inactivated neurons.

One concern was that the off kinetics of the GcaMP6f signal might not be sufficiently fast to consider the responses to 87 individual sound pips as independent data, and thus statistical evaluation based on these responses might not be reliable. As shown in Supplementary Fig. 5c, however, neurons categorized as producing a significant response tended to exhibit substantially higher mean responses than those that produced non-significant responses, providing support that this criterion was helpful for determining the characteristics of the individual neurons. Also, because of the limited number of trials at each phase (3 trials for each [D3-early/D3-late/D4-early/D4-late]), statistics based on the trial-by-trial comparison could not be performed to evaluate the CS-induced neural responses.

The US number was limited (a total of 7 stimuli per mouse); therefore, to define US-responsive neurons, the mean neural activity (z-normalized as described above) of each neuron for 1.5 sec from US onset was calculated, and US-responsive neurons were defined as neurons with 1 or higher mean neural activity. The number of the selected US-responsive neurons was very limited (zero or only a few for some of the mice) as they were only around 5% on average, and therefore all the analyses shown in Fig. 4h–k were performed with pooled data from all of the mice (*N* = 7 mice).

To evaluate the co-activation of neural activity in the dmPFC network, we calculated cell-to-cell pairwise correlations within each ensemble using Pearson’s correlation coefficient, from the GCaMP6f signals (z-normalized ΔF/F) of 2 cells over the duration of the CS+ presentation, as described before^33^. The calculated correlation coefficients were statistically analyzed. As a complementary analysis, we also used the inferred spike probability to analyze the functional connectivity, as explained in the section describing the CRF model, which revealed consistent results as shown in the Results. We further performed analyses based on shuffled datasets, as described in previous studies^33,40^ to discuss the possibility that changes in the basal activity contribute to the change in the correlation coefficient. For this, while the total activity of each neuron was preserved, only the timing of the neural activity was randomly shuffled within each neuron, followed by the calculation of the correlation coefficients between neurons.

### Extraction of neuronal ensembles by the elastic net

To systematically extract a group of neurons (ensemble) encoding the CR (i.e., suppressed locomotion triggered by CS+ as a result of the memory retrieval), or that encoding the RS (i.e., stationary state during no CS presentation), we used the elastic net^32^, a regularization and variable selection algorithm based on the regression model. Models were fitted on neural population activities (Fig. 2) to calculate the likelihood of locomotion state at each time point with high accuracy. Sparse models that rely on the activities of a limited number of neurons were produced by the elastic net, which enabled the selection of neurons informative for estimating the corresponding locomotion states, the CR (Figs. 2d–h, 3f–h and Supplementary Fig. 6) or the RS (Fig. 3 and Supplementary Fig. 7). Because this method allowed us to identify different ensembles for different locomotion states independently from the same group of neurons, we used it to verify whether neurons in the CR ensemble were unique or mostly overlapped with the RS ensemble (Fig. 3). For the elastic net, we used the “lassoglm” function of MATLAB R2019b. The hyperparameter “alpha (*α*)” for the elastic net enables adjustment of the size of the selected neurons depending on the data, and lowering the alpha value tends to increase their inclusion (Supplementary Fig. 6).

When extracting the CR ensemble, we used neural activity data only during the CS+ presentation of D4-early (i.e., when mice showed the CR as a result of memory retrieval) and identified neurons informative for distinguishing whether animals exhibited CR (freezing-like response) or were locomoting during the CS+ (Fig. 2). Therefore, the auditory information of the CS was not used to build the elastic net model for extracting the CR ensemble. While mice exhibited the CR as suppressed locomotion during D4-early (Fig. 1), they locomoted intermittently during the CS+ presentation. Both labels (CR and locomotion) are required to perform the regression based on the elastic net; only mice for which the data contained at least 10% of each label (CR and locomotion) during the CS+ presentation were used to extract the CR ensemble. On the other hand, to extract the RS ensemble, we used data only during the no-CS period (whole D3 or whole D4 data, respectively), including the initial term (before the first CS presentation on each day) and the inter-trial interval (which includes both the pre-CS+ and pre-CS− terms without any bias), excluding 30-sec data after each CS+/CS− trial (when the neural representation might still be affected by the preceding CS).

Learning for the elastic net was formulated as follows:

1 $$\mathop{\min }\limits_{{\beta }_{0},{{{{{\boldsymbol{\beta }}}}}}}\left(\frac{1}{N}\mathop{\sum }\limits_{i=1}^{N}\left(-{y}_{i}\log {\widetilde{y}}_{i}-\left(1-{y}_{i}\right)\log \left(1-{\widetilde{y}}_{i}\right)\right)+\gamma {P}_{\alpha }\left({{{{{\boldsymbol{\beta }}}}}}\right)\right),$$

where

2 $${\widetilde{y}}_{i}=\frac{1}{1+\exp \left(-\left({\beta }_{0}+{{{{{{\bf{x}}}}}}}_{i}^{T}{{{{{\boldsymbol{\beta }}}}}}\right)\right)},$$

3 $${P}_{\alpha }\left({{{{{\boldsymbol{\beta }}}}}}\right)=\frac{(1-\alpha )}{2}{\|{{{{{\boldsymbol{\beta }}}}}}\|}_{2}^{2}+\alpha {\|{{{{{\boldsymbol{\beta }}}}}}\|}_{1}=\mathop{\sum }\limits_{j=1}^{p}\left(\frac{(1-\alpha )}{2}{\beta }_{j}^{2}+\alpha \left|{\beta }_{j}\right|\right).$$

*N* is the number of observations (time points); *y*_*i*_ is the state (CR/stationary *y*_i_ = 1 or locomotive *y*_*i*_ = 0) at observation *i *(*i* = 1,…,*N*); **x**_*i*_ is data (neuronal activity), a *p*-dimensional vector (neural activities of *p* neurons) at observation *i*; *T* denotes the transpose of a vector; *γ* is a positive regularization parameter; parameters *β*_0_ and ${{{{{\boldsymbol{\beta }}}}}}$ are a scalar variable and a *p*-dimensional vector, respectively. *β*_*j*_ is the coefficient for the corresponding neuron *j* estimated by this model. Because this method is designed to sparsely leave the coefficients *β*_*j*_ for the respective neurons, we could identify neurons with non-zero coefficients as those providing substantial information (i.e., ensemble neurons).

The elastic net is a hybrid of ridge regression and LASSO (Least Absolute Shrinkage and Selection Operator) regularization: when alpha (*α*) = 1, elastic net is the same as LASSO, while as alpha approaches 0, the elastic net approaches the ridge regression. The parameter alpha indicates the *L*^1^ ratio as formulated as the penalty term ${P}_{\alpha }\left({{{{{\boldsymbol{\beta }}}}}}\right)$; it interpolates between the *L*^1^ norm of ${{{{{\boldsymbol{\beta }}}}}}$ and the squared *L*^2^ norm of ${{{{{\boldsymbol{\beta }}}}}}$. Parameter alpha directly controls the sparseness of ${{{{{\boldsymbol{\beta }}}}}}$. Tuning alpha is effective for 2 well-known problems in LASSO^32^. 1) LASSO is sensitive to correlations between variables and can choose 1 if there are 2 highly correlated and useful variables, whereas by tuning alpha the elastic net is more likely to select both useful variables, which leads to more stable variable selection. In other words, highly correlated variables have a coefficient of zero, except for 1 in LASSO, whereas in the elastic net, the highly correlated variables take non-zero values together by tuning alpha. 2) The number of variables that can be selected is limited in LASSO when the amount of training data is small. For example, in LASSO, the maximum number of variables that can be selected is *N* when *N* < *p*. In contrast, the elastic net can increase the number of variables up to *p* by tuning alpha. Thus, in the elastic net, tuning alpha can include (select) variables that may be missed in LASSO. In fact, it appeared that our recording data actually included strongly correlated pairs of variables (neuronal activities) (Supplementary Fig. 10), suggesting that the elastic net is suitable for the present study.

The tuning parameter *γ* controls the overall strength of the penalty. We varied *γ* with 7 values evenly logarithmically spaced between 10^−2.5^ and 10^−3.3^, and the *γ* value with minimum expected deviance, which was calculated by 7-fold cross-validation, was systematically selected for each dataset, at each alpha (for the alpha tuning, a wide range of the alpha values was tested as explained later).

To prepare the template neural activity dataset for modeling while avoiding an imbalance in the number of labels for respective states (e.g., CR or not [locomotive] when extracting the CR ensemble), the same number of neural activity vectors (neural population activities at randomly selected time points) from respective states were resampled (chosen randomly with replacement from the original vector set). A total of 900 samples for each state were used to produce each model. The produced models and non-zero-coefficient neurons varied slightly trial by trial, even with the same template dataset. To accurately define each ensemble (and the non-zero-coefficient neurons), we performed this procedure (random resampling and modeling) 100 times and obtained the distribution of the coefficient values for each neuron (we performed this 20 times, instead, when evaluating the performances of “CR ensemble removed”, “Non-CR ensemble removed”, “RS ensemble removed”, and “Non-RS ensemble removed”, which are explained later, because we additionally repeated whole modeling procedure for them 10 times). Gaussian fitting was performed to define the centroid and 95% confidence interval of each distribution for each coefficient. Then, to build the model, the 95% confidence interval was used to determine whether or not each coefficient was significantly different from zero (enabling us to maintain sparsity), and the centroid was used to define the final coefficients of non-zero coefficient neurons.

The goodness of a model was considered “fitting performance” when we evaluated the performance to estimate locomotion states from neural activity data containing all or part of the training dataset. On the other hand, “decoding performance (or predictability)” was defined as the score obtained by the prediction of the states from data excluding the training dataset.

The obtained model was evaluated by calculating the accuracy or the area under the curve (AUC) of the receiver operating characteristic (ROC) curve, but in the present study, we observed that those scores were very similar to each other (Supplementary Fig. 8). Accuracy denotes the ratio of correct estimations per whole observations (time points). The AUC of the ROC curve, which is another parameter for validating the performance of the model and is suggested to be more accurate when labels are imbalanced, was also calculated.

Based on the above-described procedure, a wide range of alpha values was systematically tested (Supplementary Fig. 6) to find the optimal alpha value. In some previous studies, the alpha value was arbitrarily fixed for the analysis^63–65^. Because the dmPFC neurons in our data, as well as cortical neurons in general, include strongly correlated pairs of variables (neuronal activities), however, it appeared important to carefully optimize alpha when attempting to maximally select informative neurons and further compare them with unselected neurons (CR ensemble vs Non-CR ensemble neurons) or an independently selected group (vs RS ensemble).

Ideally, if all the informative neurons can be selected into the CR ensemble, the remaining neurons should have poor information and show poor fitting performance. According to this idea, to optimize the alpha value and the number of selected neurons, we built a model at each alpha for each mouse (Fig. 2d, g) and compared the difference in fitting performance between “CR ensemble removed” and “Non-CR ensemble removed” (Fig. 2g) by calculating the difference between their AUCs (“AUC CRE-rem” and “AUC nonCRE-rem”; Supplementary Fig. 6a). “AUC CRE-rem” is the AUC value calculated by an elastic net model built with the neurons excluding the original CR ensemble neurons. “AUC nonCRE-rem” is the AUC value calculated by the neurons excluding neurons other than original CR ensemble neurons, randomly selected, and the number of excluded neurons was the same as the number of original CR ensemble neurons (so that the number of neurons used to calculate “AUC nonCRE-rem” was set to be the same as that used for calculating “AUC CRE-rem”). The “AUC difference” (Supplementary Fig. 6a) between those 2 values was calculated to estimate the degree of remaining information, and in principle, we defined the best alpha based on the maximum AUC difference for each mouse independently. In addition, for further statistical evaluation to define the optimal alpha as explained below, we repeated these procedures 10 times for both “AUC CRE-rem” and “AUC nonCRE-rem”.

As shown in Supplementary Fig. 6b, although the fitting performance of the original CR ensembles (i.e., AUC original in Supplementary Fig. 6a) was not drastically affected by the alpha (Supplementary Fig. 6b, middle), the size of the CR ensemble was affected, and a smaller alpha generally resulted in a larger number of selected neurons for each CR ensemble (Supplementary Fig. 6b, top), suggesting that the CR information might be redundantly encoded in the dmPFC as discussed in the main text. Also, the influence of the alpha on the AUC difference was more complicated. As explained above, we defined the best alpha based on the maximum AUC difference for each mouse independently, but in some cases, as shown in Supplementary Fig. 6d (mouse #3), the other alpha(s) had an AUC difference(s) that was not significantly different from the maximum AUC difference. In such cases, the alpha of the smallest ensemble size among those alphas, i.e., the largest alpha among them, was selected to avoid unnecessarily including additional neurons that did not improve the AUC difference (e.g., in mouse #3, alpha = 0.1, 0.05, 0.01 showed similar AUC differences and there was no statistically significant difference among them [Wilcoxon rank-sum test, alpha of maximum AUC difference vs the other alpha, *n* = 10 estimates for each calculated as explained above], so in this case, the largest alpha 0.1 among those 3 was selected to define the CR ensemble for this mouse).

These results revealed two important points. First, searching around the alpha value is important in some cases. Considering this, we also screened several alphas for the RS ensembles (Supplementary Fig. 7), and found no difference among the various alphas for the RS ensembles, even if we tested an additional number of reference frames (means of the neural activities over several past or future frames were used as neural activity data to estimate a single label at each time point; the Friedman test, a nonparametric statistical test similar to the parametric one-way repeated measures ANOVA, revealed no significant difference between them). Therefore, in the present study, we fixed the alpha to define RS ensembles at 0.75 for most of the analyses, except for the data in Supplementary Figs. 7 and 8 where we evaluated the influence of the alpha on RS ensembles.

Second, fear memory triggering the CR might be redundantly encoded in the dmPFC. As discussed above, although the fitting performance of the original CR ensemble was not affected by alpha (Supplementary Fig. 6b, middle), the size of the CR ensemble was affected, and a smaller alpha generally resulted in a larger number of selected neurons for each CR ensemble (Supplementary Fig. 6b, top). In addition, when the alpha was fixed at alpha (*α*) = 0.9 (*α* > 0.9 did not work for some mice in our data), although the uniqueness of the CR ensembles was maintained and the ratio of the CR ensemble neurons overlapping with RS ensembles was 26.84% (Supplementary Fig. 6e), which was very similar to the case of alpha-optimized CR ensembles (Fig. 3), the size of this CR ensemble (*α* = 0.9) was 2 times smaller than that of the alpha-optimized CR ensembles (Supplementary Fig. 6f). Importantly, 97.82% of the neurons selected at *α* = 0.9 were also selected in the alpha-optimized CR ensembles (Supplementary Fig. 6f), suggesting that the neurons selected at the largest alpha might be more reliable and robust for decoding among all informative neurons. In addition, even after the removal of such “core” neurons, the remaining neurons also possessed CR-related information (Supplementary Fig. 6b, d), indicating that the CR information was redundantly encoded in the dmPFC.

To evaluate the dominance of the CR ensembles vs the RS ensembles, we applied the RS model to predict the CR or the locomotion states during CS+ before and after fear conditioning (Fig. 3b–d and Supplementary Fig. 8). We also investigated the overlap between them (Fig. 3e–g).

In previous studies, unsupervised algorithms, such as conventional Principal Component Analysis and other dimensional reduction algorithms, were widely used to visualize the neural representation embedded in the neural population activities^14,66^. These algorithms, however, do not directly indicate the neurons corresponding to respective locomotion states. Also, some recent studies introduced unsupervised methods to extract subgroups of neurons by detecting the neurons within a population that repeatedly coactivated^41,67^, but in the present study, this procedure did not work well to extract a repeated pattern of neural population activity that accurately explained all of the CR events. Moreover, because we attempted to evaluate the similarity and overlap between neural ensembles encoding distinct locomotion states, we eventually selected the elastic net, which allows independent identification of neural populations encoding distinct locomotion states as performed in the present study.

### Conditional random field models to evaluate functional connectivity and cellular decoding performance

To evaluate changes in the functional connectivity between neurons and cellular decoding performance for the CS (CS+ or CS−) in the recorded neural population, we used CRFs as described previously^41,42^, which model the conditional probability distribution of the interaction among neuronal ensemble members, as described below.

We used CRFs to capture the contribution of specific neurons to the overall network activity defined by population vectors belonging to a given neuronal population. We generated a graphical model in which each node represents a neuron in a given neural population and edges represent the dependencies between neurons, which enabled us to estimate the functional connectivity between dmPFC neurons that were simultaneously recorded (~300 neurons).

For this analysis, the spike probabilities were inferred from the ΔF/F as an alternative estimate of neuronal activation, which was performed as explained under the subheading “Imaging data analyses and statistics”. After thresholding the spike probability (2 standard deviations), the obtained binary data were further binned (bin size: 1 sec) for the CRF modeling. To train the model, we used 80% of the time points of the activity data (of all simultaneously recorded neurons), which were randomly selected from all-time points, and the remaining 20% was used for the cross-validation to evaluate the fitting of the constructed model and eventually select the best model.

We constructed a CRF model in two steps: (1) structure learning, and (2) parameter learning. For structure learning, we generated a graph structure using *L*^1^-regularized neighborhood-based logistic regression^42^. Here, ${\lambda }_{S}$ is a regularization parameter that controls the sparsity (or conversely, the density) of the constructed graph structure, leaving only relevant functional connectivity, including both coactive and suppressive relationships. Also, a previous study showed that this number of connections is enhanced as a result of optogenetic induction of the rewiring of the local network^42^, demonstrating the reliability of the functional connectivity estimated by CRF models. In the present study, we calculated the ratio of these relevant connections (both coactive and suppressive) per all possible connections for each node as a “functional connectivity score” for each neuron. Before comparing the functional connectivity between different ensembles (e.g., within-CR-ensemble vs within-Non-CR-ensemble) or different cell types (e.g., US-responsive neurons vs Non-US-responsive neurons), we first calculated the whole network connectivity (using all simultaneously recorded neurons) without stratifying by ensembles or cell types, and further separated them into different categories.

The CRF modeling also allowed us to evaluate the information encoded by individual neurons^41,42^. In the present study, to measure the information for a given stimulus (CS+ or CS−), we computed the standard ROC, taking as ground truth the timing of a particular CS. The AUC from the ROC curve that represents the performance of each neuron was calculated and used to compare the encoded information in different ensembles, different neuron types, and different days (e.g., D3 fear conditioning session vs D4 post-FC session). As recently demonstrated^41^, high ranks for this value in the neural population indicate high potential to recall the neural and cognitive representation of a given stimulus.

The following is a detailed description for constructing the CRF model, as reported in previous studies^41,42^. The ${{{{{{\mathcal{X}}}}}}}^{N}$ and ${{{{{{\mathcal{Y}}}}}}}^{N}$ describe an N-dimensional space where the dimensionality describes the total number of active neurons. We used indicator feature vectors $x=[{x}^{1},{x}^{2},\ldots,{x}^{M}]$, where ${x}^{m}\in {{{{{{\mathcal{X}}}}}}}^{N}$, for each edge and node, and target binary population activity vectors $y=[{y}^{1},{y}^{2},\ldots,{y}^{M}]$, where ${y}^{m}\in {{{{{{\mathcal{Y}}}}}}}^{N}$ for N neurons and M samples (time points). For each sample, the conditional probability can be expressed as follows:

4 $$p\left({y}^{m} | {x}^{m}{{{{{\rm{;}}}}}}\, \theta \right)=\frac{\exp (\left\langle \phi \left({x}^{m},{y}^{m}\right),\theta \right\rangle )}{Z({x}^{m}{{{{{\rm{;}}}}}}\, \theta )},$$

where $\phi$ is a vector of the distribution expressed in log-linear form, $\theta$ is a vector of parameters with parameters for the log-linear distribution, and $Z$ is the partition function as follows:

5 $$Z\left({x}^{m}{{{{{\rm{;}}}}}}\, \theta \right)=\mathop{\sum}\limits_{y{{{{{\mathscr{\in }}}}}}{{{{{\mathcal{Y}}}}}}}\exp (\left\langle \phi \left({x}^{m},\, y\right),\, \theta \right\rangle ).$$

The conditional probability can be factored over a graph structure $G=(V,{{{{{\mathcal{A}}}}}})$, where $V$ is the collection of nodes representing observation variables and target variables, and ${{{{{\mathcal{A}}}}}}$ is the collection of subsets of $V$. Based on the graph structure, the model parameters can be written separately for nodes and edges as $\phi=\left\{{\phi }_{V},{\phi }_{A}\right\}$ and $\theta=\{{\theta }_{V},{\theta }_{A}\}\in {{\mathfrak{R}}}^{N}$, respectively. Given binary *x* and *y*, node parameters ${\phi }_{V}$ and ${\theta }_{V}$ include 2 sets of distributions and the parameters corresponding to the node state 0 and 1, whereas edge parameters ${\phi }_{A}$ and ${\theta }_{A}$ include 4 sets of distributions and the parameters, corresponding to the edge states 00, 01, 10, and 11. The conditional dependencies can then be written as follows:

6 $$p\left(Y | X{{{{{\rm{;}}}}}}\, \theta \right)=\frac{\exp \big({\sum}_{i\in V}{\theta }_{i}{\phi }_{i}\left(X,{Y}_{i}\right)+{\sum}_{\alpha {{{{{\mathscr{\in }}}}}}{{{{{\mathcal{A}}}}}}}{\theta }_{\alpha }{\phi }_{\alpha }\left(X{{{{{\boldsymbol{,}}}}}}{Y}_{\alpha }\right)\big)}{Z(X{{{{{\rm{;}}}}}}\, \theta )}.$$

This model is a generalized version of Ising models, which have been applied previously for modeling neuronal networks^68^. The log likelihood of each observation can then be written as follows:

7 $${{{{{\mathcal{l}}}}}}\left(\theta {{{{{\rm{;}}}}}}\, {X}^{m},\, {Y}^{m}\right)=\left\langle \phi \left({X}^{m},\, {Y}^{m}\right),\, \theta \right\rangle -\log Z({X}^{m}).$$

To reduce the complexity of the model, we first learned a sparse graph structure that represents variable dependencies, and then constructed a CRF on the learned structure. Given the inferred binary spikes from raw imaging data, we constructed CRF models in 2 steps: (1) structure learning and (2) parameter learning.

For structure learning, we learned a sparse graph structure $G=(V,\, {{{{{\mathcal{A}}}}}})$ using *L*^1^-regularized neighborhood-based logistic regression for each node *r*^69^ as follows:

8 $$\mathop{\min }\limits_{{\theta }_{{{\backslash }}r}}\left\{{{{{{{\mathcal{l}}}}}}\left({\theta }^{s}{{{{{\rm{;}}}}}}\, x\right)+{\lambda }_{s}\|{\theta }_{{{\backslash }}r}^{s}\|}_{1}\right\},$$

where

9 $${{{{{\mathcal{l}}}}}}\left({\theta }^{s}{{{{{\rm{;}}}}}}\, x\right)=-\frac{1}{n}\mathop{\sum }\limits_{i=1}^{n}\log \frac{\exp \left(2{x}_{r}{\sum}_{t\in V{{\backslash }}r}{\theta }_{{rt}}^{s}{x}_{t}\right)}{\exp \left(2{x}_{r}{\sum}_{t\in V{{\backslash }}r}{\theta }_{{rt}}^{s}{x}_{t}\right)+1}$$

and

10 $${\theta }_{{{{{{\rm{\backslash }}}}}}r}^{s}=\left\{{\theta }_{{ru}}^{s},\, u\in V{{\backslash }}r\right\},$$

where *\r* denotes except *r*, and ${\theta }^{s}$ is a vector of regression parameters for structure learning. Here, ${\lambda }_{s}$ is a regularization parameter that controls the sparsity (or conversely, the density) of the constructed graph structure. This is essentially a logistic regression of variable ${X}_{r}$ on the other variables ${X}_{{\backslash r}}$, with *L*^1^-regularization. The regression coefficients thus represent the neighborhood structure and the sign pattern. We implemented *L*^1^-regularization as described previously^41,42^. We constructed a large number of structures (100+) across a broad range of ${\lambda }_{s}$, from which we stochastically selected a number of structures for subsequent parameter learning. We ensured proper bracketing by including the largest and smallest ${\lambda }_{s}$ structure in this priming set. In further iterations of parameter learning, the sets of the obtained structures were systematically tested and the best model was selected using the log likelihood of the learned models as feedback.

For parameter learning, we aimed to learn the potential node and edge parameters ($\phi$ and $\theta$). Based on the learned structure, we used the Bethe approximation to approximate the partition function and iterative Frank-Wolfe methods for parameter estimation by maximizing the log likelihood of observations with a quadratic regularizer^70^ as follows:

11 $${{{{{\mathcal{l}}}}}}\left(\theta {{{{{\rm{;}}}}}}\, X,\, Y\right)=\mathop{\sum }\limits_{m=1}^{M}{{{{{\mathcal{l}}}}}}\left(\theta {{{{{\rm{;}}}}}}\, {X}^{m},\, {Y}^{m}\right)-\frac{{\lambda }_{p}}{2}{\|\theta \|}^{2}.$$

Here, ${\lambda }_{p}$ is a regularization parameter that controls the learned parameters and helps prevent overfitting. Cross-validation was performed to find the best ${\lambda }_{s}$ and ${\lambda }_{p}$ via held-out model likelihood. We varied ${\lambda }_{s}$ with 100 values (between 0.00001 and 0.5, sampled uniformly) and ${\lambda }_{p}$ with 4 values (1, 10, 100, and 10,000). To obtain the best model parameters, 80% of the data were used for training, and 20% of the data were withheld for validation. Cross-validation was performed by using all possible combinations of the above parameters and calculating the likelihood of the withheld data. Then, the best model parameters were determined by selecting the parameter set with a locally maximum likelihood in the parameter space.

We also evaluated the cellular contribution for predicting the stimulus conditions (CS+ and CS−) in the population, as described previously^41,42^. For the *i*^th^ neuron in the population, we compared its activity or inactivity in all *M* imaging frames (time points) for each model. With the 2 resulting population vectors in the *m*^th^ frame among all samples, we calculated the log probability of them coming from the trained CRF model as follows:

12 $${p}_{i,1}^{m}=p({y}^{m}{{{{{\rm{|}}}}}}{x}_{{{\backslash }}i}^{m},\, {x}_{i}^{m}=1{{{{{\rm{;}}}}}}\, \theta )$$

13 $${p}_{i,0}^{m}=p({y}^{m}{{{{{\rm{|}}}}}}{x}_{{{\backslash }}i}^{m},\, {x}_{i}^{m}=0{{{{{\rm{;}}}}}}\, \theta ).$$

Then, we computed the log-likelihood ratio as follows:

14 $${{{{{{\mathcal{l}}}}}}}_{i,1-0}=\left\{\log {p}_{i,1}^{m}-\log {p}_{i,0}^{m}\right\},\, m=1,\ldots,M\,$$

and calculated the standard ROC curve with the ground truth as the timing of each tone (CS+, CS−). The prediction ability of all nodes (neurons) for all presented stimuli (tones) is then represented by an AUC matrix *A*, where ${A}_{i,d}$ represents the AUC value of node *i* predicting tone *d*. Additionally, we calculated the node strength $S=\{{s}_{i}\}$ of each neuron in the CRF model. We then computed the predicted AUC ${A}^{r}$ and node strength ${S}^{r}$ of each node *r* from 100 CRF models trained on shuffled data. The final ensemble for tone *d* is defined as follows:

15 $$\{i{{{{{\rm{|}}}}}}{A}_{i,d} \, > \, {{{{{\rm{mean}}}}}}\left({A}_{d}^{r}\right)+{{{{{\rm{std}}}}}}\left({A}_{d}^{r}\right),\, {S}_{i} \, > \, {{{{{\rm{mean}}}}}}\left({S}^{r}\right)+{{{{{\rm{std}}}}}}\left({S}^{r}\right)\}.$$

### Statistical analysis

Statistical analyses in the present study were performed as described above (in “Methods” as well as in the main text and figure legends).

### Reporting summary

Further information on research design is available in the Nature Portfolio Reporting Summary linked to this article.

### Supplementary information

Supplementary Information

Description of Additional Supplementary Files

Supplementary Movie 1

Supplementary Movie 2

Reporting Summary

### Source data

Source Data

## Data Availability

Source data files are provided with this paper. The data that support the findings of this study are also available from the corresponding author upon reasonable request. The anatomical information in the Allen Brain Atlas (https://atlas.brain-map.org/) was used for the anatomical description, determination of the virus injection area, and evaluation of the recorded brain regions. Source data are provided with this paper.

## Code availability

For data analyses in the present study, commercial software and publicly available codes were used (details are described in the Methods section). For the statistical analysis, we used MATLAB R2014a and R2019b (MathWorks, Natick, MA). Graphs were produced by MATLAB R2014a/R2019b (MathWorks) and Excel (Microsoft). When plotting the entire large number of samples, we used a MATLAB code, CategoricalScatterplot (https://github.com/AbstractGeek/CategoricalScatterplot). Raw images of the GCaMP6f signals in the dmPFC were processed to correct for brain motion artifacts, using the publicly available code for the enhanced correlation coefficient image alignment algorithm (Evangelidis and Psarakis, 2008; https://jp.mathworks.com/matlabcentral/fileexchange/27253-ecc-image-alignment-algorithm-image-registration). The ROIs for the detection of neural activity were automatically selected using a constrained nonnegative matrix factorization algorithm in MATLAB R2014a/R2019b, and the code is also publicly available (Pnevmatikakis et al., 2016; https://github.com/flatironinstitute/CaImAn-MATLAB). For inferring the spike probabilities from the ΔF/F as an alternative estimate of neuronal activation, the publicly available code “constrained_foopsi.m” (Pnevmatikakis et al., 2016; https://github.com/flatironinstitute/CaImAn-MATLAB) was used. For the elastic net, we used the “lassoglm” function of MATLAB R2019b. For the CRF modeling, we used the publicly available code developed previously (Carrillo-Reid et al., 2019; Carrillo-Reid et al., 2021; https://github.com/hanshuting/graph_ensemble; https://github.com/darikoneil/Identification-of-Pattern-Completion-Neurons).
