# Supplementary material for: Activity-dependent organization of prefrontal hub-networks for associative learning and signal transformation
Source: Nat Commun. 2023 Oct 6;14:5996. doi: 10.1038/s41467-023-41547-5 (PMC10558457; doi:10.1038/s41467-023-41547-5)
Supplement: Supplementary file 1 — Supplementary Information [file 41467_2023_41547_MOESM1_ESM.pdf]

Supplemental information for

Activity-dependent organization of prefrontal hub-networks for associative learning and signal transformation

Masakazu Agetsuma[1, 2, 3, 4, 5] *, Issei Sato[6], Yasuhiro R Tanaka[7], Luis Carrillo-Reid[8], Atsushi Kasai[9], Atsushi Noritake[10], Yoshiyuki Arai[3], Miki Yoshitomo[1], Takashi Inagaki[1], Hiroshi Yukawa[5], Hitoshi Hashimoto[9, 11, 12, 13, 14], Junichi Nabekura[1], and Takeharu Nagai[3]

1, Division of Homeostatic Development, National Institute for Physiological Sciences, 38 Nishigohnaka Myodaiji-cho, Okazaki, Aichi, 444-8585, Japan

2, Japan Science and Technology Agency, PRESTO, 4-1-8 Honcho, Kawaguchi, Saitama, 332-0012, Japan

3, SANKEN (The Institute of Scientific and Industrial Research), Osaka University, Mihogaoka 8-1, Ibaraki, Osaka 567-0047, Japan

4, Division of Molecular Design, Research Center for Systems Immunology, Medical Institute of Bioregulation, Kyushu University, 3-1-1 Maidashi, Higashi-ku, Fukuoka 812-8582,

5, Quantum Regenerative and Biomedical Engineering Team, Institute for Quantum Life Science, National Institutes for Quantum Science and Technology (QST), Anagawa 4-9-1, Chiba Inage-ku, Chiba 263-8555, Japan

6, Department of Computer Science, Graduate School of Information Science and Technology, The University of Tokyo. 7-3-1 Hongo, Bunkyo-ku, Tokyo 113-0033, Japan.

7, Brain Science Institute, Tamagawa University, 6-1-1 Tamagawagakuen, Machida, Tokyo, 194-8610, Japan

8, Instituto de Neurobiologia, National Autonomous University of Mexico, Boulevard Juriquilla 3001, Juriquilla, Queretaro, CP 76230, Mexico

9, Graduate School of Pharmaceutical Sciences, Osaka University, Yamadaoka 1-6, Suita, Osaka 565-0871, Japan

10, Division of Behavioral Development, National Institute for Physiological Sciences, 38 Nishigohnaka Myodaiji-cho, Okazaki, Aichi, 444-8585, Japan

11, Institute of Nano-Life-Systems, Institutes of Innovation for Future Society Nagoya University, Furo-cho, Chikusa-ku, Nagoya 464-8603, JAPAN

12, United Graduate School of Child Development, Osaka University, Kanazawa University, Hamamatsu University School of Medicine, Chiba University, and University of Fukui, 2-2 Yamadaoka, Suita, Osaka 565-0871, Japan

13, Division of Bioscience, Institute for Datability Science, Osaka University, 1-8 Yamadaoka, Suita, Osaka 565-0871, Japan

14, Open and Transdisciplinary Research Initiatives, Osaka University, 2-1 Yamadaoka, Suita, Osaka 565-0871, Japan

15, Graduate School of Medicine, Osaka University, 2-2 Yamadaoka, Suita, Osaka 565-0871, Japan

*Correspondence to: age@nips.ac.jp

This PDF file includes:

Supplementary Figures. 1 to 14

Captions for Supplementary Movies 1 and 2

Other Supplementary Materials for this manuscript include the following:

Supplementary Movies 1 and 2

Supplementary Fig. 1

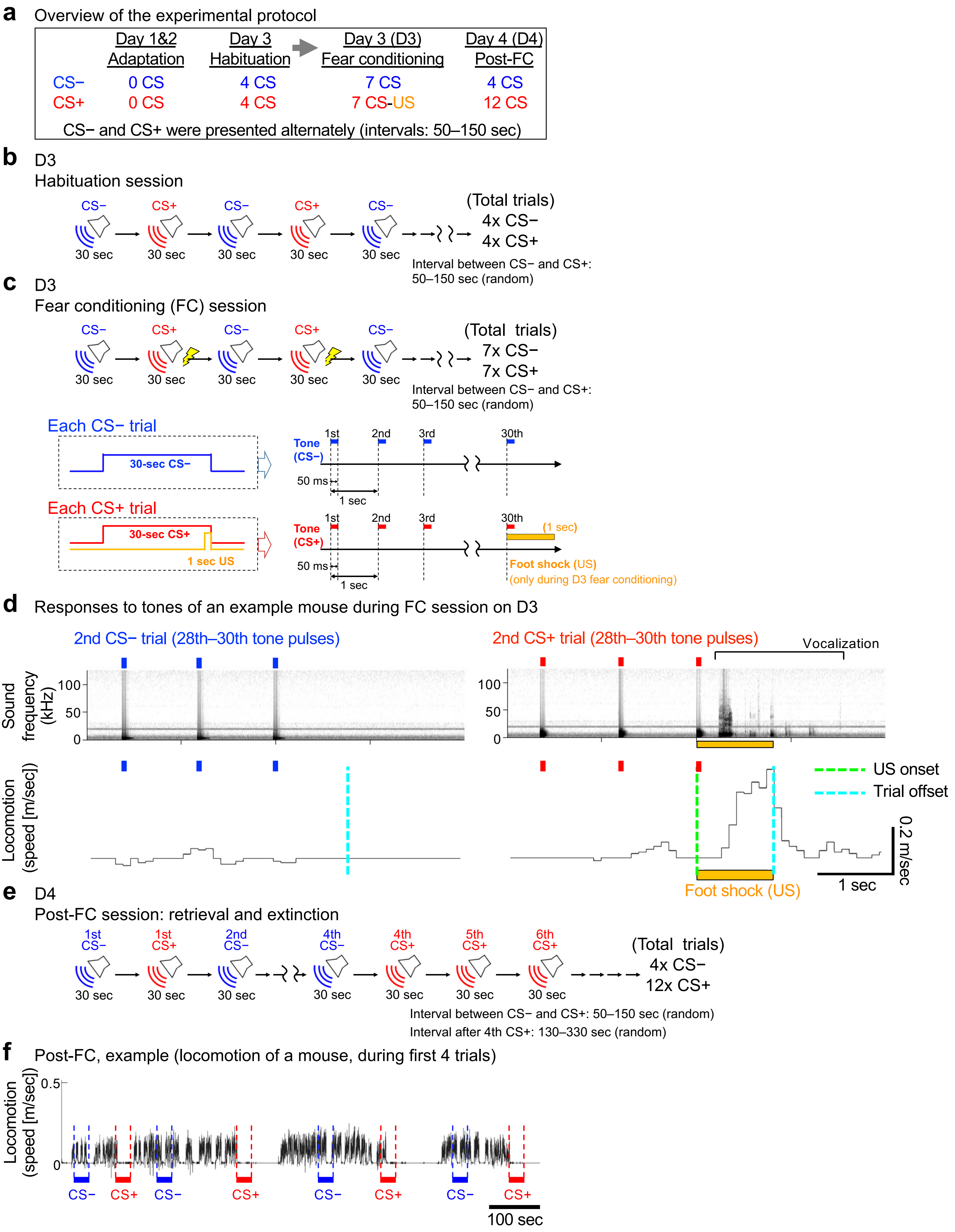

Supplementary Fig. 1. Experimental protocol for days 3 and 4 (D3 and D4): detailed description.

(**a**) Overview of the experimental protocol (for convenience, the same panel as in Fig. 1b (top) is shown). Further details are described in subsequent panels. CS, conditioned stimulus; US, unconditioned stimulus; FC, fear conditioning. (**b**) D3 habituation session. Mice received 4 presentations each of the CS− and CS+ (without the US) alternately. (**c**) (top) The discriminative fear-conditioning (FC) session immediately after the habituation session on the same day. In the FC session, the CS+ was paired with the US. The US duration was 1 sec and it co-terminated with the CS+ trial. The CS− and CS+ trials were performed alternately (inter-trial intervals, 50–150 sec). (bottom) The total CS duration was 30 sec for each CS−/CS+ trial and consisted of 50-ms pips at 1 Hz repeated 30 times. For the CS+ and US pairing (i.e., the fear conditioning), 1-sec US co-terminated with the CS+ trial as described in this panel and in panel d (the onset of the US coincided with the onset of the last sound pip of each 30-sec CS trial). (**d**) The response to the foot shock (and whether or not the intensity was sufficient) was manually evaluated based on the detection of enhanced locomotion and/or vocalizations emitted by the mice immediately after foot shock onset. This panel shows an example of such enhanced locomotion and induced vocalization in a mouse, specifically after the onset of the US. (**e**) The day after the fear conditioning (on D4), the conditioned mice underwent a post-FC session, in which they received 4 presentations of the CS− and 12 presentations of the CS+ without US presentation (4 presentations of the CS− and CS+ trials alternately [inter-trial intervals, 50–150 sec], followed by 8 consecutive CS+ trials [inter-trial intervals, 130–330 sec]). (**f**) The example mouse exhibited a freezing-like response, i.e., decreased locomotion as a conditioned response (CR), specifically during the CS+ presentation (the same panel as Fig. 1b (bottom) shown for convenience). We refer to this expression of the CS+-evoked CRs during the early post-FC session as memory retrieval.

Supplementary Fig. 2

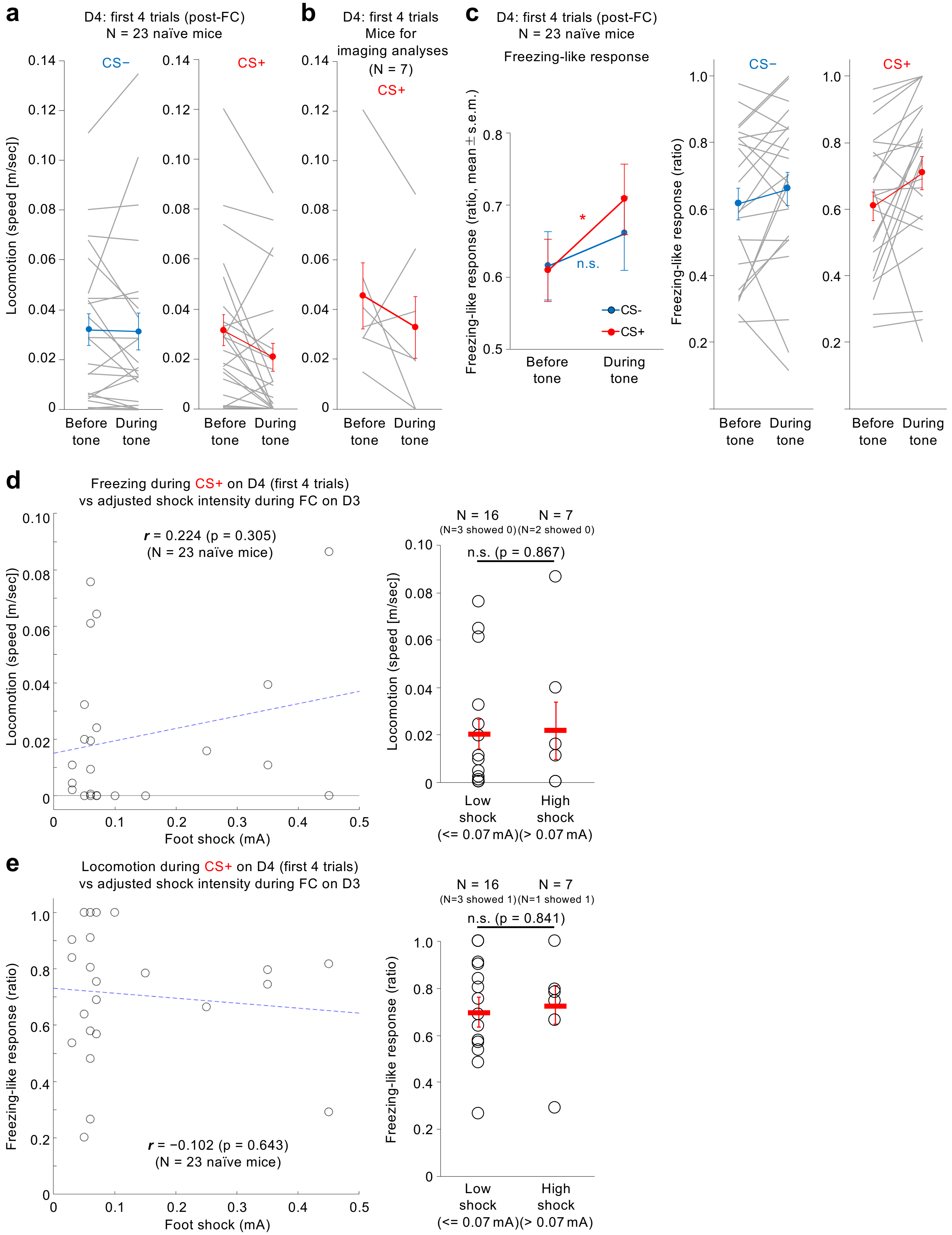

Supplementary Fig. 2. Summary of responses to CS+ and CS− of individual mice on day (D) 3 and D4.

The details of the results of the naïve mice (i.e., non-CNO injected ones) are summarized. (**a**) The change in locomotor speed by the CS+ and CS− presentation at each individual mouse during the first 4 trials on D4 was shown. The data summarized here are the same as Fig. 1c, right panel (N = 23 mice), indicating that most of the mice showed reduced locomotion. (**b**) The CS+-evoked change in locomotion of the mice used for the longitudinal imaging during D3 and D4 (N = 7) is described, which was consistent with the change in locomotion of the entire cohort mice (shown in panel a). (**c**) (left) The freezing-like response was scored by referring to the locomotion speed of mice. With this measure, we also confirmed the significant enhancement of this freezing-like response by the CS+ presentation, but not by the CS−. Wilcoxon signed-rank test (nonparametric paired test) was used for the statistical comparison. (middle and right) The change in the freezing-like response by the CS+ (middle) and CS− (right) presentation at each mouse during the first 4 trials on D4 was also shown. (**d–e**) Although we used a milder foot shock current for most of the experiments, we sometimes needed to increase the foot shock intensity when mice failed to respond to the US, likely due to the running disk becoming dirty or wet from the mice and thus possibly suppressing or shunting the flow of current during the experiment (see Methods for details). Therefore, in these panels, we investigated the relationship between the foot shock level during the fear conditioning and the behavior during the memory retrieval (locomotion speed [d] or freezing-like response [e]) on D4 (during the first 4 trials). (left) The relationship between the foot shock intensity and the behavior of each mouse was plotted. Using Pearson's correlation and calculating the r and p value, we demonstrated no significant correlation between them. (right) We compared the behavior of the mouse group that experienced a stronger foot shock (0.10–0.45 mA) and the group experiencing a weaker shock (0.03–0.07 mA), using a non-paired comparison (Wilcoxon rank sum test), and observed no significant difference in the freezing-like response and locomotion level during the CS+ between these two groups. Gray lines (a–c) and gray circles (d–e) indicate the results of individual mice. Blue dotted lines correspond to the results of the line fitting (linear regression). Blue and red circles with the error bars in a–c indicate mean and s.e.m., respectively. Red horizontal bars with the error bars in d and e indicate mean and s.e.m., respectively. * $p < 0.05$; n.s., not significant.

Supplementary Fig. 3

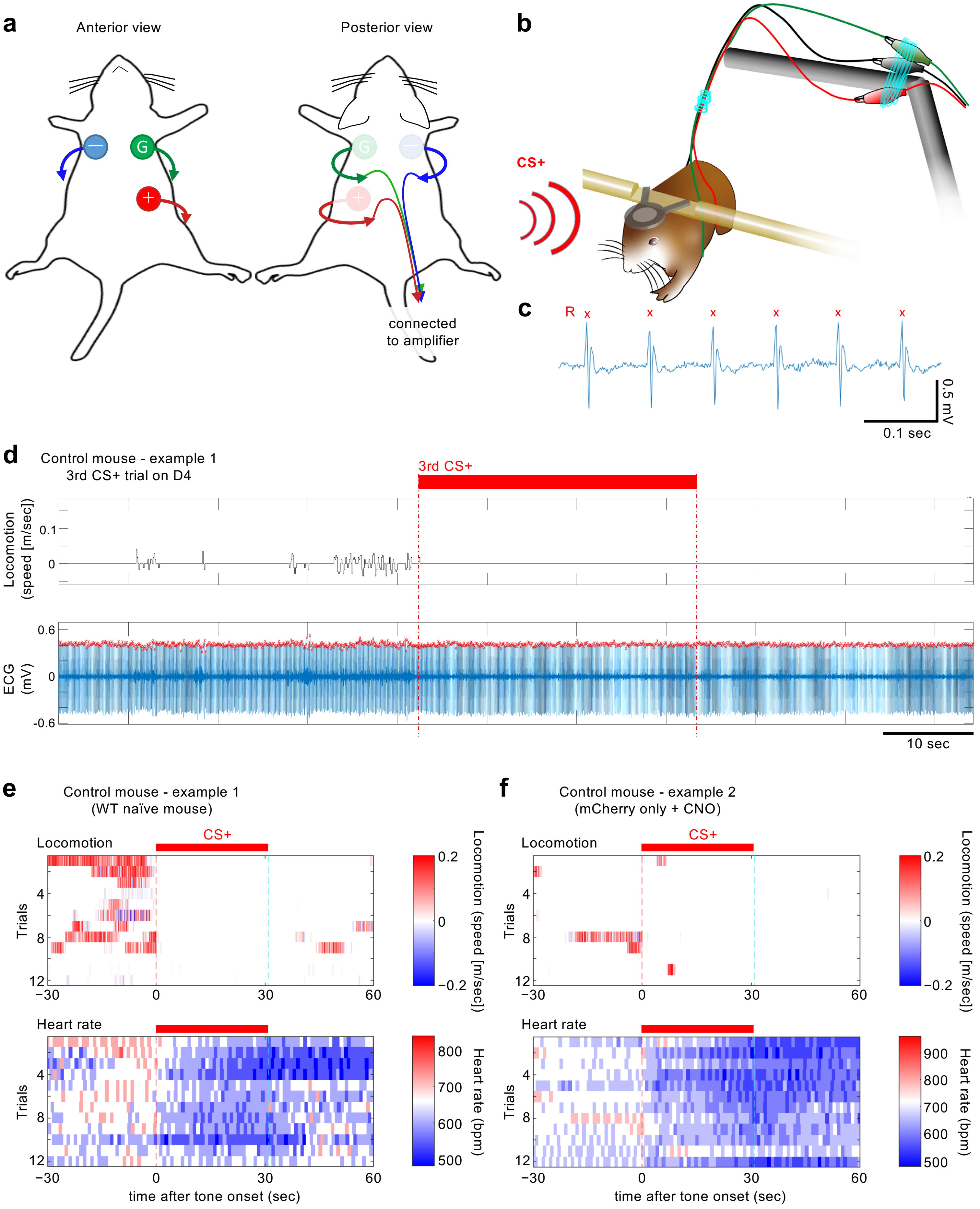

Supplementary Fig. 3. Heart rate recording during the post-FC session indicated that the non-locomotive state during CS+ presentation was accompanied by heart rate deceleration.

(**a**) Schematic diagram showing the positions of the electrodes on the mouse for the electrocardiogram. The negative electrode (−) was placed in the mouse's right upper chest, and the positive electrode (+) was placed in the left abdomen. ECG signals were referenced to the ground (G; left upper chest). (**b**) The cables were bound above the mouse by the plastic tape, and the cable bundle was clipped to a flexible magnetic stand base holder and then connected to the amplifier. (**c**) The typical R-wave peak of each heartbeat was clearly identified (red cross), and the detected peaks were used to calculate the heart rate. (**d–e**) An example mouse showed reduced locomotion and heart rate during CS+ presentation during the D4 post-FC session. Raw traces for locomotion and ECG during the 3rd CS+ trial (d) and calculated locomotion and heart rate for the entire 12 CS+ trials (e) of the same example mouse are shown. Red crosses in panel d indicates the detected R peaks. The reduction of the heart rate, which is reportedly one of the representative features during the freezing, was clearly observed especially during the first four trials. (**f**) Another example mouse (a control mouse used for the DREADD experiments and tested 30 min after the intraperitoneal injection of CNO) showed a low basal locomotion level even without the CS+. In this case, even without a clear change in locomotion level, the mouse showed heart rate deceleration during the CS+, suggesting that a regular stationary state (without CS+) and non-locomotive state during CS+ (i.e., freezing-like response as a CR) might be physiologically maintained differentially. The feature of the heart rate deceleration triggered by the CS+ was further statistically analyzed (as shown in Supplementary Fig. 4).

Supplementary Fig. 4

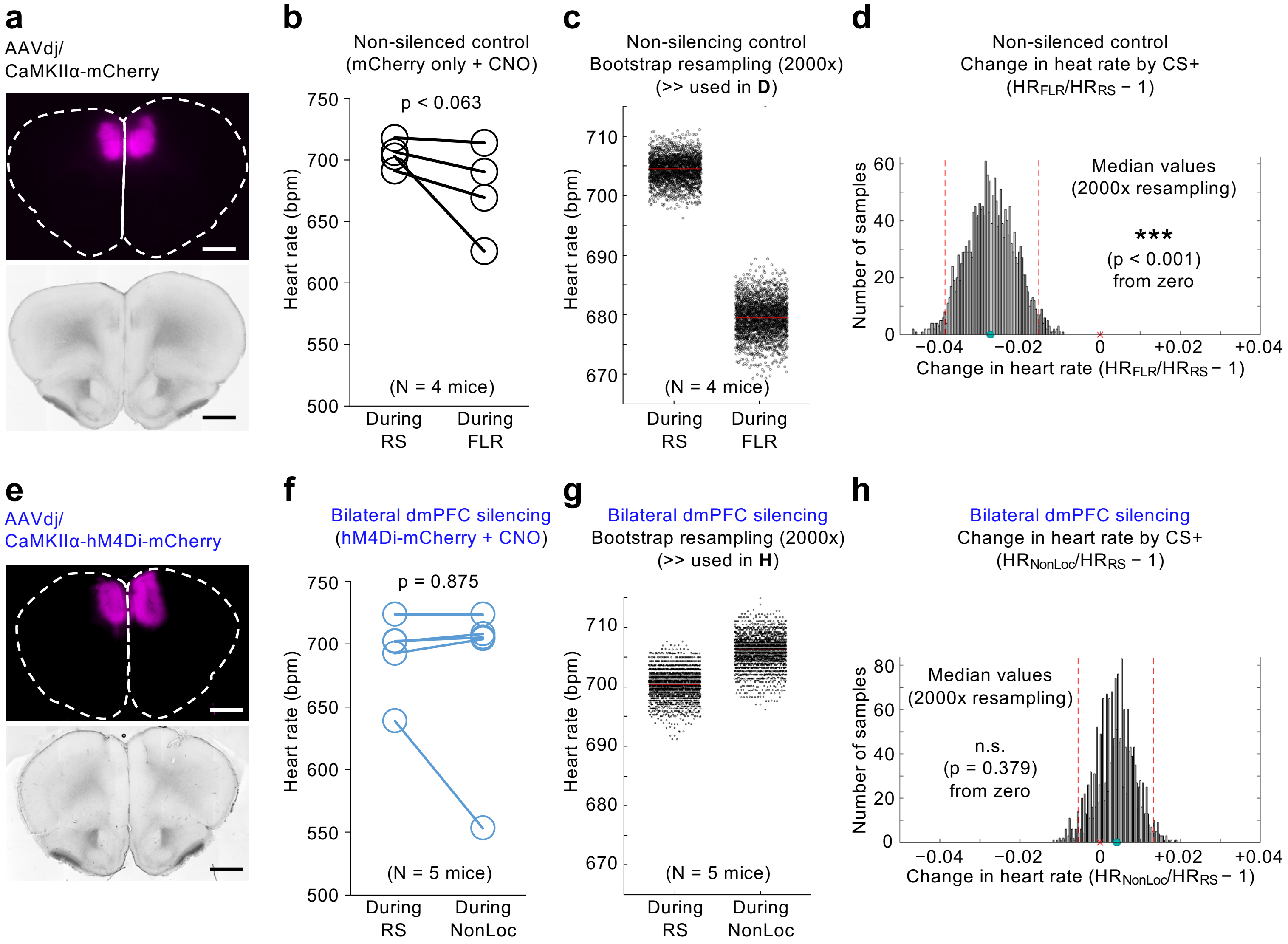

i

Mouse	Ratio difference ($Ratio_{CS+} - Ratio_{CS-}$)	Significance from zero
hM4Di-mCherry #A	-0.001	n.s.
hM4Di-mCherry #B	0.007	n.s.
hM4Di-mCherry #C	-0.010	n.s.
hM4Di-mCherry #D	-0.164	***
hM4Di-mCherry #E	0.011	n.s.
mCherry only #A	-0.136	***
mCherry only #B	-0.062	***
mCherry only #C	0.070	***
mCherry only #D	-0.036	**
mCherry only #E	-0.038	***

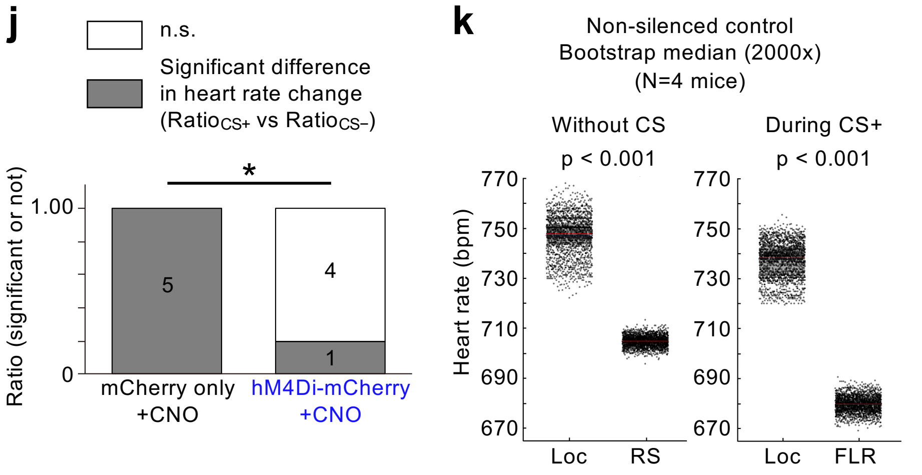

Supplementary Fig. 4. Heart rate reduction during the freezing-like response, and the suppression by the dmPFC silencing

(**a**) A coronal section of a mouse brain injected with AAVdj/CaMKIIα-mCherry as the non-silenced control mice into the bilateral dmPFC. (top) the expression pattern of mCherry. (bottom) the bright field image of the same brain slice. (**b**) In the control mice, the heart rate during the freezing-like response (FLR, in this figure) evoked by the CS+ was slower than the heart rate during the regular stationary state (RS). The p-value obtained by the paired permutation test was less than 0.063, which is the minimum for the comparison of the 4 pairs. (**c**) We performed tests based on bootstrap resampling to more systematically evaluate the difference between the two groups. We first calculated the bootstrap means for each individual, which was further used to calculate the medians (n = 2000 times, shown as 2000 dots in each panel) for each group as shown here. (**d**) From each pair (at each resampling round) of the values calculated in the panel c, a ratio value (heart rate during the FLR was divided by that during the RS) was calculated (in total 2000 values). The p-value was directly calculated from the distribution, and upper and lower boundaries for the statistical significance (red dotted lines, $p < 0.05$), testing the significant difference from zero (zero means that HR_{FLR} is equal to HR_{RS}). (**e**) A coronal section of a mouse injected with AAVdj/CaMKIIα-hM4Di-mCherry into the bilateral dmPFC. (top) The expression pattern of hM4Di-mCherry. (bottom) Bright field image of the same brain slice. (**f**) The paired permutation test for the dmPFC-silenced mice indicated that the heart rates between the non-locomotive state under the CS+ (NonLoc) and the RS were not significantly different. (**g–h**) The analyses based on the bootstrap resampling for the dmPFC-silenced mice revealed no significant difference. (**i**) The impact of the CS+ on the heart rate at each mouse level was evaluated by the bootstrap resampling (See Methods for details). "Ratio difference" was calculated by dividing the 29-sec during-trial heart rate with the 29-sec pre-trial heart rate based on the first 4 trials of either the CS+ or the CS− on D4, and further performed subtraction between $Ratio_{CS+}$ and $Ratio_{CS-}$. (**j**) Fisher's exact test was performed by referring to the results in the panel g, indicating that a statistically significant association between the dmPFC activity and CS+-evoked change in heart rate. (**k**) (left) Without the CS (i.e., no CS+, no CS−), the heart rates during the RS were significantly slower than those during the locomotive state. (right) Under the CS+, the heart rates during the FLR were significantly slower than those during the locomotive state. White dotted lines in a and e indicate the edges of the brain slices. Scale bars in a and e, 1 mm; RS, regular stationary state (without the CS); FLR, freezing-like response under the CS (CS+ or CS−); red bars in c, g and k, median; gray boxes in c, g and k indicate the 25th and 75th percentiles; red dotted lines in d and h, upper and lower boundaries for the statistical significance ($p < 0.05$); green circle in d and h, the median of the distribution; red cross in d and h, zero; HR_{XX}, heart rate during XX; $Ratio_{XX}$, change in HR_{FLR}/HR_{RS} ratio for XX (CS+ or CS−); NonLoc, non-locomotive state; * $p < 0.05$; ** $p < 0.01$; *** $p < 0.001$; n.s., not significant.

Supplementary Fig. 5

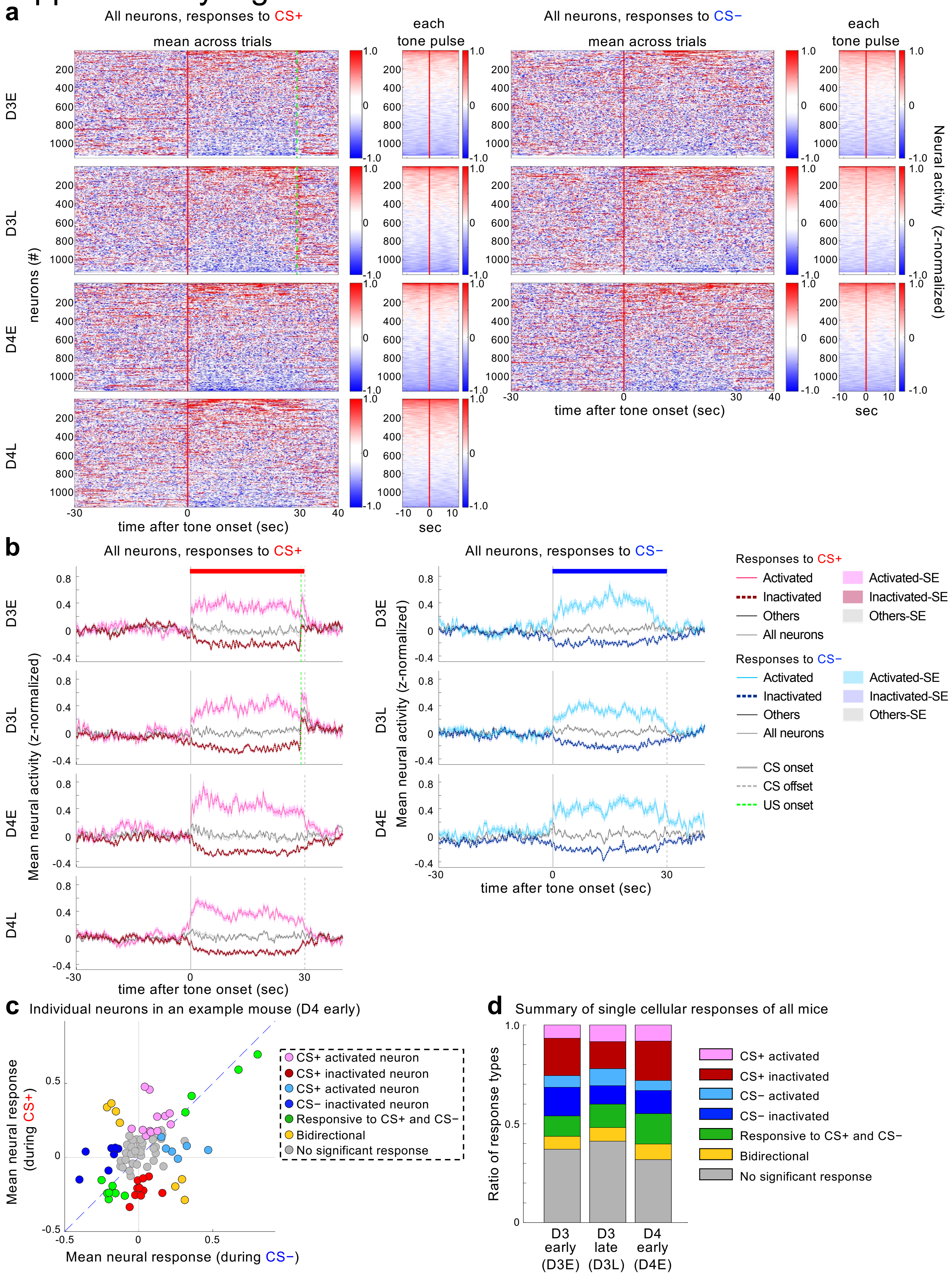

Supplementary Fig. 5. Summary of responses to CS+ and CS− of individual neurons on D3 and D4.

To consider possible temporal changes in responses to the CSs before and after the fear conditioning, only results from mice in which neural activities were successfully recorded on both D3 and D4, from the same sets of neurons, were analyzed. (**a**) Neural activities aligned at the CS onset are ordered by the magnitude of response for 29 sec from the onset of the respective CSs at each temporal phase (D3E/D3L, D4E/D4L). Mean activity over 3 CS trials, or over 87 onsets of 50-ms tone pulses during the 3 trials (D3-early[D3E]/D3-late[D3L] or D4-early[D4E]/D4-late[D4L], respectively) for all individual neurons is plotted separately (n = 1165). (**b**) Mean (± s.e.m. (SE)) CS responses of each category (significantly activated [bright red or blue], inactivated [dark red or blue] by CS+ (left) or CS− (right), and others [dark gray]), as well as the mean of all neurons (light gray), at each temporal phase (D3E/D3L, D4E/D4L), are plotted separately. To categorize the responsiveness of individual neurons, we statistically tested whether the responses of each neuron were significantly different from zero (baseline), and neurons with values significantly higher than the baseline were categorized as "activated" whereas neurons with values significantly lower than the baseline were categorized as "inactivated". "Others" means that these neurons showed no significant change during the CSs. See Methods for details. (**c**) Scatter plot showing responses of individual neurons to the CS+ and CS− in an example mouse during D4E. Each dot represents the mean response of each neuron. Pale red, red, pale blue, blue, green, and yellow colors indicate that neurons had a significant response as described in the panel. "Responsive to CS+ and CS−" indicates the neurons were significantly responsive to both CSs in the same direction (either enhanced or suppressed). "Bidirectional" means that these neurons showed significantly enhanced responses to one of the CSs and significantly suppressed responses to the other CS. See Methods for details. These features for all the mice are summarized in d. (**d**) Summary of response profiles at each phase (D3E, D3L, and D4E, respectively; n = 1165 neurons from N = 7 chronically recorded mice; for each mouse, n = 91, 116, 249, 99, 288, 157, 165 neurons respectively).

Supplementary Fig. 6

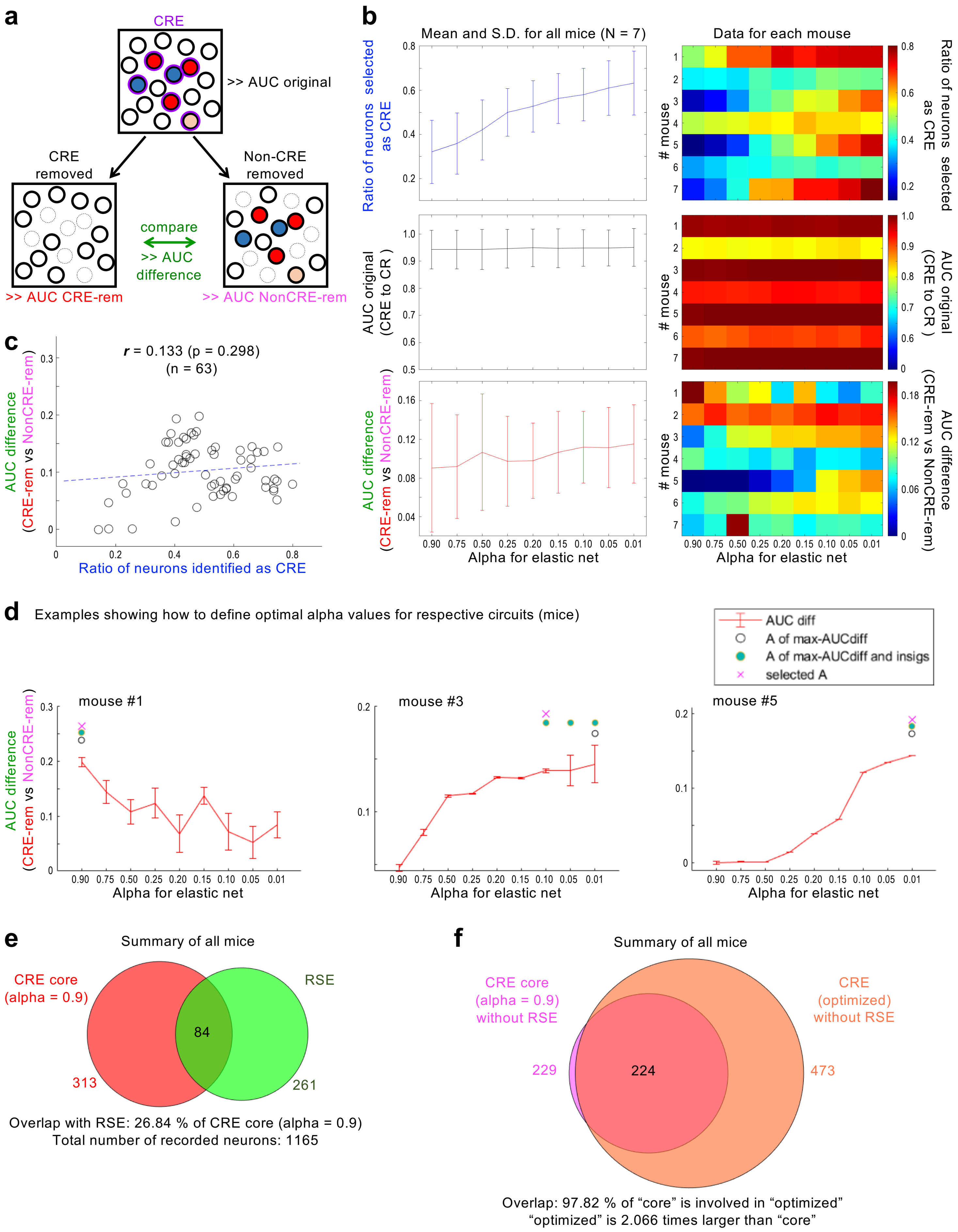

Supplementary Fig. 6. Optimization process of the size of the CR ensemble by adjusting the alpha value for the elastic net, which also revealed the redundant coding feature.

(**a**) We systematically tested a wide range of alpha values and evaluated whether informative neurons were left as unselected neurons at each alpha by measuring the information (fitting performance of a model) of the remaining and unselected neurons. The AUC of the ROC was calculated to accurately evaluate the fitting performance of a model built at each alpha for each mouse. The fitting performance of the obtained model based on the whole original data was calculated as “AUC original”. We further compared the difference in fitting performances between “AUC CRE-rem” and “AUC NonCRE-rem”. AUC CRE-rem is the AUC value calculated by an elastic net model built with the neurons excluding original CR ensemble neurons. AUC NonCRE-rem is the AUC value calculated by the neurons excluding neurons other than original CR ensemble neurons. The “AUC difference” between those two values was further calculated, and in principle, we defined the best alpha based on the maximum AUC difference for each mouse independently (see more details in the Methods and panel d). (**b**) Summary (Mean and S.D., left) and raw data (data for each mouse, right) for ratio of neurons identified as CR ensemble (per whole neurons of each mouse) (top), AUC original (middle), and AUC difference (bottom), at each alpha. (**c**) Pearson’s correlation was used to calculate the *r* and p values, revealing that the ratio of neurons identified as CR ensembles (per whole neurons) and the AUC difference were not significantly correlated (n = 63 samples [7 mice x 9 alphas] were analyzed to determine the possible relationship). The blue dotted line corresponds to the result of the line fitting (linear regression). (**d**) Examples showing how to determine the optimal alpha values for respective circuits (mice). In principle, we defined the best alpha based on the maximum AUC difference for each mouse independently, but in some examples as in mouse #3, several alphas revealed statistically insignificant results among the AUC differences. In this case, the largest alpha among those with the same AUC difference was selected. See more details in the Methods. (**e**) When alpha was fixed at alpha = 0.9, while the number of the neurons selected as CRE neurons in total (7 mice, 1165 neurons) were smaller than the case of the optimized alpha, the ratio of the CRE neurons that overlapped with RSE neurons was 26.84%, similar to the case of the optimized alpha as shown in Fig. 3. (**f**) The size of this CR ensemble (alpha = 0.9) was about two times smaller than that of the alpha-optimized CR ensembles. 97.82% of the neurons identified at alpha = 0.9 were also selected in the alpha-optimized CR ensembles, suggesting that the neurons selected at the largest alpha 0.9 might be more reliable and robust for the estimation among all the informative neurons in the dmPFC. In addition, even after the removal of such “core” neurons, the remaining neurons also possessed information for the CR (as shown in b and d), indicating that the CR information was redundantly encoded in the dmPFC. See more details in the Discussion. Error bars, s.e.m.

Supplementary Fig. 7

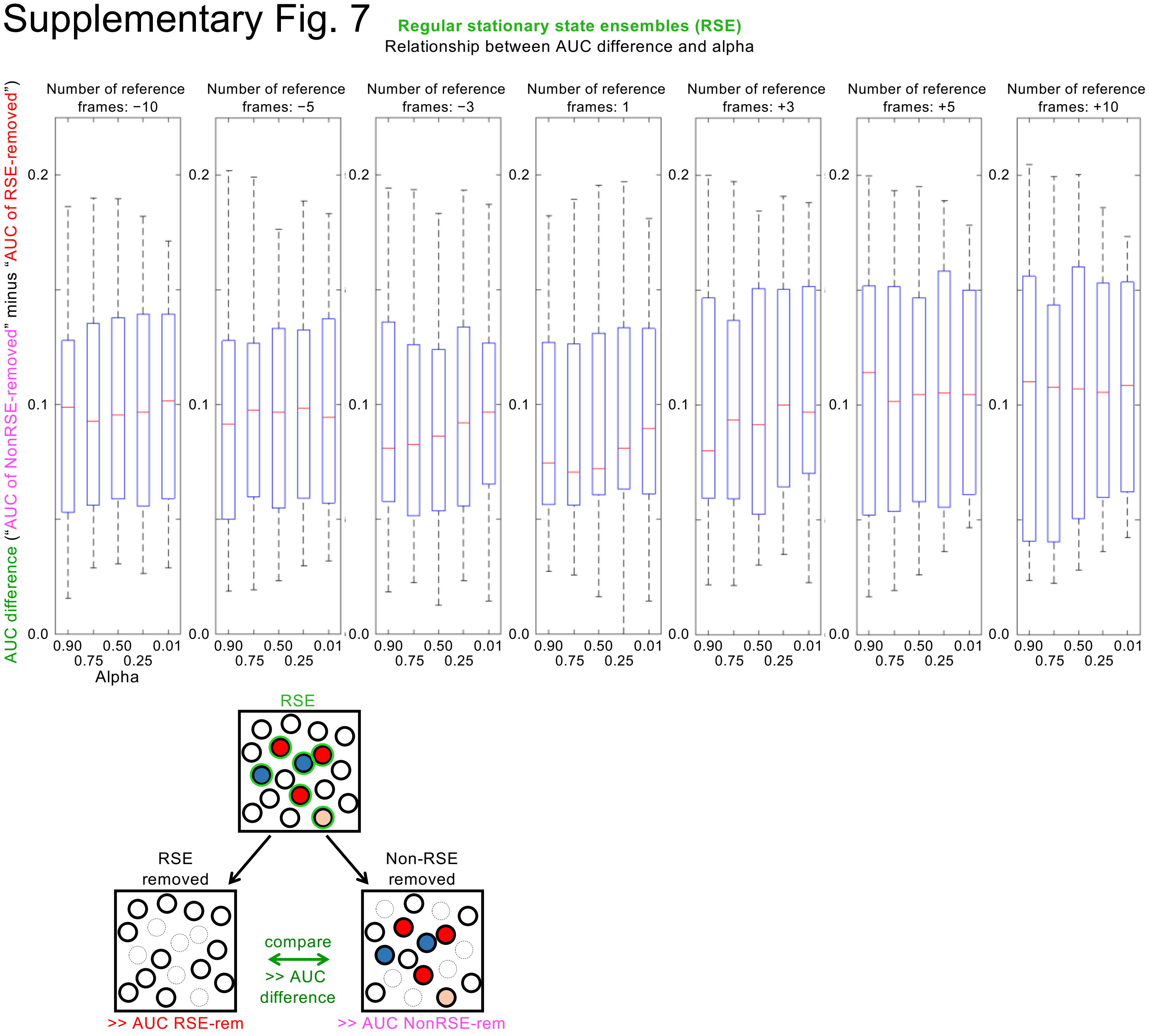

Supplementary Fig. 7. The RS ensemble was not affected by the alpha of the elastic net.

Because optimization of the alpha (hyper parameter for elastic net) was necessary to extract CR ensembles (Fig. 2 and Supplementary Fig. 6), we also investigated the relationship between the alpha and AUC difference for RS ensembles. In addition to various alpha values, we tested various numbers of reference frames (means of the neural activities over several past or future frames were used as neural activity data to estimate a single label at each time-point) to determine the potential difference in the fitting performance. However, we found no significant differences among the various alphas or the different numbers of reference frames, which were evaluated by the Friedman test. Analyses shown in Supplementary Fig. 8 also showed a similar independency of alpha values in the decoding performance of the RS ensembles. According to these results, we decided to consistently use the alpha = 0.75 and to fix number of reference frames to one when modeling RS ensembles. Red bars, median; the bottom and top edges of the box indicate the 25th and 75th percentiles, respectively; whiskers extend to the most extreme data points not considered outliers (outliers were calculated by the "boxplot" function of MATLAB R2014a).

Supplementary Fig. 8

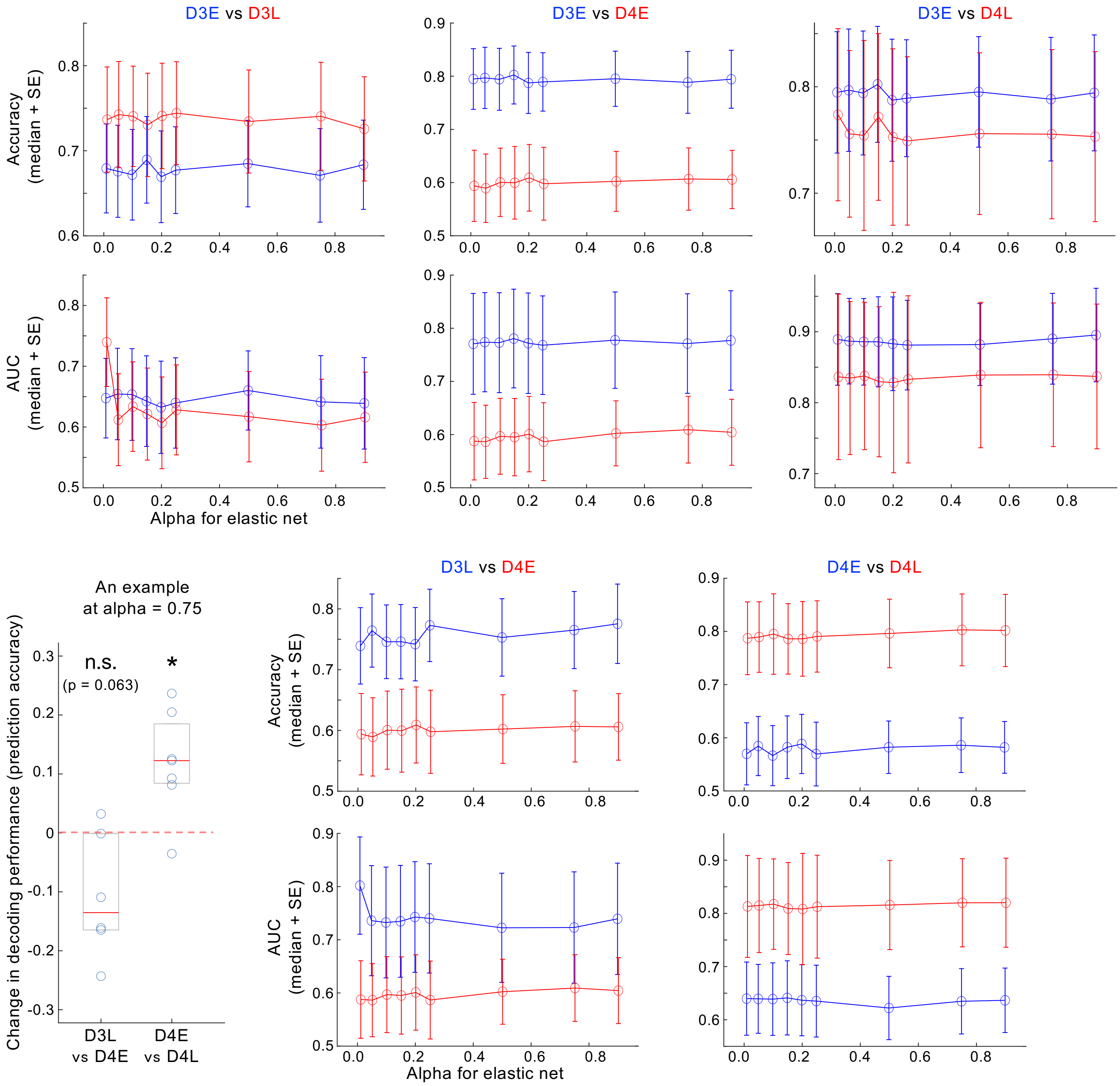

p value at each alpha (paired permutation test)

Alpha	0.01	0.05	0.10	0.15	0.20	0.25	0.50	0.75	0.90
accuracy, D3E vs D3L	0.107	0.219	0.260	0.113	0.250	0.309	0.215	0.244	0.195
accuracy, D3E vs D4E	0.016	0.016	0.016	0.016	0.016	0.016	0.016	0.016	0.016
accuracy, D3E vs D4L	0.531	0.500	0.500	0.531	0.531	0.594	0.469	0.531	0.250
accuracy, D3L vs D4E	0.156	0.094	0.156	0.125	0.219	0.156	0.125	0.063	0.031
accuracy, D4E vs D4L	0.031	0.016	0.078	0.016	0.078	0.031	0.016	0.016	0.031
AUC, D3E vs D3L	0.779	0.273	0.350	0.211	0.354	0.365	0.102	0.322	0.365
AUC, D3E vs D4E	0.219	0.188	0.219	0.219	0.250	0.188	0.188	0.219	0.156
AUC, D3E vs D4L	0.313	0.375	0.688	0.625	0.313	0.375	0.688	0.688	0.563
AUC, D3L vs D4E	0.219	0.563	0.750	0.656	0.750	0.563	0.625	0.563	0.438
AUC, D4E vs D4L	0.094	0.031	0.031	0.031	0.281	0.094	0.031	0.031	0.031

Supplementary Fig. 8. Summary of state-dependent change in decoding performance of RS ensembles to predict locomotion states during the CS+.

To evaluate the decoding performance (predictability of the locomotion states during CS+ presentation by the RS model), we calculated the accuracy and AUC (of the ROC) as described in the Methods. Because there was no difference among the various alphas in any estimates, as shown here and in Supplementary Fig. 7, we fixed the alpha for the RS ensembles at 0.75 for further analyses. For the selected results of alpha = 0.75, the data of individual circuits are also shown in the left middle panel, as in Fig. 3d. We performed imaging experiments on 11 mice; 7 mice were successfully imaged from the same set of neurons on both D3 and D4; data from one of the 7 mice did not meet the modeling criteria for the RSE model on D3 (so N = 10 for D3), while data from all 7 mice met the modeling criteria for the RSE model on D4 (so N = 6 for D4 when compared with D3, and N = 7 for D4E vs D4L). P values at different alphas (calculated by paired permutation test) are also summarized in the table. D3E, D3-early; D3L, D3-late; D4E, D4-early; D4L, D4-late.

Supplementary Fig. 9

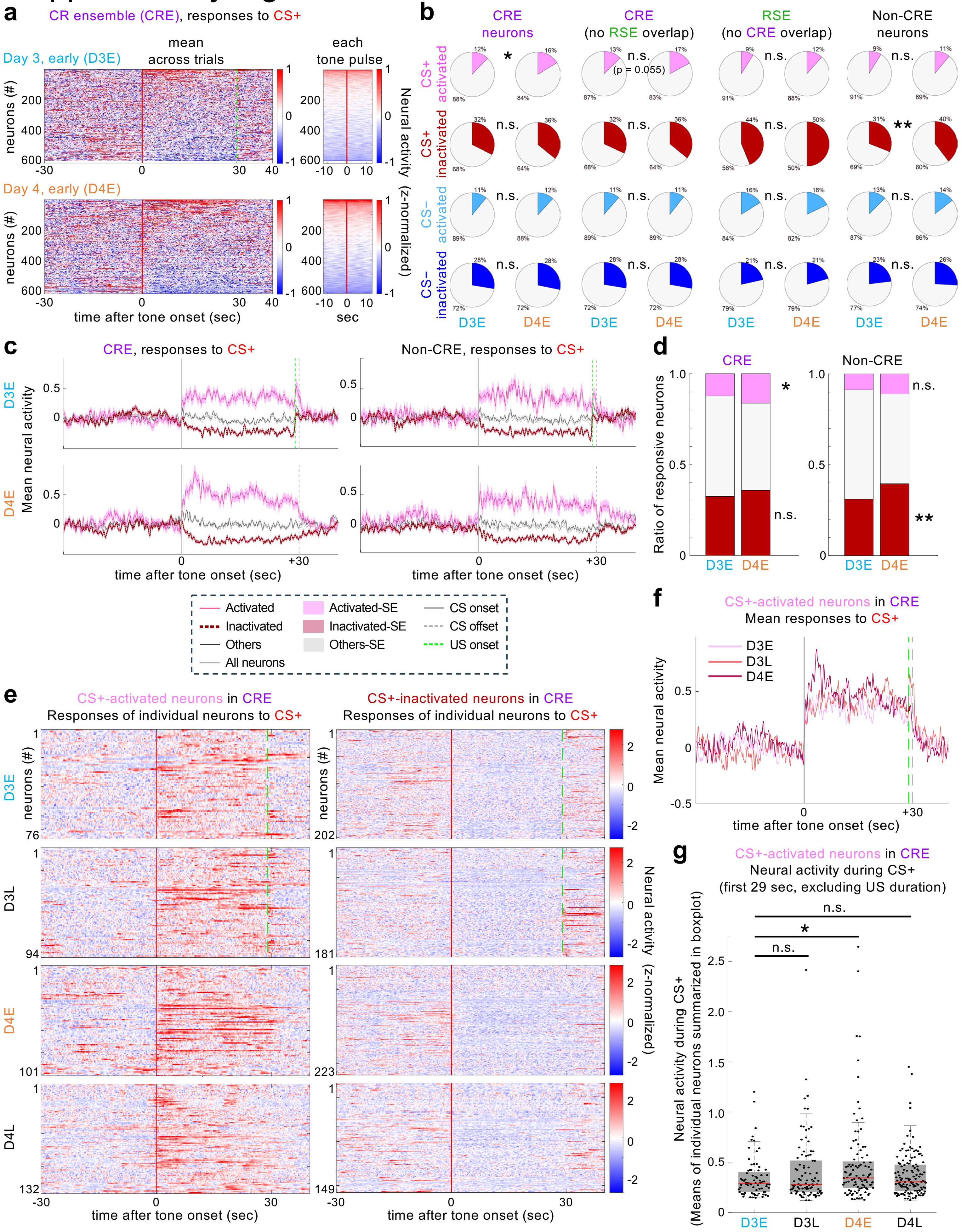

Supplementary Fig. 9. Summary of response profiles of each ensemble to CS+ and CS−

(**a**) Neural activities aligned at the CS onset are ordered by the magnitude of response for 29 sec from the onset of the respective CSs at each temporal phase (D3E, D4E). Different from Supplementary Fig. 5, the mean activity over 3 CS trials, or over 87 onsets of 50-ms tone pulses during the 3 trials (D3E or D4E, respectively) in all individual neurons identified as CR ensemble neurons are plotted, indicating the enhanced responses at D4E compared with D3E. (**b**) Pie charts summarizing changes in CS responses. To categorize the responsiveness of individual neurons, we statistically tested whether the responses of each neuron were significantly different from zero (baseline), and neurons with values significantly higher than the baseline were categorized as "activated" whereas neurons with values significantly lower than the baseline were categorized as "inactivated". See Methods for details. In CR ensemble neurons, CS+-activated neurons were slightly but significantly increased, while no change was observed for other features. On the other hand, in Non-CRE neurons, only CS− inactivated neurons were significantly increased. (**c**) Mean CS+ responses (± s.e.m.) of each category in either CRE or Non-CRE, at each temporal phase (D3E, D4E), are plotted separately. (**d**) Selected features in b and c were re-plotted as stacked bar graphs. The meaning of the color in this panel is the same as the meaning in b. (**e**) CS+ responses of individual neurons at each temporal phase (D3E, D3L, D4E, D4L) are plotted. Among various types of neurons shown in b, CS+-activated and CS+-inactivated neurons in the CRE are selectively shown for details. (**f**) Mean activities of the CS+-activated neurons in the CR ensemble during different temporal phases (D3E, D3L, D4E) are shown to compare the degree of the CS+ responses. D3E and D3L were similar, while D4E showed enhanced responses. (**g**) The mean activities of individual CS+-activated neurons in the CR ensemble during CS+ presentation at each phase (D3E, D3L, D4E, D4L) were plotted and summarized by the boxplot. The analyses were performed with data pooled together from all mice (N = 7 mice; dots represent results of corresponding individual neurons). Statistical comparison between D3E and later phases suggested that the CS+-activated neurons showed significantly higher responses to the CS+ during D4E. Note that this enhancement at the activity level was accompanied by the significantly increased number of the CS+ activated neurons as shown in b and d. At D3L, while some neurons showed higher responses than the D3E, comparison as groups showed no significant difference. Red lines (in a (left two), e) and gray lines (in c and f) indicate the onset of the 30-sec CS+ presentation; Red lines in the right panels in a indicate the onset of the CS+ pulse; green dotted lines (in a, c, e, f) indicate the onset of the US (foot shock) (see Supplementary Fig. 1 for details of the fear conditioning protocol). Red bars in g, median for each group; gray boxes in g indicate 25th and 75th percentiles. A chi-square test was performed for the statistics in b and d; a non-paired comparison (Wilcoxon rank sum test) was performed for g. * $p < 0.05$; ** $p < 0.01$; n.s., not significant. As neural activity, z-normalized $\Delta F/F$ is shown in panels a, c, e-g. D3E, D3-early; D3L, D3-late; D4E, D4-early; D4L, D4-late.

Supplementary Fig. 10

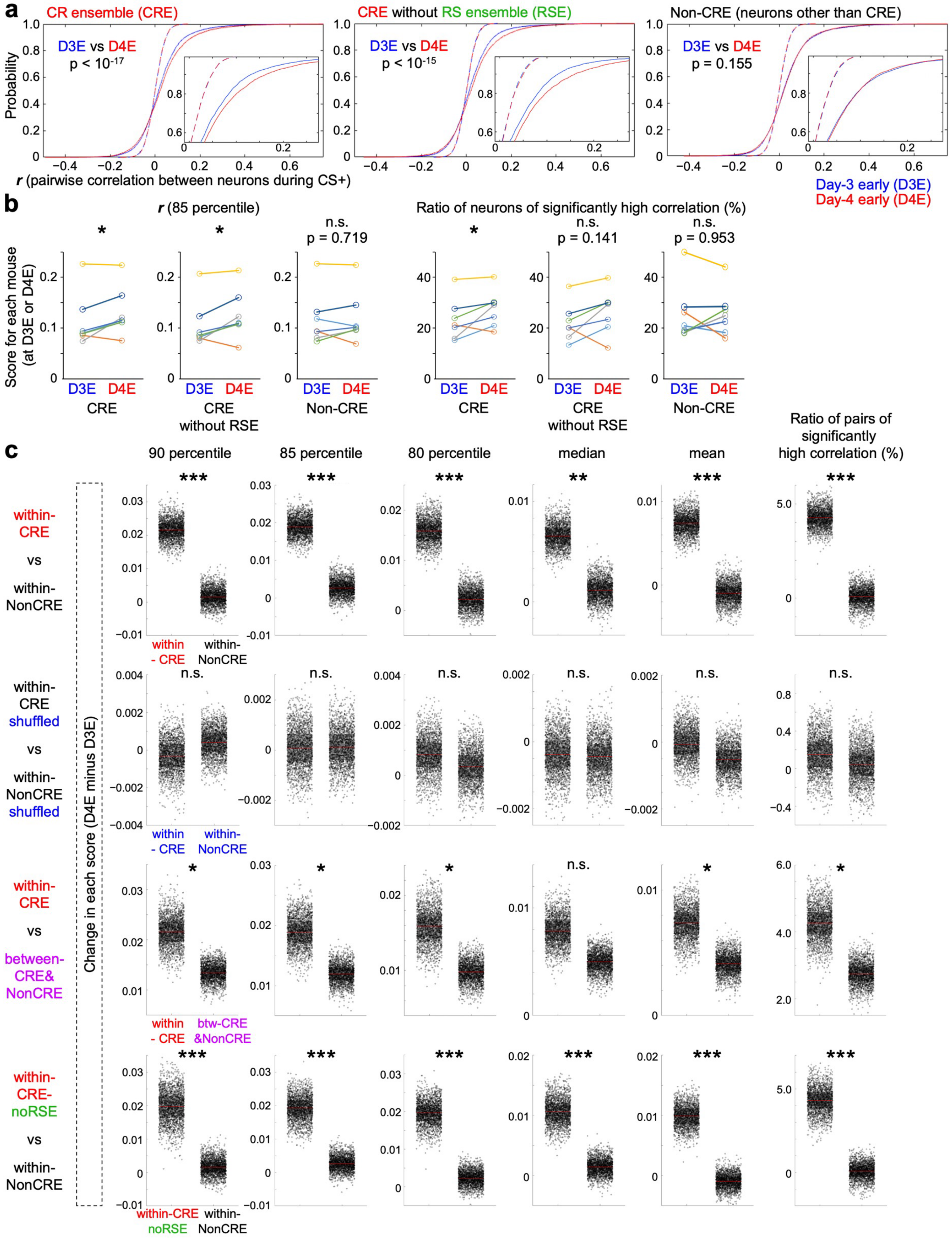

Supplementary Fig. 10. Change in coactivity specifically observed in CR ensembles after fear conditioning.

(**a**) Cumulative curves were drawn for the data pooled by random resampling from all mice (2000 datapoints from each mouse, a total of 14000 datapoints from 7 mice) to visualize the change in coactivity within CR ensemble neurons (CRE), CRE neurons without overlap with RSE neurons (CRE-noRSE), and neurons other than CRE (Non-CRE). Each inset shows a magnification of a part of each graph. Note that this random resampling is different from bootstrap resampling of which results are shown in panel c (See details in the Methods). The results of the statistical comparison between D3E and D4E using a two-sample Kolmogorov-Smirnov test are shown in the respective panels. Dotted lines show the results of shuffled data (See Methods for details). No statistically significant difference between D3E and D4E for the shuffled data in all cases. (**b**) Comparison between D3E and D4E for coactivity-related values was performed with the original (non-shuffled) data. In addition to the systematic analyses based on the bootstrap resampling, of which results are shown in panel c, tests based on the raw data (i.e. representative values from the individual mice) also indicated a significant enhancement of the coactivity specifically in CRE, even though the number of the samples is limited (N = 7 mice). This also demonstrated that the results shown in the panel a and c did not derive from artificially enhanced marginal differences. (**c**) Detailed and systematic analyses of the change in coactivity in dmPFC circuits after the fear conditioning. Changes in the correlation coefficients R (D4E minus D3E) of all chronically observed circuits (N = 7 mice) are plotted as a result of bootstrap resampling (2000 times, shown as 2000 dots in each panel) performed to systematically compare all the raw *r* data of whole mice, for those of CR ensemble neurons (within-CRE) or for those other than CR ensemble neurons (within-NonCRE). Because the panel a indicates that the clear change in *r* between D3E and D4E was observed only for positive correlations specifically in CRE, we statistically tested changes in the 90th, 85th, 80th, and 50th percentiles, mean, and ratio of pairs of the significantly high correlation for each category. We also revealed results for shuffled data and for within-CRE vs between-CRE&NonCRE (coactivity between CRE neurons and Non-CRE neurons), suggesting that enhanced coactivity within the CRE after the fear conditioning was specific. Results of CRE-noRSE were also consistent with those of CRE. A paired permutation test was used for the statistics in b. The data obtained by bootstrap resampling shown in c were statistically analyzed as described in the Methods. * $p < 0.05$; ** $p < 0.01$; *** $p < 0.001$; n.s., not significant. Red bars in c, median; gray boxes in c indicate the 25th and 75th percentiles. D3E, D3-early; D3L, D3-late; D4E, D4-early; D4L, D4-late.

Supplementary Fig. 11

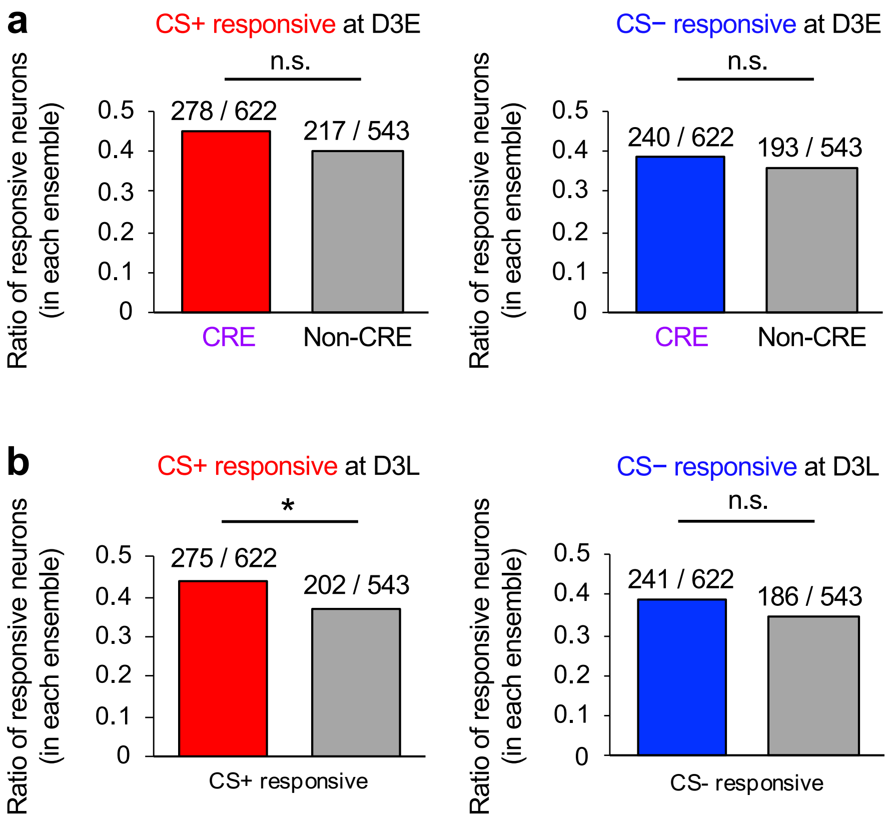

Supplementary Fig. 11. Neural responses to the CS at D3E or D3L and integration of these neurons into the CR ensemble on the day after the fear conditioning.

(**a**) Neuronal responses to the CSs during D3E were not significantly implicated in whether the neurons became included in the CRE on the day after fear conditioning. For the neurons responding to the CSs at D3E (CS+-responsive, n = 495; CS− -responsive, n = 433), we statistically evaluated whether they became involved in CRE (n = 622 neurons in total) or Non-CRE (n = 543). The CS+-responsiveness (left) and CS−-responsiveness (right) were analyzed separately. Fisher's exact test was used as in the case of the US (shown in Fig. 4h). There was no significant difference in these cases. (**b**) On the other hand, neurons responding to the CS+ at D3L (i.e., after the repeated CS+-US pairing) were predominantly involved in the CRE on D4, which was statistically confirmed by Fisher's exact test. There was no statistical significance in the case of CS−-responsiveness. At D3L, CS+-responsive neurons, n = 477 in total; CS− -responsive neurons, n = 427. All the analyses in this figure were performed with pooled data from all of the mice (N = 7 mice), as we did in Fig. 4h. * $p < 0.05$; n.s., not significant.

Supplementary Fig. 12

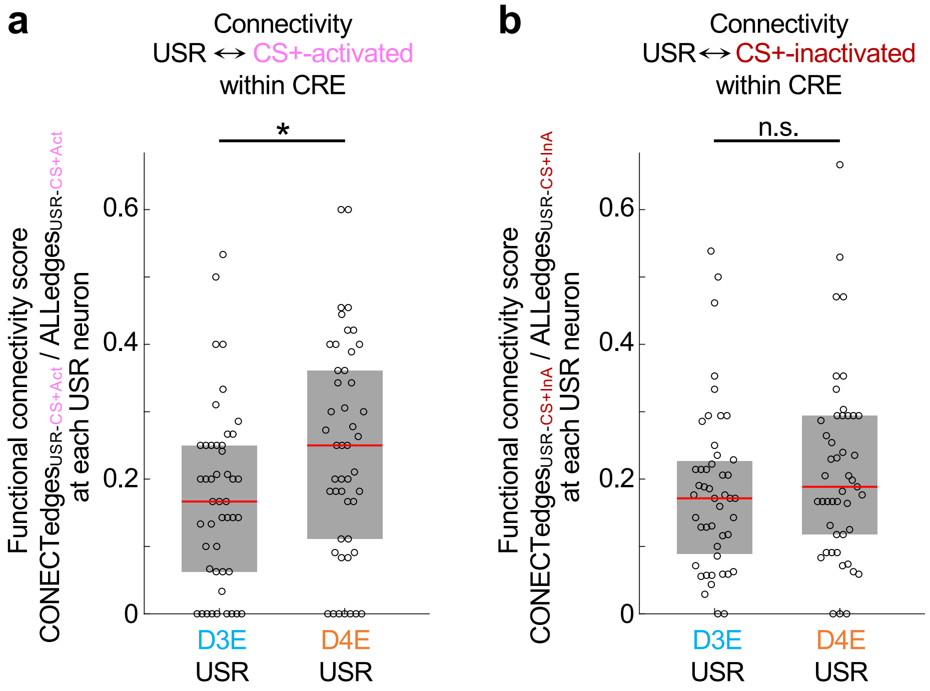

Supplementary Fig. 12. Enhanced functional connectivity between CS+-activated neurons and US-responsive neurons within the CR ensemble on the day after the fear conditioning.

The functional connectivity of US-responsive neurons (USR) to CS+-responsive (activated [**a**] or inactivated [**b**]) neurons within the CR ensemble was compared between D3E and D4E. The functional connectivity score for each neuron was calculated as the ratio (the number of the functional connections from one USR neuron to all (other) CS+-responsive neurons was divided by all possible connections (i.e., number of edges) between the USR neuron and all (other) CS-responsive neurons). Functional connectivity between the US-responsive neurons and the CS+-activated neurons within the CR ensemble was significantly enhanced after the fear conditioning (a), while there was no significant difference for the CS+-inactivated neurons (b). The analyses were performed with data pooled together from all mice (N = 7 mice; dots represent results of corresponding individual neurons). The number of USR neurons integrated into the CR ensemble was 47 (as shown in Fig. 4h). Within the CR ensemble, the number of the neurons for CS+-activated at D3E, 76; CS+-activated at D4E, 101; CS+-inactivated at D3E, 202; CS+-inactivated at D4E, 223. Red bars in a and b, median; gray boxes in a and b indicate the 25th and 75th percentiles. The Wilcoxon signed-rank test (a paired test) was used for the statistical comparison. * $p < 0.05$; n.s., not significant.

Supplementary Fig. 13

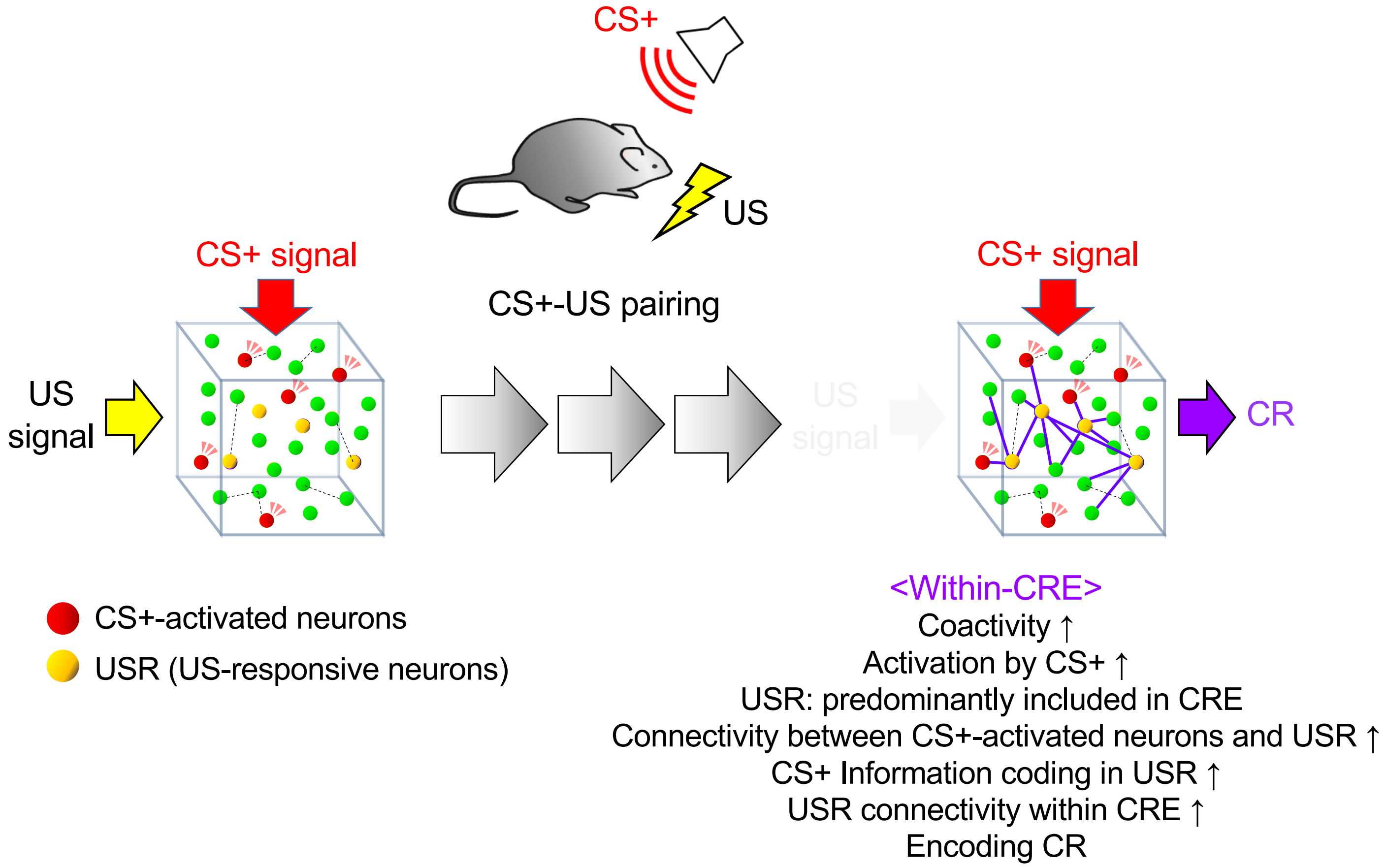

Supplementary Fig. 13. Summary of the present study.

We developed a pipeline for longitudinal two-photon imaging and computational dissection of the neural population, which allowed us to investigate learning-dependent dynamic modulation of functional connectivity and population coding for associative fear learning in the dmPFC network. Neurons activated by the US during fear conditioning (USR, yellow circle) were consequently and predominantly integrated into the CR ensemble (CRE, a group of circles inter-connected by purple lines), a neural population encoding the CR, as a result of the associative fear learning. We also demonstrated that; after fear conditioning, the CR ensemble neurons became coactivated together and more activated by the CS+; the functional connectivity between the CS+-activated neurons (red circle) and the USR was enhanced in the CRE (but not in the outside network), which was accompanied by the enhanced information coding for the CS+ in the USR; and the USR possessed significantly higher functional connectivity within the entire CRE. The CR ensemble encoded the CR information and was distinct from the regular motor-coding neurons. Conclusively, these results visualized the possible signal flow from the CS+ to the CS+-activated neurons in the CRE, then to the USR, and further to the entire CRE, most of which association or responsiveness was enhanced as a result of the repeated CS+ and US pairings.

These results in the present study suggest that the eventual network stemming from the USR gained typical features of pattern completion cells of the CRE, which are supposed to work as a hub in the prefrontal networks to dominantly relay the CS+ information and promote the CR, underlying the associative fear memory.

Supplementary Fig. 14

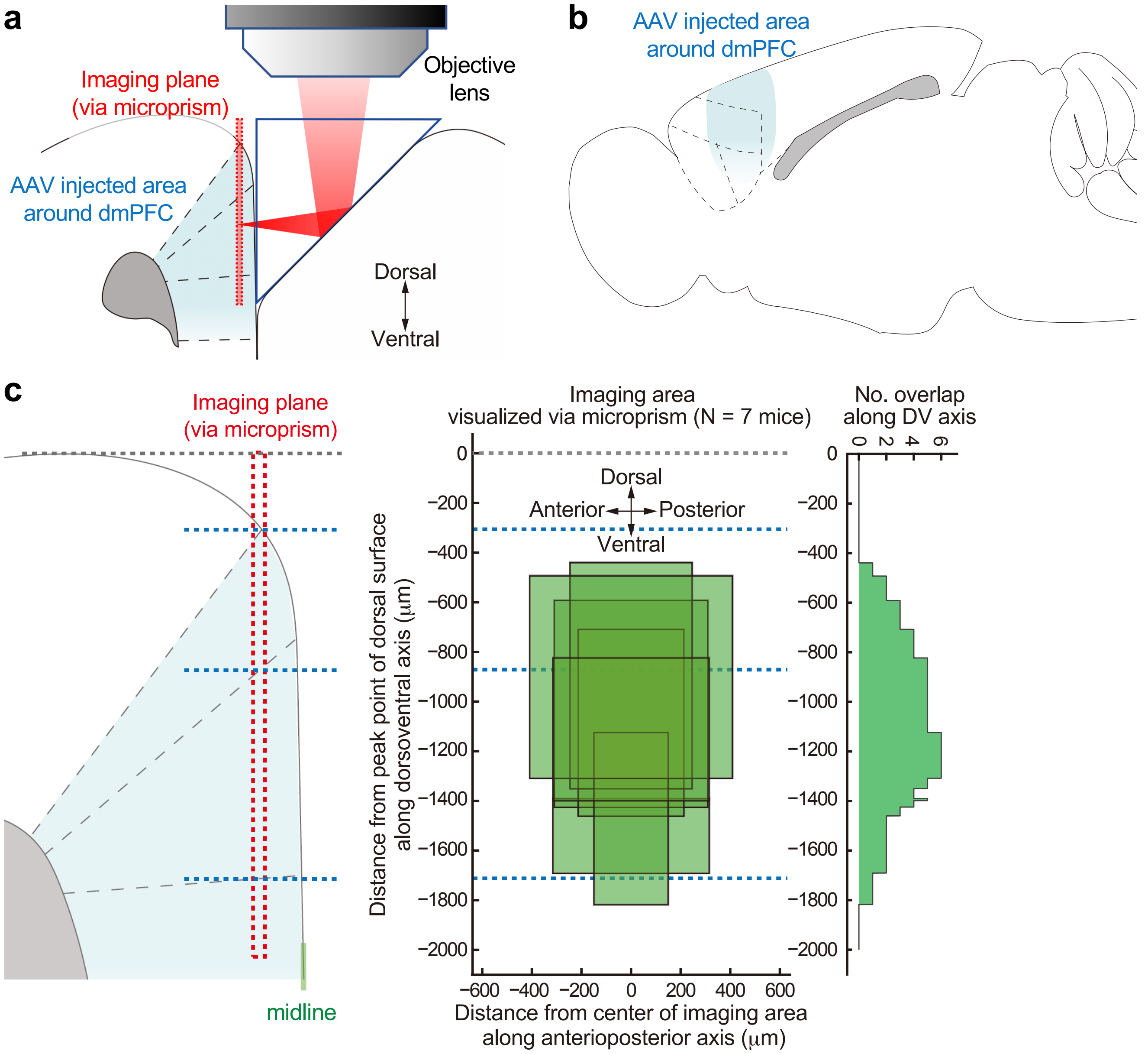

Supplementary Fig. 14. Summary of imaging regions around the dmPFC

(**a**) Coronal section illustrating how neural activity in the dorsal part of the medial prefrontal cortex (dmPFC) was recorded. We injected the AAV1/CamKII.GCaMP6f to cover a wide region of the medial prefrontal cortex (pale blue; see Methods for details), and the GCaMP6f expressed in the dorsal part was visualized by two-photon microscopy through an implanted 2x2 mm microprism. This imaging method allowed us to measure the position of each imaging area (i.e., depth from the dorsal surface of the brain and distance from the midline). We recorded the neural activity of the superficial midline layer of the dmPFC (red-dotted line) approximately 150–200 µm from the pial surface along the midline. (**b**) Sagittal section including the superficial layer of the dmPFC and the AAV-injected area (pale blue). (**c**) Summary of the imaging regions in mice recorded on both D3 and D4 (N = 7). (left) Magnified view of panel a. Gray bold dotted line indicates the peak point of the dorsal surface of the brain. Regions between pale blue dotted lines (and gray dashed lines) correspond to the anterior cingulate area (ACA, top) and prelimbic area (PL, bottom), respectively, according to the Allen Brain Atlas. (middle) Each green square indicates the location and spatial distribution of the neural population recorded by two-photon imaging in each mouse. (right) Number of overlapping imaged areas between multiple mice (N = 7) along the dorsoventral (DV) axis. In the middle and right panels, the vertical axes indicate the distance from the peak point of the dorsal surface of the brain, and gray and pale blue vertical lines correspond to the positions in the left panel.
