# Supplementary material for: Activity-dependent organization of prefrontal hub-networks for associative learning and signal transformation
Source: Nat Commun. 2023 Oct 6;14:5996. doi: 10.1038/s41467-023-41547-5 (PMC10558457; doi:10.1038/s41467-023-41547-5)
Supplement: Supplementary file 2 — Description of Additional Supplementary Files [file 41467_2023_41547_MOESM2_ESM.pdf]

Description of additional supplementary files

Supplementary Movie 1. An example of spontaneous activities in dmPFC on day 4 (the day after the fear conditioning), detected by changes in GCaMP6f signals in a field of view. (left) original GCaMP6f signal, (middle) baseline subtracted signal shown in magenta, (right) baseline subtracted signal is shown in magenta over a background of the baselineimage shown in gray.

Supplementary Movie 2. An example of GCaMP6f signals on day 3, during fear conditioning. Baseline subtracted signal is shown in magenta, and merged over the baseline-image shown in gray. The timings of the CS and US presentation were indicated at the left upper corner in the movie. The CS and US presentation did not disturb the image acquisition.
