# Supplementary material for: Activity-dependent organization of prefrontal hub-networks for associative learning and signal transformation
Source: Nat Commun. 2023 Oct 6;14:5996. doi: 10.1038/s41467-023-41547-5 (PMC10558457; doi:10.1038/s41467-023-41547-5)
Supplement: Supplementary file 5 — Reporting Summary [file 41467_2023_41547_MOESM5_ESM.pdf]

nature portfolio

Corresponding author(s): Masakazu Agetsuma

Last updated by author(s): Aug 7, 2023

Reporting Summary

Nature Portfolio wishes to improve the reproducibility of the work that we publish. This form provides structure for consistency and transparency in reporting. For further information on Nature Portfolio policies, see our Editorial Policies and the Editorial Policy Checklist.

Statistics

For all statistical analyses, confirm that the following items are present in the figure legend, table legend, main text, or Methods section.

n/a	Confirmed	
☐	☒	The exact sample size (*n*) for each experimental group/condition, given as a discrete number and unit of measurement
☐	☒	A statement on whether measurements were taken from distinct samples or whether the same sample was measured repeatedly
☐	☒	The statistical test(s) used AND whether they are one- or two-sided<br>*Only common tests should be described solely by name; describe more complex techniques in the Methods section.*
☒	☐	A description of all covariates tested
☐	☒	A description of any assumptions or corrections, such as tests of normality and adjustment for multiple comparisons
☐	☒	A full description of the statistical parameters including central tendency (e.g. means) or other basic estimates (e.g. regression coefficient) AND variation (e.g. standard deviation) or associated estimates of uncertainty (e.g. confidence intervals)
☐	☒	For null hypothesis testing, the test statistic (e.g. *F*, *t*, *r*) with confidence intervals, effect sizes, degrees of freedom and *P* value noted<br>*Give P values as exact values whenever suitable.*
☒	☐	For Bayesian analysis, information on the choice of priors and Markov chain Monte Carlo settings
☒	☐	For hierarchical and complex designs, identification of the appropriate level for tests and full reporting of outcomes
☐	☒	Estimates of effect sizes (e.g. Cohen's *d*, Pearson's *r*), indicating how they were calculated

Our web collection on statistics for biologists contains articles on many of the points above.

Software and code

Policy information about availability of computer code

Data collection	Data of imaging and simultaneous behavioral experiments were collected by Labview 2015/2018 (National Instruments) and FV30S-SW image acquisition and processing software (Olympus) as described in the manuscript.
Data analysis	For data analyses in the present study, commercial software and publicly available codes were used (details are described in the method section of the manuscript). For the statistical analysis, we used MATLAB R2014a and R2019b (MathWorks, Natick, MA). Graphs were produced by MATLAB R2014a/R2019b (MathWorks) and Excel (Microsoft). Raw images of the GcaMP6f signals in the dmPFC were processed to correct for brain motion artifacts, using the publicly available code for the enhanced correlation coefficient image alignment algorithm (Evangelidis and Psarakis, 2008; https://jp.mathworks.com/matlabcentral/fileexchange/27253-ecc-image-alignment-algorithm-image-registration). The ROIs for the detection of neural activity were automatically selected using a constrained nonnegative matrix factorization algorithm in MATLAB R2014a/R2019b, and the code is also publicly available (Pnevmatikakis et al., 2016; https://github.com/flatironinstitute/CaImAn-MATLAB). For inferring the spike probabilities from the dF/F as an alternative estimate of neuronal activation, the publicly available code “constrained_foopsi.m” (Pnevmatikakis et al., 2016; https://github.com/flatironinstitute/CaImAn-MATLAB) was used. For the elastic net, we used the “lassoglm” function of MATLAB R2019b. For the conditional random field (CRF) modeling, we used the publicly available code developed previously (Carrillo-Reid et al., 2019; Carrillo-Reid et al., 2021; https://github.com/hanshuting/graph_ensemble; https://github.com/darikoneil/Identification-of-Pattern-Completion-Neurons).

For manuscripts utilizing custom algorithms or software that are central to the research but not yet described in published literature, software must be made available to editors and reviewers. We strongly encourage code deposition in a community repository (e.g. GitHub). See the Nature Portfolio guidelines for submitting code & software for further information.

Data

Policy information about availability of data

All manuscripts must include a data availability statement. This statement should provide the following information, where applicable:

- Accession codes, unique identifiers, or web links for publicly available datasets
- A description of any restrictions on data availability
- For clinical datasets or third party data, please ensure that the statement adheres to our policy

Source data files are provided with this paper. The data that support the findings of this study are also available from the corresponding author upon reasonable request. The anatomical information in the Allen Brain Atlas (https://atlas.brain-map.org/) was used for the anatomical description, determination of the virus injection area, and evaluation of the recorded brain regions.

Human research participants

Policy information about studies involving human research participants and Sex and Gender in Research.

Reporting on sex and gender	N/A
Population characteristics	N/A
Recruitment	N/A
Ethics oversight	N/A

Note that full information on the approval of the study protocol must also be provided in the manuscript.

Field-specific reporting

Please select the one below that is the best fit for your research. If you are not sure, read the appropriate sections before making your selection.

☒ Life sciences ☐ Behavioural & social sciences ☐ Ecological, evolutionary & environmental sciences

For a reference copy of the document with all sections, see nature.com/documents/nr-reporting-summary-flat.pdf

Life sciences study design

All studies must disclose on these points even when the disclosure is negative.

Sample size	Sample sizes (numbers of neurons and animals) were based on work in previous publications (Ref 14, 16, 41, 42), and no statistical tests were used to predetermine sample sizes.
Data exclusions	No mice were excluded. Inclusion criteria for recorded neurons were based on the algorithm as described in the previous studies and in the method section of the present study.
Replication	Behavioral experimental settings of the fear conditioning, i.e., tones and foot shocks, and those of the two-photon imaging, were used as replicates independently in all mice. To validate the reproducibility and reliability of the obtained results, we tested various parameters and the ways to verify the results as shown in the results (e.g., in Figs. S2, S6-8, and S10), all of which indicated very similar results, specificities, and conclusions. We performed tests based on bootstrap resampling as replicates to evaluate the statistical significance between the two groups systematically.
Randomization	To verify the neural coactivity measurement, we used not only the original data but also the shuffled data, where the activity of each neuron was preserved but the temporal order was randomly shuffled neuron by neuron. To systematically estimate representative values (e.g. mean or median) of each mouse or each group where the number of recorded neurons in each field view varied, we performed bootstrap resampling as explained in the method section of the present study.
Blinding	For the behavioral experiments and virus injections, blinding was not relevant because all behavioral experiments were controlled by computer systems, and the virus injections were performed with constant settings and thresholds. The investigators were blinded to group allocation during all data collection and analysis since they were automatically performed with constant settings and thresholds.

Reporting for specific materials, systems and methods

We require information from authors about some types of materials, experimental systems and methods used in many studies. Here, indicate whether each material, system or method listed is relevant to your study. If you are not sure if a list item applies to your research, read the appropriate section before selecting a response.

Materials & experimental systems

n/a	Involved in the study
[x]	[ ] Antibodies
[x]	[ ] Eukaryotic cell lines
[x]	[ ] Palaeontology and archaeology
[ ]	[x] Animals and other organisms
[x]	[ ] Clinical data
[x]	[ ] Dual use research of concern

Methods

n/a	Involved in the study
[x]	[ ] ChIP-seq
[x]	[ ] Flow cytometry
[x]	[ ] MRI-based neuroimaging

Animals and other research organisms

Policy information about studies involving animals; ARRIVE guidelines recommended for reporting animal research, and Sex and Gender in Research

Laboratory animals	Male C57BL/6 mice housed under a 12-h light/dark cycle with free access to food and water in a temperature-controlled environment (22–24 °C and 30–60 % humidity) were used for all experiments. Experiments were performed during the dark cycle (i.e. when mice were normally awake) using single-housed mice. Mice at 3–6 months of age were used for the behavioral and imaging experiments.
Wild animals	No wild animal was used in the present study.
Reporting on sex	All mice (N=33) used in the present study were male.
Field-collected samples	No field collected sample was used in the present study.
Ethics oversight	All animal experiments were carried out in accordance with the Institutional Guidance on Animal Experimentation and with permission from the Animal Experiment Committee of Osaka University (authorization number: 3348), or in accordance with National Institutes of Health guidelines and approved by the National Institute for Physiological Sciences Animal Care and Use Committee (approval number 18A102).

Note that full information on the approval of the study protocol must also be provided in the manuscript.
